# Higher-order phylogeny of modern birds (Theropoda, Aves: Neornithes) based on comparative anatomy. II. Analysis and discussion

**DOI:** 10.1111/j.1096-3642.2006.00293.x

**Published:** 2007-01-01

**Authors:** BRADLEY C LIVEZEY, RICHARD L ZUSI

**Affiliations:** 1 Section of Birds, Carnegie Museum of Natural History 4400 Forbes Avenue, Pittsburgh, PA 15213-4080, USA; 2 Division of Birds, National Museum of Natural History Washington, DC 20013-7012, USA

**Keywords:** Aves, cladistics, classification, convergence, homology, morphology, ontogeny, palaeontology, phylogenetics, Neornithes, taxonomy

## Abstract

In recent years, avian systematics has been characterized by a diminished reliance on morphological cladistics of modern taxa, intensive palaeornithogical research stimulated by new discoveries and an inundation by analyses based on DNA sequences. Unfortunately, in contrast to significant insights into basal origins, the broad picture of neornithine phylogeny remains largely unresolved. Morphological studies have emphasized characters of use in palaeontological contexts. Molecular studies, following disillusionment with the pioneering, but non-cladistic, work of Sibley and Ahlquist, have differed markedly from each other and from morphological works in both methods and findings. Consequently, at the turn of the millennium, points of robust agreement among schools concerning higher-order neornithine phylogeny have been limited to the two basalmost and several mid-level, primary groups. This paper describes a phylogenetic (cladistic) analysis of 150 taxa of Neornithes, including exemplars from all non-passeriform families, and subordinal representatives of Passeriformes. Thirty-five outgroup taxa encompassing Crocodylia, predominately theropod Dinosauria, and selected Mesozoic birds were used to root the trees. Based on study of specimens and the literature, 2954 morphological characters were defined; these characters have been described in a companion work, approximately one-third of which were multistate (i.e. comprised at least three states), and states within more than one-half of these multistate characters were ordered for analysis. Complete heuristic searches using 10 000 random-addition replicates recovered a total solution set of 97 well-resolved, most-parsimonious trees (MPTs). The set of MPTs was confirmed by an expanded heuristic search based on 10 000 random-addition replicates and a full ratchet-augmented exploration to ascertain global optima. A strict consensus tree of MPTs included only six trichotomies, i.e. nodes differing topologically among MPTs. Bootstrapping (based on 10 000 replicates) percentages and ratchet-minimized support (Bremer) indices indicated most nodes to be robust. Several fossil Neornithes (e.g. Dinornithiformes, Aepyornithiformes) were placed within the ingroup a posteriori either through unconstrained, heursitic searches based on the complete matrix augmented by these taxa separately or using backbone-constraints. Analysis confirmed the topology among outgroup Theropoda and achieved robust resolution at virtually all levels of the Neornithes. Findings included monophyly of the palaeognathous birds, comprising the sister taxa Tinamiformes and ratites, respectively, and the Anseriformes and Galliformes as monophyletic sister-groups, together forming the sister-group to other Neornithes exclusive of the Palaeognathae (Neoaves). Noteworthy inferences include: (i) the sister-group to remaining Neoaves comprises a diversity of marine and wading birds; (ii) Podicipedidae are the sister-group of Gaviidae, and not closely related to the Phoenicopteridae, as recently suggested; (iii) the traditional Pelecaniformes, including the shoebill (*Balaeniceps rex*) as sister-taxon to other members, are monophyletic; (iv) traditional Ciconiiformes are monophyletic; (v) Strigiformes and Falconiformes are sister-groups; (vi) Cathartidae is the sister-group of the remaining Falconiformes; (vii) Ralliformes (Rallidae and Heliornithidae) are the sister-group to the monophyletic Charadriiformes, with the traditionally composed Gruiformes and Turniciformes (Turnicidae and Mesitornithidae) sequentially paraphyletic to the entire foregoing clade; (viii) *Opisthocomus hoazin* is the sister-taxon to the Cuculiformes (including the Musophagidae); (ix) traditional Caprimulgiformes are monophyletic and the sister-group of the Apodiformes; (x) Trogoniformes are the sister-group of Coliiformes; (xi) Coraciiformes, Piciformes and Passeriformes are mutually monophyletic and closely related; and (xii) the Galbulae are retained within the Piciformes. Unresolved portions of the Neornithes (nodes having more than one most-parsimonious solution) comprised three parts of the tree: (a) several interfamilial nodes within the Charadriiformes; (b) a trichotomy comprising the (i) Psittaciformes, (ii) Columbiformes and (iii) Trogonomorphae (Trogoniformes, Coliiformes) + Passerimorphae (Coraciiformes, Piciformes, Passeriformes); and (c) a trichotomy comprising the Coraciiformes, Piciformes and Passeriformes. The remaining polytomies were among outgroups, although several of the highest-order nodes were only marginally supported; however, the majority of nodes were resolved and met or surpassed conventional standards of support. Quantitative comparisons with alternative hypotheses, examination of highly supportive and diagnostic characters for higher taxa, correspondences with prior studies, complementarity and philosophical differences with palaeontological phylogenetics, promises and challenges of palaeogeography and calibration of evolutionary rates of birds, and classes of promising evidence and future directions of study are reviewed. Homology, as applied to avian examples of apparent homologues, is considered in terms of recent theory, and a revised annotated classification of higher-order taxa of Neornithes and other closely related Theropoda is proposed. © 2007 The Linnean Society of London, *Zoological Journal of the Linnean Society*, 2007, **149**, 1–95.

## INTRODUCTION

‘But as far as the problem of the relationship of the orders of birds is concerned, so many distinguished investigators have labored in this field in vain, that little hope is left for spectacular break-throughs.’ (Stresemann, 1959: 277)

‘It must be remembered that the basic avian structure was determined at an early stage in the evolutionary history of birds because of the rigorous limitations placed upon a flying vertebrate. Consequently, adaptations in the birds have been along lines that are not always indicated by the details of anatomy, a fact that makes these vertebrates highly interesting to the student of recent animals but difficult subjects for the palaeontologist.’ (Colbert, 1980: 187)

### Maturation of avian phylogenetics

#### Confines of tradition

The opening quotation from Colbert (1980) clearly articulates a fundamental assumption of functional constraint under which many avian systematists laboured for more than a century (Wyles *et al.*, 1983). Apparently retarded rates of morphological and molecular change (Primmer & Ellegren, 1998; Stanley & Harrison, 1999) strongly influenced evolutionary theory as applied to birds, e.g. prompting assessment of phylogenetic principles for morphologically ‘uniform’ groups (Bock, 1963a). This duality – higher-order diversity defying phylogenetic inference and study of morphological variation lacking unified phylogenetic focus – was influential during the last century.

Avian systematics has followed a general tri-phasic pattern: (i) a descriptive period – epitomized by the landmark works by Huxley (1867), Fürbringer (1888) and Gadow (1892, 1893), in which early classifications of the period were based solely on anatomical evidence and distinctly informal in nature (Seebohm, 1888, 1889, 1890a, b, c, 1895; Clark, 1901); (ii) a comparative (multitaxic) period – typically confined to single skeletal elements, articulations, limbs or organ systems (e.g. Bock, 1959, 1960a, b; Cracraft, 1968; Ames, 1971); and (iii) a phylogenetic period – the primary literature considered herein.

Important advances in avian systematics have been typified by studies focused on key extant taxa – e.g. *Balaeniceps rex* (Cottam, 1957) and *Pedionomus torquatus* (Olson & Steadman, 1981) – or promising aspects of anatomy – e.g. appendicular myology (Garrod, 1873a, 1874) – as well as a few broad surveys of modern taxa (Cracraft, 1986; Cracraft & Mindell, 1989). Regardless of method, however, scale of avian phylogenetics seldom exceeded single orders prior to 1990, when palaeontological finds revived such broad systematic endeavours. From the earliest years of avian systematics, ornithologists were attracted to taxa posing confusing combinations of characters, and a few systematists showed an uncanny recognition of taxa that were key to problems concerning larger groups (Table 1).

**Table 1:** Selected references concerning neognathous Neornithes qualifying as perennial problems of higher-order (supra-ordinal) systematics (see Sibley & Ahlquist, 1972, 1981, 1990)

Taxon	Alternative proposals	References
Gaviiformes	Podicipediformes, Procellariiformes	Stresemann (1934); Verheyen (1959a, 1961); Storer (1956, 1971a, b)
Podicipediformes	Gaviiformes, Pelecaniformes, Gruiformes, Heliornithidae, Phoenicopteridae	Stresemann (1934); Verheyen (1959b, 1961); Tyler (1969); Olson (1985); Van Tuinen *et al*. (2001); Storer (2002); Mayr (2004a)
Pelecaniformes	Polyphyly (multiple topologies)	Beddard (1897); Chandler (1916); Simonetta (1963); Hedges & Sibley (1994); Bourdon, Boya & Iarochène (2005)
Balaenicipitidae	Ciconiiformes, Pelecaniformes	Parker (1860, 1861); Reinhardt (1860, 1862); Bartlett (1861); Giebel (1873); Beddard (1888); Shufeldt (1901a); Mitchell (1913); Böhm (1930); Cottam (1957); Feduccia (1977a); Cracraft (1985); Mayr (2003a); Mayr & Clarke (2003)
Scopidae	Ciconiiformes, Pelecaniformes	Beddard (1884); Shufeldt (1901a); Mayr (2003a)
Falconimorphae	Polyphyly	Fürbringer (1888); Gadow (1892); Sibley & Ahlquist (1990)
Cathartidae	Falconiformes, Ciconiiformes	Ligon (1967); Cracraft & Rich (1972); Emslie (1988); Avise, Nelson & Sibley (1994a); Helbig & Seibold (1995)
Phodilidae	Tytonidae, Strigidae	Milne-Edwards (1878a); Beddard (1890); Shufeldt (1900); Miller (1965); Hoff (1966); Marshall (1966)
Phoenicopteridae	Anseriformes, Ciconiiformes, Charadriiformes	Gadow (1877); Weldon (1883); Parker (1889a); Shufeldt (1889a, 1901b); Chandler (1916); Feduccia (1976, 1977b); Livezey (1997a, b, 1998a)
Turnicidae	Gruiformes, Galliformes; indeterminate, basal Neornithines	Parker (1862); Ogilvie-Grant (1889); Gadow (1893); Lowe (1923); Livezey (1998b); Rotthowe & Starck (1998)
Mesitornithidae	Gruiformes, Cuculiformes	Bartlett (1877); Milne-Edwards (1878b); Forbes (1882); Gadow (1893); Lowe (1924); Livezey (1998b); Mayr & Ericson (2004)
Pedionomidae	Gruiformes, Charadriiformes	Gadow (1891a); Bock & McEvey (1969); Olson & Steadman (1981); Livezey (1998b)
Rhynochetidae	Ardeiformes, Gruiformes	Bartlett (1862); Parker (1869); Murie (1871); Beddard (1891); Mitchell (1915); Steinbacher (1968); Livezey (1994, 1998b)
Opisthocomidae	Tinamidae, Ratitae, Galliformes, Cuculiformes, Columbidae, Pteroclide, Rallidae, Otididae, Coliidae	Perrin (1875); Garrod (1879); Von Nathusius (1881); Beddard (1889); Gadow (1891b); Parker (1891); Mitchell (1896); Shufeldt (1918); Böker (1929); Barnikol (1953); Parsons (1954); Verheyen (1956a); Sibley & Ahlquist (1973); Avise *et al*. (1994b); Hackett *et al*. (1995); Hedges *et al*. (1995); Hughes & Baker (1999); Johansson *et al*. (2001); Mayr & Clarke (2003); Sorenson *et al*. (2003)
Pteroclidae	Columbiformes, Galliformes, Charadriiformes	Parker (1862); Elliot (1878); Gadow (1882); Shufeldt (1901c); Chandler (1916); Stegmann (1957, 1959); Fjeldså (1976)
Caprimulgiformes	Paraphyly or polyphyly, notably Aegothelidae, Steatornithidae	Garrod (1873b); Shufeldt (1885); Beddard (1886); Parker (1889b); Bühler (1970); Johansson *et al*. (2001); Mayr (2002a)
Trochilidae	Apodidae, Passeriformes	Lowe (1939); Chandler (1916); Cohn (1968)
Coliidae	Cuculiformes, Coraciiformes, Indicatoridae, Caprimulgiformes	Murie (1872a); Garrod (1876); Verheyen (1956b); Sibley & Ahlquist (1972); Berman & Raikow (1982); Espinosa de los Monteros (2000)
Trogonidae	Coliidae, Cuculiformes, Coraciiformes	Forbes (1881); Espinosa de los Monteros (1998, 2000); Mayr (2003b)

Percy Roycroft Lowe (British Museum), despite an idiosyncratic view of ontogeny in evolution (Livezey, 1995a) and pre-Hennigian concepts of phylogenetic reconstruction, undertook early and under-appreciated attempts to resolve the phylogenetic positions of problematic avian groups. Early works by Lowe emphasized the vexing Charadriiformes and allied Gruiformes (Lowe, 1922, 1923, 1924, 1925, 1931a, b), the ratites (Lowe, 1928, 1930, 1942, 1944a), ‘primitive’ characters of Sphenisciformes (Lowe, 1933), characters of *Archaeopteryx* possibly germane to an alliance between birds and dinosaurs (Lowe, 1935, 1944b), the perplexingly apomorphic Apodiformes (Lowe, 1939), and preliminary diagnoses for Cuculiformes (Lowe, 1943), Piciformes (Lowe, 1946) and Coraciiformes (Lowe, 1948). Intermittently during the same period, Lowe also considered possible relationships among ratites and some non-avian Theropoda, e.g. *Struthiomimus* and *Ornitholestes*, although he was hampered by the prevailing confusion between synapomorphy and symplesiomorphy and their respective implications for phylogeny (Lowe, 1928, 1930, 1935, 1942, 1944a, b). René Verheyen (Institut Royale Belgique) authored approximately 35 papers during 1950–60 that centred on problems of avian systematics by means of semi-quantitative methods (e.g. Verheyen, 1956a, b, 1960a, b, 1961). The work by Verheyen, however, was deemed idiosyncratic and largely ignored (Sibley & Ahlquist 1990).

Sibley & Ahlquist (1972, 1987, 1990) chronicled avian systematics since the late 18th century. Raikow (1985a) reviewed the philosophical underpinnings of avian systematics in recent decades, and clarified for the time the fundamental differences among various systematic schools. Avian systematics in the late 20th century has been marked by a trough in morphological phylogenetics (Fautin & Watling, 1999; Jenner, 2004a) and a concomitant peak in molecular systematics. The pessimism expressed by Sibley & Ahlquist (1990) regarding the phylogenetic potential of morphological characters, however, contrasts with surveys of the contributions of both (Patterson, Williams & Humphries, 1993). Bledsoe & Raikow (1990) concluded that considerable congruence existed among reconstructions based on DNA–DNA hybridization, sequence-based analyses, and comparative morphology. In a survey of the history of avian molecular systematics, Meyer & Zardoya (2003) recounted discrepancies between reconstructions of basal lineages based on mtDNA and nuclear genes. As discourse among schools increased, it was evident that the familiar demons of avian systematics haunted both morphological and molecular practices: differential selection and adaptation, convergence, extinction of lineages, challenges of homology and alignment, and heterogeneity of evolutionary rates and branch-lengths.

#### Palaeontological contributions

Fossils essentially are amenable only to morphological study, with the exception of a few, fortunate recoveries of ‘ancient DNA’ (Cooper *et al*., 1992, 2001; Austin, Smith & Thomas, 1997; Cooper, 1997; Sorenson *et al.*, 1999; Paxinos *et al.*, 2002), and typically provide only substandard anatomical material or incomplete specimens. Some of the most intense conflicts among avian systematists stemmed either from a commitment to phenetics or the idiosyncracies of palaeornithological perspectives (e.g. Cracraft, 1979, 1980, 1981; Olson, 1982). Influential for avian systematics was the view that avian fossils are both fragile and correspondingly rare (Olson, 1985), despite compendia indicative of extensive taxonomic diversity (Brodkorb, 1963, 1964, 1967, 1971a, b, 1978). Deficiencies in the fossil record (Olson, 1985) and challenges of homology (e.g. Sereno, 2001), however, did not diminish a reliance on new fossils to resolve the broad outlines of avian evolution (Feduccia, 1980, 1995, 1996).

Palaeontological contributions have been confounded by speculative evolutionary scenarios that extend beyond the underlying systematics (Feduccia, 1973, 1977c, 1995, 1996, 2003). The purported issue of ‘fossil mosaics’ (Eldredge, 1989) – a predictable consequence of heterogeneity in evolutionary rates among characters – further exacerbated the interpretation of evolutionary change (Livezey, 1997a). Martin (1983: 291) concluded that during the 150 years of avian palaeontology, ‘… a major burden for palaeornithologists has been a lack of comparative skeletons of recent birds’, and that the ‘other major problem is the incompleteness of most avian fossils.’ With the latter we agree, but the former is less a problem of availability than the result of under-utilization, a factor worsened by the rush to a molecular era.

#### Ethological and parasitological phylogenetics

Behavioural characters are only infrequently used in formal cladistic analyses (e.g. Hughes, 1996; Lee *et al.*, 1996; Kennedy *et al.*, 1996, 2000; Slikas, 1998; Birdsley, 2002), or precursors thereof (Van Tets, 1965). Complete designs have not been attempted for lack of comparable data for species of interest (Wimberger & de Queiroz, 1996), and some are limited to assessments a posteriori for phylogenetic signal (Winkler & Sheldon, 1993; Lee, Feinstein & Cracraft, 1997; McCracken & Sheldon, 1997). Phylogeneticists have come to consider selected ethological traits – notably displays of courtship – worthy of phylogenetic interpretation (Delacour & Mayr, 1945; Johnsgard, 1961; Archibald, 1976; Paterson, Wallis & Gray, 1995). Patterns of interspecific hybridization have perhaps the longest history of study, notably among Anseriformes (Sibley, 1957; Johnsgard, 1960, 1963; Scherer & Hilsberg, 1982). Eventually, interfertility was recognized to be plesiomorphic and comparatively conservative (Prager & Wilson, 1975), and therefore interspecific hybridization to be uniformative with respect to phylogenetics (Cohen *et al.*, 1997; Braun & Brumfield 1998; Andersson, 1999). Similarly, phylogenetics of ectoparasites has been explored only infrequently in phylogenetic reconstructions of birds (Paterson, Gray & Wallis, 1993; Paterson & Gray, 1996; Page *et al.*, 1998; Johnson *et al.*, 2002; Storer, 2002; Smith, Page & Johnson, 2004; Banks, Palma & Paterson, 2006). Consequently, the two primary sources of phylogenetic signal for birds during the 20th century have been morphological variation and molecular (increasingly DNA sequence) data.

#### Molecular phylogenetics

Following an implicit rejection of DNA hybridization on the grounds of its phenetic nature and woefully incomplete distance matrices, molecular systematics focused on the cladistics of parsimony or increasingly explored the probabilistics of maximum-likelihood and Bayesian methods. Phylogenetic analyses based solely on mitochondrial genes *de jour* (e.g. cyt *b*, 12S) initially were accorded considerable validity (Sraml *et al.*, 1996; Mindell, Sorenson & Dimcheff, 1998; Johnson & Sorenson, 1998, 1999; McCracken *et al.*, 1999), but these works effectively were trumped by those based on entire mitochondrial genomes (Paton, Haddrath & Baker, 2002) or including nuclear genes, with few exceptions (García-Moreno, Sorenson & Mindell, 2003). Similarly, explorations of very limited numbers of genes (Templeton, 1983; Groth & Barrowclough, 1999; Paton *et al.*, 2003; Chubb, 2004a, b; Fain & Houde, 2004) were eclipsed by expanded analyses of nuclear data with diversified taxonomic samples (Hughes & Baker, 1999; Donne-Goussé, Laudet & Hänni, 2002; Sorenson *et al.*, 2003). This progression of analytical refinements and expanded taxonomic representation, despite the continued challenges discovered in each (e.g. Cotton & Page, 2002), is likely to continue and perhaps accelerate with the implementation of studies based on ‘total evidence’ (Huelsenbeck, Bull & Cunningham, 1996; Baker, Yu & DeSalle, 1998; Ballard *et al.*, 1998; Bininda-Emonds, Gittleman & Steel, 2002; Cracraft *et al.*, 2004).

## Current status of avian phylogenetics

‘… the currently accepted arrangement of birds in no way reflects the probable evolutionary history of the class…. The arrangement used here is predicated mainly on the assumptions that birds originated on land rather than in the water, and that highly specialized waterbirds are more derived than less specialized ones…. a consensus has emerged that birds originated, if not in trees, certainly on land. Therefore, we should look for the most primitive taxa among the land birds.’ (Olson, 1985: 83, 84)

‘If one had to summarize the current state of knowledge, the most pessimistic view would see the neoavian tree as a “comb,” with little or no resolution among most traditional families and orders.’ (Cracraft *et al.*, 2004: 475)

‘Perhaps the greatest unsolved problem in avian systematics is the evolutionary relationships among modern higher-level taxa.’ (James, 2005: 1052)

Harrison *et al*. (2004: 974) concluded: ‘It is almost an offense against birds that the deep mammalian tree is virtually resolved … whilst there are still major uncertainties about many aspects of the avian evolutionary tree.’ In support of this sentiment, the authors cited fundamental discordance among phylogenetic inferences for birds based on mitochondrial and nuclear genomes, an assessment at odds with a contemporary review by García-Moreno *et al*. (2003). Discussion of morphological efforts by Harrison *et al*. (2004) was limited to the uncertainties raised by Cracraft (1981, 2001) but verified increasingly by analyses (Cracraft, 1982a, 1986, 1988; Cracraft & Mindell, 1989; Cracraft *et al.*, 2004; Mayr, 2005a). Reconstructions of the higher-order relationships of birds based on morphological characters, in turn, have been depauperate in both characters and taxa and seldom genuinely cladistic (e.g. Cracraft, 1986, 1988, 2001; Cracraft & Clarke, 2001; Mayr & Clarke, 2003).

Regardless of the taxonomic group considered, however, the sobering truth is that the goal of phylogenetics is extremely ambitious and without easy or uniformly reliable means of accomplishment. It is beyond debate that the conceptual framework of morphological cladistics (Hennig, 1966) and ever-increasing computational power has led to significant progress. Nevertheless, it is also clear that many phylogenetic problems have proven resistant to all attempts at solution and seem destined to controversy. Also, phylogenetic endeavours are replete with disagreements in method (both for reconstruction and for evaluation of estimates) and types of evidence considered most reliable. Currently, the tendency is to consider molecular reconstructions as representing the future of avian phylogenetics, and that it is simply a matter of time, perhaps less than a decade, before a global consensus is achieved within the systematic community (Barrowclough, 1992; Livezey & Zusi, 2001; Stanley & Cracraft, 2002).

Deficiencies in taxa or characters typically render comparisons among investigations problematic (Bledsoe & Raikow, 1990), and attempts to reconcile the phylogenetic evidence for Aves substantiate this generality (Cracraft & Mindell, 1989; Mayr, Manegold & Johansson, 2003, 2004a; Dyke & Van Tuinen, 2004; Griffiths *et al.*, 2004). Indicative of disappointing progress in mid- and lower-order avian phylogenetics is the conclusion that basal (higher-order) nodes may be irresolvable or accurate approximations of genuine, explosive radiations (Poe & Chubb, 2004). While demonstrably true of analyses confined to few characters or limited taxonomically (Kumazawa & Nishida, 1995), a single decade of uninspiring inference is insufficient to judge solution to be beyond hope.

The current status of molecular resolution of deepest neornithine nodes, however, serves to underline the likelihood that many genes provide inadequate phylogenetic signal for the problems at hand, a deficiency exacerbated by basal polarities necessarily based on closest extant relatives that are unfortunately comparatively distantly related, e.g. Crocodylia and Testudines (Larhammar & Milner, 1989; Iwabe *et al.*, 2004). The fact that ‘nearest’ outgroup(s) for molecular analysis need to be be extant has had unfortunate implications for rooting, in that for Neornithes these outgroups are comparatively distantly related and may converge on ‘white noise’ as indicators of avian polarities, especially for rapidly evolving mitochondrial data.

**Figure 4:**
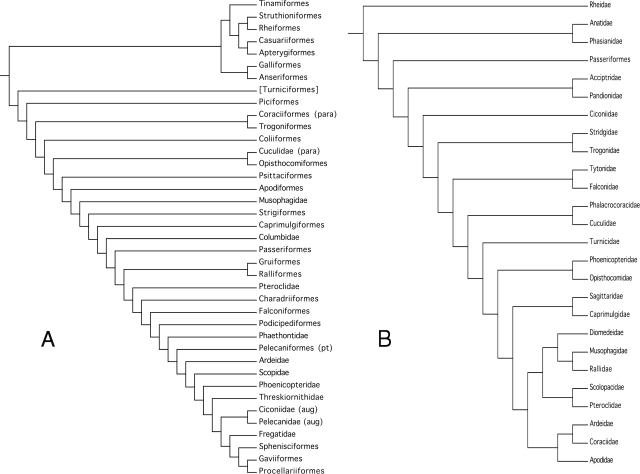
Molecular phylogenetic trees proposed in previous studies (see Fig. 1 for details), IV. A, Sibley & Ahlquist (1990: figs 354–356), simplified to orders, wherein parenthetical ‘para’ indicates paraphyly of sampled members, and ‘aug’ indicates unconventional content; B, Mindell *et al*. (1997).

Like morphological estimates, a number of potential pitfalls (rooting aside) afflict molecular reconstructions, e.g. serial homoplasy by misalignment, distortions related to silent substitutions, unrealistic treatment of ‘gaps’, and unequal evolutionary rates over extended intervals of geological time and among lineages. Furthermore, disagreement persists if not expands regarding methodological preferences – e.g. classes of data employed, protocols for alignment (i.e. diagnosis of serial homology), choice among reconstructive methods, and assessment of resolution and support (Felsenstein, 2004). Until substantial agreement concerning methods is attained and accurate synergism among molecular and morphological methods secured, the field will remain vulnerable to methodological bias and a tolerance for poorly supported hypotheses of phylogeny, in which even the best-supported works disagree significantly (see Figs 4–9).

**Figure 1:**
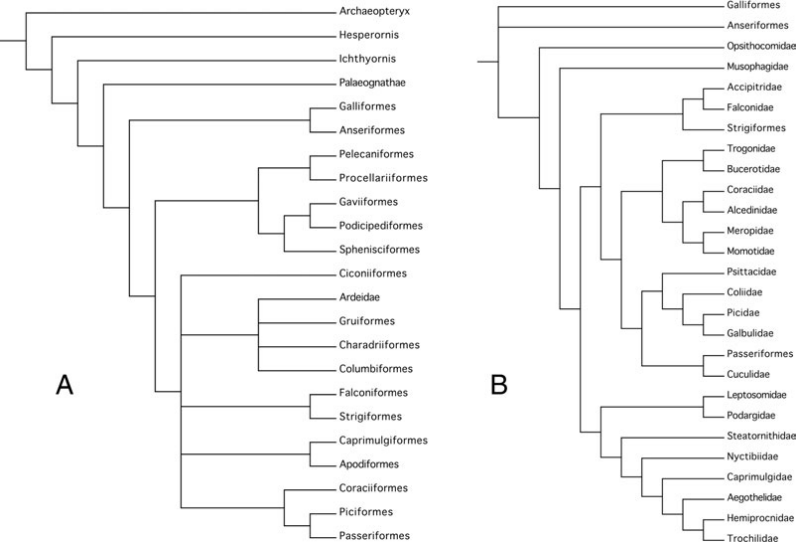
Morphological phylogenetic trees proposed in previous studies, I. A, Cracraft (1988); B, Mayr *et al*. (2003). Some trees were subjected to topologically neutral modifications of taxa to facilitate comparisons (also Figs 2–9). See corresponding papers for analytical methods and topological statistics.

**Figure 2:**
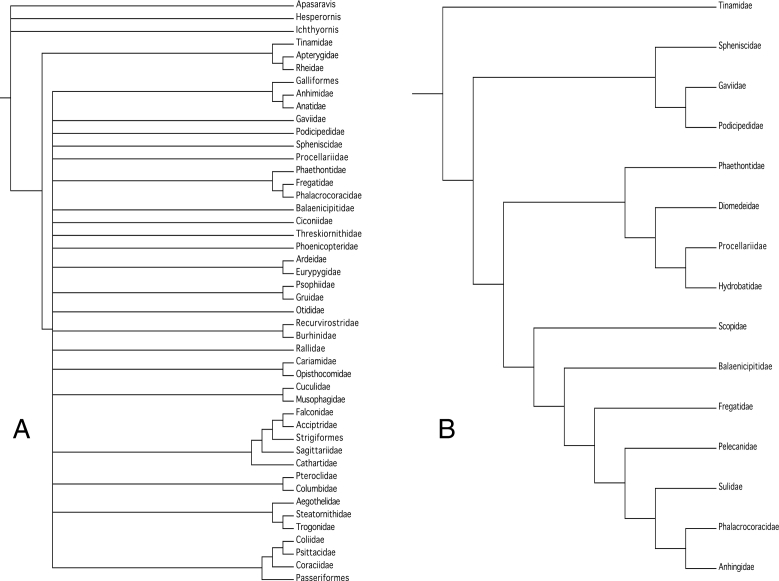
Morphological phylogenetic trees proposed in previous studies (see Fig. 1 for details), II. A, Mayr & Clarke (2003); B, Bourdon *et al*. (2005).

**Figure 5:**
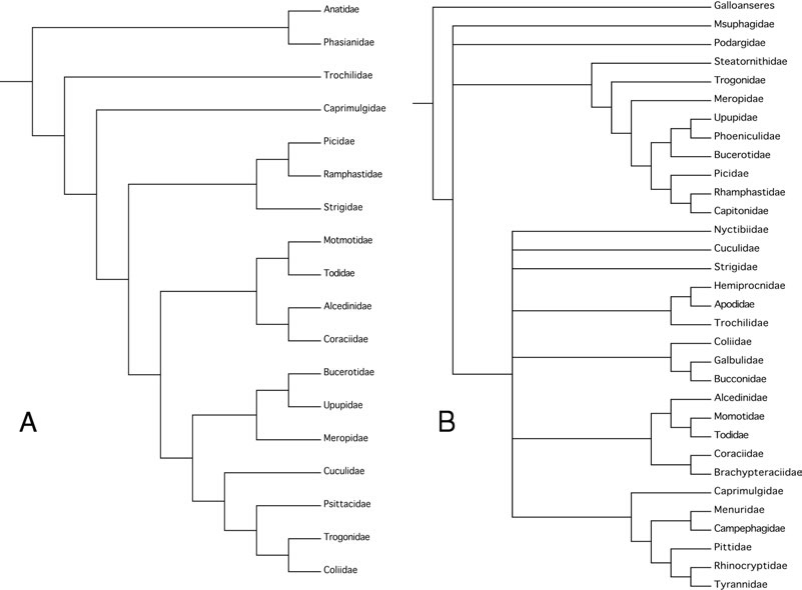
Molecular phylogenetic trees proposed in previous studies (see Fig. 1 for details), V. A, Espinosa de los Monteros (2000); B, Johansson *et al*. (2001).

**Figure 9:**
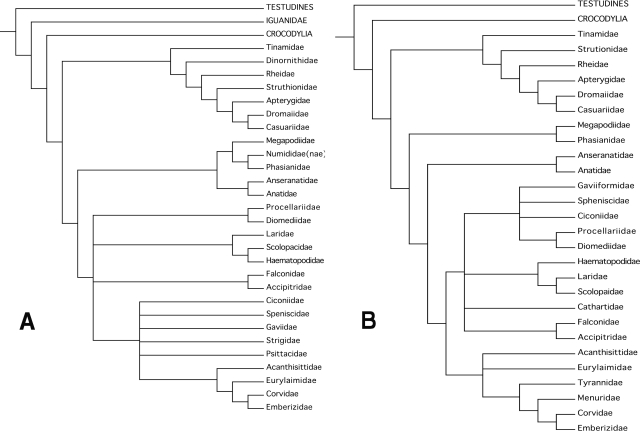
Molecular phylogenetic trees proposed in previous studies (see Fig. 1 for details), IX. A, Pereira & Baker (2006a); B, Slack *et al*. (2006a).

## Goals and objectives

The primary purpose of this paper is to present a morphologically based phylogenetic hypothesis of higher-order relationships of Neornithes. A compendium of characters is provided within the companion work (Livezey & Zusi, 2006), including a bibliographic synthesis, annotations of prior uses of synonymous and related characters, and a compact disc of the data matrix for refinement and augmentation. The secondary objective of this work is to provide a cladistic alternative to the molecular phenetics of Sibley & Ahlquist (1990), at least for non-passeriform families, and to serve as a framework for lower-level studies of included families. An earlier paper on philosophical and methodogical issues (Livezey & Zusi, 2001), despite an explicit disclaimer to the contrary, frequently has been cited as a phylogenetic hypothesis appropriate for comparison with works considered complete by their authors, even regarding positions of individual taxa (e.g. Cracraft *et al.*, 2003). We began the present study with the opinion that the phylogenetic signal encoded within avian anatomy is, with adequate study of both definitive and ontogenetic variation of an adequate sample of modern lineages, more than sufficient for the reconstruction of the higher-order phylogeny of Neornithes. We remain at least as optimistic concerning this goal.

The present phylogenetic hypothesis is intended to serve both as a baseline estimate and ‘scaffold’ for finer-scale reconstructions of terminal clades (i.e. families), as attempts at broad reconstructions of the phylogeny of Neornithes to date have been limited, at the very least, in taxonomic representation (e.g. Slack *et al.*, 2006b) or discredited methods of inference (Sibley & Ahlquist, 1990). We also sought to provide robust nodes supplemental to the few phylogenetic hypothesis currently employed for calibrations of age based on fossils (e.g. Dyke & Van Tuinen, 2004; Pereira & Baker, 2006a) or their surrogates (Van Tuinen, Stidham & Hadly, 2006). Integration of these data with a rich matrix of DNA-sequence data (Cracraft *et al.*, 2004) is planned to explore the power of ‘total evidence’ to recover both higher-level and lower-level avian phylogeny. Perhaps most importantly for the facilitation of future analyses, be these morphological or molecular, is the identification of sister-groups (optimal outgroups) for purposes of rooting analyses of single orders or families. The comparatively sparse representation of taxa in the present analysis reflected logistical limits, but was considered adequate for achieving the stated objectives. Findings herein principally were compared with modern higher-order reconstructions (e.g. Mindell *et al.*, 1997; Mayr & Clarke, 2003; Mayr *et al.*, 2003), the most critical of which are summarized graphically here (Figs 1–9). Works of narrower scope are considered where issues of familial monophyly persist, with emphasis on truly phylogenetic works as opposed to eclectic or phenetic assessments (Raikow, 1985a).

## METHODS

### Included taxa

#### Taxonomic sampling and exemplars

Taxonomic diversity generally represents a much greater logistical burden than diversity of characters in phylogenetic analyses, and challenges imposed by taxa can be exacerbated by unfortunate sampling (Maddison & Maddison, 1992; Graybeal, 1998; Swofford, 2002; Felsenstein, 2004). However, it has been demonstrated that density of taxonomic sampling for the ingroup varies directly with expected accuracy, support and resolution of resultant trees (Lecointre *et al.*, 1993), although the importance of taxonomic density appears to be greatest for sequence data (especially with respect to long-branch attraction). Expectations of resolution and accuracy that are related to richness of morphological characters, unlike for sequence data (Lecointre *et al.*, 1994), have not been subjected to numerical assessment, but logically are significantly related. The importance of monophyly of the groups represented by exemplars prompted the citation, where available, of analyses germane to the monophyly and content of taxonomic families represented here by exemplars.

We sought to maximize richness of characters and represent higher-order taxa within logistic limits that: (i) represented (sub)familial diversity among non-passeriform Neornithes; (ii) provided special insights into interfamilial groups (Livezey, 1997a, 1998a); (iii) were suitably represented by essential specimens; and (iv) included taxa of special interest to avian systematics. Neornithine families were represented by one or more exemplars deemed in most cases to reflect at least a ‘basal’ member (i.e. candidate sister-taxon of other members) of the taxon in question. This method is not without difficulties, as concerns persist regarding the use of exemplars as terminal surrogates for higher-order taxa (Bininda-Emonds, Bryant & Russell, 1998), notably where polymorphism is involved (Yeates, 1995; Simmons, 2001) or monophyly of terminals represented by single exemplars is in question. Also, limitations on specimens of specialized preparations impose critical deficiences on resultant data matrices, an abiding concern of anatomical collections of birds (Livezey & Zusi, 2001; Livezey, 2003a). Relatively strong support for monophyly of most clades alleviated concerns regarding taxonomic sampling, especially given the number of morphological characters employed. However, use of minimal numbers of exemplars justifies caution in the diagnostics given for diverse orders and families herein (Table 2).

**Table 2:** Median branch lengths (*L*) subtending clade identified by taxon among MPTs (values in brackets pertain to polytomies), respective Bremer (support) indices (*B*) for clades (i.e. non-terminal taxa in analysis), and apomorphies both unambiguous (i.e. invariant for set of MPTs) and diagnostic (CI = 1.00) or supportive (0.50 ≤ CI < 1.00) for corresponding taxa (Appendix 1). Characters (numbered) and states (lettered in italics) identify terminal condition of transformation attributed to internode in question; characters, states and provenance of features were described by Livezey & Zusi (2006)

Taxon	*L*	*B*	Diagnostic apomorphies	Supportive apomorphies
Aves	[82]	12	1*b*, 214*b*, 1518*b*, 1912*b*, 1987*d*, 2218*c*	338*b*, 708*b*, 789*a*, 1329*b*, 1312*c*, 1510*a*, 2438*b*, 2446*b*
Ornithurae	124	2	–	1470*b*
Eoaves	139	13	418*c*, 515*b*, 1280*b*, 1297*b*, 1474*b*, 2108*b*, 2212*c*	1333*d*, 1452*b*, 1701*c*, 2227*c*, 2440*c*, 2446*d*
Neornithes	98	11	22*c*	221*b*, 1586*a*, 1687*a*, 1688*a*, 1690*a*, 1819*d*, 2134*b*, 2383*a*
Palaeognathae	106	13	540*b*, 631*b*, 656*b*, 659*b*, 1750*b*, 2029*b*, 2436*c*, 2945*b*	1330*b*, 1523*a*, 2028*b*, 2133*c*
Crypturi*	102	–	924*b*	1361*b*, 1453*b*, 1635*b*, 1645*b*, 1844*b*, 2351*b*, 2497*a*
Ratitae†	241	50	129*a*, 250*b*, 474*a*, 523*b*, 547*b*, 555*b*, 765*b*, 767*a*, 901*b*, 923*b*, 926*b*, 958*b*, 1046*b*, 1051*b*, 1263*b*, 1341*b*, 1537*b*, 1554*b*, 1564*c*, 1861*a*, 2022*b*, 2045*b*, 2165*b*, 2184*b*, 2512*b*, 2721*b*, 2757*c*, 2794*b*, 2798*b*, 2867*b*	476*b*, 506*b*, 600*a*, 927*b*, 1008*e*, 1019*b*, 1041*a*, 1053*b*, 1098*f*, 1122*b*, 1258*a*, 1333*e*, 1336*c*, 1337*c*, 1346*c*, 1346*b*, 1353*a*, 1364*c*, 1371*c*, 1450*a*, 1497*b*, 1509*b*, 1548*b*, 1694*b*, 1707*a*, 1709*a*, 1744*a*, 1747*c*, 1756*d*, 1766*b*, 1773*b*, 1924*b*, 1998*a*, 2015*d*, 2167*a*, 2479*b*, 2522*b*, 2547*b*, 2568*b*, 2717*b*, 2769*b*, 2808*b*, 2811*a*, 2821*a*, 2868*a*
Casuariimorphae	88	31	352*b*, 1120*b*, 1121*b*, 1167*b*, 1170*b*, 1390*b*, 2306*b*	413*c*, 930*b*, 952*b*, 962*b*, 1156*b*, 1789*b*, 1844*b*, 1355*b*, 2667*b*, 2949*b*
Struthionimorphae	100	44	107*b*, 1065*b*, 1154*b*, 1169*b*, 1784*b*, 1896*b*, 2002*b*, 2302*b*, 2326*b*, 2398*c*, 2795*b*, 2824*b*	1371*c*, 1551*b*, 1568*b*, 1756*c*
Neognathae	142	52	213*b*, 523*c*, 579*b*, 601*b*, 1096*b*, 1106*b*, 1487*c*, 1809*b*, 1953*b*, 2068*b*, 2108*c*, 2209*b*, 2216*b*, 2217*b*	2*e*, 4*c*, 109*b*, 112*c*, 468*b*, 583*c*, 600*d*, 731*d*, 1497*d*, 1633*c*, 1789*b*, 2294*b*
Galloanserimorphae	82	18	117*b*, 513*b*, 546*b*, 601*c*, 698*b*, 723*b*, 2073*b*, 2855*b*, 2915*b*	–
Galliformes	137	86	542*b*, 625*b*, 1077*b*, 1109*b*, 1247*b*, 1257*b*, 1362*b*, 1657*b*, 1906*b*, 2693*b*, 2859*b*, 2907*b*	109*d*, 378*b*, 524*b*, 600*c*, 750*b*, 1175*c*, 1196*b*, 1330*b*, 1792*c*, 2146*a*
Anseriformes	97	41	95*b*, 278*b*, 2052*b*, 2073*c*, 2148*b*, 2454*b*, 2724*b*, 2747*b*, 2913*b*	422*b*, 1333*b*, 2497*a*
Neoaves	81	18	1280*c*, 2502*b*, 2586*b*, 2893*b*, 2895*b*, 2896*b*, 2900*d*	480*c*, 517*c*, 600*e*, 1721*b*
Natatores	51	1	–	–
Pygopodo-tubinares	104	43	195*c*, 1432*b*, 2076*b*, 2413*a*	–
Gaviomorphae	95	52	534*c*, 538*b*, 748*b*, 2117*b*, 2147*b*, 2249*b*, 2256*b*	927*b*, 946*c*, 1514*b*, 1766*b*, 1924*b*, 2077*b*, 2089*d*, 2287*b*, 2362*b*, 2402*a*
Gaviiformes	125	–	457*b*, 1407*b*, 1532*b*, 1893*b*, 2002*c*, 2320*b*, 2331*b*, 2411*b*, 2644*b*, 2694*b*, 2882*b*, 2886*b*	1193*b*, 1820*b*, 2133*b*, 2322*b*, 2349*c*
Podicipediformes	121	–	823*b*, 2304*b*, 2435*c*, 2642*b*, 2658*b*, 2771*b*, 2784*b*	164*b*, 1008*d*, 1657*b*, 2054*d*, 2056*b*, 2429*b*
Procellariimorphae	66	12	196*b*, 722*b*, 1347*b*, 2404*b*, 2630*b*, 2744*b*, 2933*b*	2225*c*, 2356*b*, 2729*d*
Sphenisciformes	225	–	448*b*, 534*b*, 910*b*, 933*b*, 1422*b*, 1424*b*, 1495*b*, 1517*b*, 1530*b*, 1541*b*, 1542*b*, 1544*b*, 1556*b*, 1571*b*, 1736*b*, 1749*c*, 1751*b*, 2293*c*, 2366*b*, 2528*b*, 2543*b*, 2546*b*, 2720*b*, 2790*b*, 2791*b*, 2870*b*	385*e*, 994*b*, 1302*b*, 1353*c*, 1364*d*, 1443*b*, 1451*c*, 1516*c*, 1580*c*, 1581*b*, 1694*b*, 1707*c*, 1733*a*, 1755*c*, 1756*c*, 1820*b*, 2004*b*, 2133*b*, 2446*c*, 2522*b*, 2563*b*, 2584*b*, 2588*b*, 2610*c*, 2810*c*, 2812*d*, 2868*a*
Procellariiformes	33	64	1305*b*, 1306*b*, 2847*b*	2819*a*
Stegano-grallatores	61	8	–	1138*b*
Pelecanimorphae	102	24	720*b*, 1241*b*, 2107*b*, 2751*b*	24*a*, 372*b*, 1144*b*, 1540*d*, 1999*b*, 2089*b*, 2729*b*
Balaenicipitiformes	158	–	257*b*, 288*b*, 293*b*, 433*b*	153*b*, 286*b*, 304*b*, 566*b*, 740*b*, 762*b*, 769*c*, 780*c*, 1309*b*, 1514*b*, 2346*b*
Pelecaniformes	111	16	335*b*, 889*b*, 1832*b*, 2573*b*	35*b*, 48*b*, 147*b*, 946*d*, 2181*b*, 2351*b*, 2388*b*, 2406*b*, 2548*b*
Ciconiimorphae	55	9	1543*b*	1138*c*, 2179*b*, 2830*b*
Ciconiiformes	58	9	–	2420*b*
Ardeiformes	106	66	116*b*, 175*b*, 754*b*, 2097*b*, 2391*b*, 2396*b*, 2458*b*, 2800*b*, 2834*b*, 2836*b*, 2851*b*	35*b*, 147*b*, 529*b*, 535*b*, 831*b*, 1238*c*, 2028*b*, 2330*b*, 2388*b*
Terrestrornithes‡	42	1	–	–
Charadriimorphae	45	10	–	2575*b*
Gruiformes	40	7	2111*b*	2146*a*, 2197*b*
Charadriiformes	30	25	–	2462*b*
Dendrornithes§	54	4	–	–
Falconimorphae	62	6	2916*b*	2431*b*
Falconiformes	83	19	120*d*, 1857*b*, 1938*b*, 2343*b*, 2428*b*	1153*c*, 2133*b*, 2854*b*, 2899*b*
Strigiformes	172	122	13*b*, 154*b*, 174*b*, 193*b*, 548*b*, 1549*b*, 1714*b*, 2072*b*, 2200*a*, 2286*b*, 2300*b*	128*c*, 249*b*, 1540*c*, 1569*c*, 1779*b*, 1822*b*, 2407*b*, 2412*b*, 2583*c*, 2602*a*, 2694*b*, 2710*d*, 2743*b*
Anomalogonates¶	56	6	–	–
Cuculimorphae	185	33	1572*b*	1007*b*, 1336*b*, 1614*b*, 1651*d*, 1658*c*, 1866*b*, 2634*c*, 2849*a*
Opisthocomiformes	131	–	959*b*, 1064*b*, 2857*b*	385*e*, 566*b*, 850*e*, 1063*b*, 1140*b*, 1329*d*, 1651*e*, 1658*d*, 2091*b*, 2510*b*, 2575*b*, 2710*d*, 2845*b*
Cuculi	85	42	937*b*, 2034*b*, 2046*b*, 2061*b*, 2200*c*, 2334*e*	1122*b*, 2498*b*
Psittacimorphae	52	1	–	–
Psittaciformes	177	130	118*b*, 246*b*, 354*b*, 410*b*, 429*b*, 593*b*, 605*c*, 650*b*, 679*b*, 703*b*, 761*b*, 1060*b*, 1210*b*, 2334*d*, 2405*d*, 2490*b*, 2703*b*, 2712*b*, 2832*b*, 2876*c*, 2878*b*, 2884*b*	772*b*, 2028*b*, 2127*b*, 2133*d*, 2203*b*, 2491*b*, 2498*b*, 2673*b*, 2710*c*, 2849*e*, 2854*c*, 2935*b*, 2941*b*
Columbiformes	106	62	1723*b*, 2119*b*, 2557*b*, 2846*b*	351*b*, 1175*b*, 1307*b*, 1356*b*, 1369*b*, 1417*c*, 2036*b*, 2575*b*, 2710*d*, 2722*b*
Incessores**	101	2	–	–
Cypselomorphae	70	11	2786*b*	1365*b*, 2903*b*
Apodiformes	97	49	1125*b*, 1375*b*, 1416*b*, 1455*b*, 1465*b*, 1466*b*, 2449*c*, 2661*b*	38*b*, 1346*b*, 2466*a*, 2549*c*, 2674*b*
Caprimulgiformes	77	9	280*b*, 1271*b*, 1979*b*, 2198*c*, 2921*b*	128*c*, 230*b*, 249*c*, 450*b*, 1999*b*, 2583*c*, 2903*d*, 2933*b*
Trogones††	38	2	–	–
Trogonomorphae	43	2	1919*b*	–
Trogoniformes	83	58	450*b*, 935*b*, 1720*b*, 2333*b*	244*b*, 531*b*, 2100*b*, 2593*c*, 2613*b*
Coliiformes	108	–	512*b*, 1032*b*, 2338*b*, 2367*b*	398*b*, 2036*b*, 2127*b*, 2673*b*, 2702*b*
Passerimorphae‡‡	[62]	13	1807*b*, 2590*b*	718*b*, 1453*b*
Coraciiformes	51	6	2360*b*	2723*b*
Piciformes	58	9	2334*g*, 2335*b*	1300*b*, 1709*c*, 1981*b*, 2498*d*
Passeriformes	96	55	159*b*, 1228*b*, 1463*b*, 1895*c*, 2669*b*, 2687*b*, 2874*b*, 2877*b*, 2890*b*, 2891*b*	1127*b*, 1540*e*, 1559*b*, 2789*c*

* Redundant with taxon of next-lower rank – Dromaeomorphae – by hierarchical classification, and equivalent to apomorphies of terminal taxon Tinamiformes. Other example are (i) Subcohort Galloanseres comprising solely the Superorder Galloanserimorphae; and (ii) Superorder Casuariimorphae comprising solely the Order Casuariiformes.

† Pertains to estimate exclusive of two extinct members (Dinornithiformes, Aepyornithiformes); see Methods.

‡ Redundant with taxon of next-lower rank – Telmatorae – and therefore latter was not tabulated.

§ Redundant with taxon of next-higher rank – Raptores – and therefore latter was not tabulated.

¶ Redundant with taxon of next-lower rank – Coccygae – and therefore latter was not tabulated.

Musophagidae.

** Redundant with taxon of next-lower rank – Cypselomorphae – and therefore latter was not tabulated.

†† Redundant with taxon of next-lower rank – Trogonomorphae – and therefore latter was not tabulated.

‡‡ Redundant with taxon of next-higher rank – Pico-clamatores – and therefore latter was not tabulated.

Crocodylia and non-avian theropod Dinosauria served as ‘ultra-deep’ and primary outgroups, respectively, to root Neornithes (Maddison, Donoghue & Maddison, 1984; Janke & Arnason, 1997), but the inclusion of most published characters in placing these taxa (Benton & Clark, 1988; Evans, 1988; Benton, 1999; Cao *et al.*, 2000; Brochu, 2001; Brochu & Norell, 2001) chronicled the acquisition of avian characters during the Mesozoic (Carroll, 1997). The recent extension of avian roots, both by newly discovered avialian taxa and confirmation of early roots among non-avian theropods, circumvented difficulties of establishing basal polarities for Neornithes based on inadequate diversity of Mesozoic relatives or (for narrower reconstructions) or dubious reliance on the problematic Palaeognathae, notably caused by the complex of apomorphy and plesiomorphy of ratites relative to the Tinaniformes (Bertelli, Giannini & Goloboff, 2002). These outgroups optimized rooting by the hierarchy of information afforded by multiple (nested) outgroups (Barriel & Tassy, 1998; Lyons-Weiler, Hoelzer & Tausch, 1998) and avoided the analytical problems implicit with hypothetical ancestors (Bryant, 1997, 2001).

Four comparatively distant outgroups were sampled for estimating deep polarities – non-Archosauromorpha (informative states of comparable characters at the approximate origin of the archosaurian clade), Crocodylomorpha (i.e. non-dinosaurian Archosauria), Ornithischia (i.e. non-saurischian Dinosauria) and Sauropodomorpha (modalities of non-theropod Saurischia). Among non-avian Theropoda, *Herrerasaurus* served as the most informative of the generic outgroups (Sereno, 1994; Sereno & Novas, 1994). Groupings among outgroups (i.e. among non-avian taxa) were of only secondary interest, however, whereas establishment of a realiable root for the Neornithes was the principal priority.

Indeterminate and redundant contributions of some outgroup taxa with respect to the primary objective of this analysis, as well as excessive proportions of missing data recognized upon completion of the data matrix, prompted limited pruning and merging of taxa (primarily outgroups) for analysis: (a) taxa pruned –*Euparkeria*, *Syntarsus*, *Eoraptor*, *Saurornitholestes*, ‘Caenognathidae’, *Microvenator*, *Citipati*, *Chironestes*, *Ornitholestes*, *Segnosaurus*, *Avimimus*, *Sinornithosaurus*, *Microraptor*, *Erlicosaurus*, *Shuvuuia*, *Jehelornis*, *Gobipteryx*, *Patagopteryx*; Diatrymiformes, Dromornithiformes (Rich, 1979, 1980; Murray & Megirian, 1998; Murray & Vickers-Rich, 2004), *Sylviornis* (Poplin & Mourer-Chauviré, 1985; Mourer-Chauviré & Balouet, 2005); (b) taxa merged: {*Allosaurus*, *Sinraptor*}∼ Allosauroidea; {*Tyrannosaurus*, *Albertosaurus*}∼ Tyrannosauridae; {*Sinovenator*, *Sinornithoides*, *Troodon*}∼Troodontidae; {‘Enantiornithidae’, *Iberomesornis*, *Cathayornis*, *Concornis*, *Neuquenornis*, *Eoalulavis*, *Protopteryx*}∼Enantiornithes; {*Mononykus*, *Patagonykus*, *Alvarezsaurus*}∼Alvarezsauroidea.

Two subfossil taxa – Aepyornithiformes and Dinornithiformes – for which character states were only marginally recovered, were excluded for the primary global search, and provisionally placed by means of two different protocols. This measure was taken because simple analysis of these imperfectly known, broadly similar, large ratites led to an apparently artefactual couplet –‘long-branch’ distortions exacerbated by missing data (Wiens, 2005) – as sister-group of other ratites exclusive of Apterygidae. First, each was analysed in the absence of the other in a global, unconstrained analysis. Second, each was separately placed by means of heuristic searches in which the primary tree was used as a backbone constraint. The Dinornithiformes were scored as two families (Dinornithidae and Emeidae) as approximated by Cracraft (1976a, b) and Worthy and Holdaway (2002) during character analyses, but analysed as a single, merged taxon in light of their virtually identical scores. Accordingly, the ‘trimmed-merged’ data matrix provided in digital form by Livezey & Zusi (2006) comprised 150 ingroup taxa and 35 outgroups.

## Phylogenetic analysis

### General philosophy

Most standard methodological issues were detailed in the foregoing companion work (Livezey & Zusi, 2006), including the analytical perspectives that serve to justify the delimitation of characters and states, ordering of states, and related options requisite to preparation of characters for analysis. Noteworthy is a principal reliance on the literature for many characters of non-avian Theropoda. In the present installment, the foregoing characters were subjected to phylogenetic analysis *sensu* morphological cladistics (Kluge & Wolf, 1993) coupled with the criterion of parsimony of character evolution as implied by the resultant phylogenetic hypothesis (Eldredge & Cracraft, 1980; Wiley, 1981; Brady, 1982; Farris, 1982; Felsenstein, 1983, 2004; Semple & Steel, 2003). In light of the practical and theoretical implications of adopting the parsimony criterion (Felsenstein, 1983, 2004), alternative methods were not practical for this analysis because of missing data (Felsenstein, 1979; Kluge, 1997a, b, 1999) – e.g. optimizations of morphological characters on branching models under selected models of stochastic change (Huelsenbeck, Nielsen & Bollback, 2003) or maximum-likelihood analysis (Lewis, 2001). Global parsimony – i.e. minimal total for character-state changes required by final tree (i.e. ‘shortness’) – served as the criterion of optimality for trees recovered through searches (Sober, 1982, 2005). The data matrix was not revised iteratively conditional on outcomes of analysis, nor was ordering of characters conditional on such runs. Instead, the entire data matrix summarized herein was completed prior to the analytical phase, thereby maintaining a partition between (i) definition of characters and states, coding of taxa, and issues of weighting and ordering, and (ii) phylogenetic analysis.

### Characters and states

Unfortunate logistic limitations, not oversight or philosophical considerations, prevented the inclusion of character descriptions with the present analytical work. Although a reflection of our unexpected success in defining 2954 morphological characters relevant to the project (Livezey & Zusi, 2006), it precluded the familiar juxtaposition of descriptive material with analytical inferences. We anticipate that this inconvenience will be ameliorated by the coordinated publication of the descriptive atlas of characters and digitally recorded data matrix (Livezey & Zusi, 2006), to be made available virtually at cost. We strongly recommend that those interested in the present work procure a copy of its sister publication, as it is through examination of both that meaningful improvements will be made.

Where mutually exclusive states of a single character were diagnosable (Stevens, 2000), a single multistate character was defined (Mishler, 1994, 2005). Where two or more included states are observed for a single taxon, a coding of polymorphism was used and analysed specifically as given (i.e. not as uncertainty). The expanse of time reflected by the scale of the analysis also is expected to be associated with the number of multistate characters recognized (Lipscomb, 1992; Steel & Penny, 2005), i.e. scale of time and taxonomic divergences may be expected to be related directly to scale in evolution of form (Grant & Kluge, 2004). Multistate characters encode features manifesting comparatively great evolutionary change and may include greater potential phylogenetic signal, and states thereof will be optimized at multiple internodes (Simmons, Reeves & Davis, 2004). Unless otherwise indicated, characters were analysed as unordered.

Ordering can impose significant constraints on solution sets (Hauser & Presch, 1991; Forey & Kitching, 2000), and was used only where determined to be defensibly realistic, e.g. naturally ordinated (Livezey & Zusi, 2006). For example, multistate characters of forms ‘small, medium, large’, ‘absent, miniscule, prominent’, courses of passage of types ‘depressio, sulcus, arcus, tuba’, and junctura of types ‘articulatio, sutura, synostosis’ were considered naturally ordered, counter-evidence lacking. Fundamentally, ordering of states within a character was fundamental to definition and differentiation of characters, basic to the delimitation of states, and represented an extension of parsimony by inclusion of information on linear likelihoods in coding schemes. Such reasoning precluded meaningful use of arbitrary analytical variants such as treating all characters or partitions thereof as unordered. Hypotheses of transformation were sufficiently simple to obviate reliance on step-matrices (Ree & Donoghue, 1998), linear ordering being the sole condition imposed. Different numbers of states among characters can impose different levels of influence simply by different numbers of state changes among characters (James, 2004), but we considered such differential influence to be realistic and justified as it encoded diverse richness of evolutionary change instead of arbitrarily imposing uniformity on contributions of signal. Therefore, no attempt was made to counter-weight multistate characters. Moreover, no method of explicit weighting – a priori (Neff, 1986; Wheeler, 1986; Sharkey, 1989) or successive (Farris, 1969) – was employed in this analysis, although some perceive weighting effects to be implicit by other means (Haszprunar, 1998).

In this work, characters qualifying as autapomorphies at this analytical scale (i.e. apomorphic state limited to single included terminal taxon) were included in all analyses because most served as synapomorphies of the higher-order groups represented by respective exemplars, and many were included in previous publications as diagnostic of the clades represented by exemplars. In addition, such characters are intended to serve others performing lower-level analyses subsequently using some or all of the present matrix. Although autapomorphies did not serve to group taxa at this scale, the limited number detected here were retained because our interests not only included delimitation of clades but also were intended to provide a reasonable representation of evolutionary rates both among internodes and among terminal branches, of interest in many studies of evolutionary rates (e.g. Omland, 1997a, b). Also, autapomorphic differences (deriving from both unique changes or homoplasy) are critical to long-standing issues of perceived (phenetic) distinction and evolutionary divergence among taxa of debated relationships. Furthermore, such characters do not bias support indices such as bootstrapping (Harshman, 1994a), and by definition do not influence topologies. Also, a small minority of characters manifesting two or more states in original codings (included in the matrix to permit alternative taxonomic treatments) were rendered invariant by merging and pruning of taxa as detailed herein; this treatment was considered simpler than outright manipulation of the matrix analysed. The primary parameter of logistical concern where parsimony is the criterion of optimization is the number of taxa (Kim, 1998), a dimension that in the present work was favourably countered by number of characters.

Included characters manifested a range of homoplasy (Sanderson & Donoghue, 1989). However, the number of morphological characters employed here exceeded the domain for which meaningful comparison with other works is feasible (Swofford, 1991; Sanderson & Donoghue, 1989, 1996) and evaluation of a suite of related issues – e.g. rates of evolution, notions of relative ‘reliability’ of different data types, patterns of homoplasy (Faith, 1989; Sanderson, 1991), and Markovian informativeness (Shpak & Churchill, 2000) – was not logistically feasible.

### Search for optimal solution

The character matrix was constructed using MACCLADE (Maddison & Maddison, 1992; Prendini, 2003), and analyses were performed on a Macintosh G5 2.5-GHz dual-processor computer. Primary phylogenetic analyses were performed using PAUP* version 4.0b10 (Swofford, 2002). Given the size of the data set and the corresponding universe of possible trees delimited (Felsenstein, 1978), we undertook a thorough exploration of the tree space to circumscribe the optimal solution set, i.e. the set of maximally parsimonious trees (MPTs), summarized graphically by a strict consensus tree of this set.

The set of MPT(s) (min [total length] = 19 553) recovered during heuristic searches in PAUP (MULPARS, TBR, random-addition of taxa, 10 000 random starting trees, MAXTREES = 20 000) was confirmed by a full ratchet-analysis (Goloboff, 1999; Nixon, 1999; Müller, 2004, 2005), including five random-addition cycles of 200 ratchet iterations each; the ratchet analyses, employed to escape local suboptima, recovered 97 trees across 1000 topological islands. Choice of optimizations (DELTRAN vs. ACCTRAN) was ineffectual, and neither DOLLO nor IRREVERSIBLE options were used. Of particular relevance to this comparatively large analysis were recent discussions of: (i) efficient means for finding solutions for large data sets (Maddison, 1991; Page, 1993; Rice, Donoghue & Olmstead, 1997; Quicke, Taylor & Provis, 2001; Salter, 2001), (ii) effects of missing data (Wilkinson, 1995, 2003; Wiens, 2003) and (iii) analytical relevance of branch lengths (Maddison, 1993; Lyons-Weiler & Hoelzer, 1997; Farris, Källersjö & De Laet, 2001; Norell & Wheeler, 2003; Wilkinson, LaPointe & Gower, 2003).

Summary statistics used here were: total length, L; consistency index, CI (Klassen, Mooi & Kicke, 1991; Kim 1996; Källersjö, Albert & Farris, 1999); retention index, RI (Farris, 1989); rescaled consistency index, RC; and skewness index (*g*_1_) based on 10^5^ topologies randomly generated from the same data matrix (Huelsenbeck, 1991; Källersjö*et al.*, 1992). Despite its popularity, the CI is negatively correlated with number of both taxa and characters analysed (Archie, 1989; Sanderson & Donoghue, 1989), making meaningful comparisons of indices across scales of analysis and classes of characters is difficult. Characters manifesting homoplasy can impose structural resolution and thereby result in smaller solution sets of MPTs (Källersjö, Albert & Farris, 1999). The set of equally parsimonious topologies (i.e. solutions differing only in optimization of characters on branches of trees of identical topology or solutions differing in branching structure but of equal length) were summarized using a strict consensus tree. Summary statistics for strict consensus trees were component information, Nelson–Platnick term and total information, and Mickevich consensus information.

Support for individual clades was measured by two statistics (Mort *et al.*, 2000; Wilkinson *et al.*, 2003): (i) percentages of 10 000 bootstrapped replicates in which the node was conserved (Felsenstein, 1985; Sanderson, 1995), indices considered informative even if assumptions concerning precision and absence of bias are unrealistic (Felsenstein & Kishino, 1993; Hillis & Bull, 1993); and (ii) Bremer (support) indices, the estimated minimal number of additional steps required wherein the given node, by inverse constraint, is not conserved (Bremer, 1994, 1997). The latter were estimated using PRAP (Müller, 2004, 2005), metrics similar to the PC-compatible algorithms of Goloboff (1999) and Nixon (1999). Ratchet methods were used in order to find the minimum Bremer index by avoidance of entrapment in local optima (Maddison, 1991). For the Bremer (support) indices, 20 ratchet replicates per node were used (Müller 2004, 2005). The popular alternative protocol, TREEROT, was not employed because its primary asset –‘partitioned’ support indices – were not a priority here and (most importantly) the algorithm lacks the ratchet (Sorenson, 1999).

### Comparisons with other trees

Tests of alternative hypotheses proposed by other authors were equivalent to local penalties, i.e. minimal differences in total length imposed by the alternative hypothesis, while other aspects of the MPT (exclusive of pruning of taxa essential for comparability) were conserved (Kluge, 1997a, b). These estimates were made by simple transfer of branch(es) within the consensus cladogram using MACCLADE (Maddison & Maddison, 1992), which holds other topological groupings constant. This procedure differs from searches constrained only to the grouping of interest, typically performed using ancillary searches under inverse constraints, as in protocols for estimation of Bremer (support) indices.

## Critical concepts and terminology

‘**It is possible**[50% likelihood] that – 1) a distant relationship exists between *Apteryx* and a tinamou-galliform assemblage; … (5) the diurnal birds of prey may be allied to the owls through the Falconidae … **It is improbable**[formerly widely believed, since discredited] that – 1) a close relationship exists between *Rhea* and the tinamous; … (3) *Pandion* deserves familial status in the Falconiformes …’ (Sibley & Ahlquist, 1972: 241), emphasis added.

‘… the mousebirds, or colies, [*i*] have no close living relatives, … [*ii*] they are the only survivors of an ancient divergence … Their [*iii*] closest living relatives are probably …’ (Sibley & Ahlquist, 1990: 363)

Before considering specific findings in the present study, a clarification of critical terms is essential. The first of the foregoing quotes comprises four statements of perceived probability that either make no objective sense or are self-contradictory by conventional standards. Also, the second quote contains three conclusions (i–iii) for a single group based on a single data set that are: either mutually contradictory (i and iii), or of undetermined meaning (ii vs. either i or iii). In cladistic terms, ‘most closely’ implies ‘closely’ in that hierarchy defines relative relationships. Sister-groups are by definition the ‘most closely related’ of any taxa compared. For example, in cladistic terms, an assumption of monophyly of life on earth implies that every taxon has a close relative and/or closest relative, regardless of extinctions. In other words, degree of relatedness is relative: all lineages have a closest relative and therefore a close relative. Sister-groups need not meet some standard of similarity or absolute antiquity of divergence to qualify. However, under an expectation of at least a limited correlation between evolutionary change in morphology with time – neither ‘clock-like’ nor wildly heterogeneous and completely disassociated – sister-taxa can be expected to share degrees of similarity broadly related to time since divergence, such that recency of divergence between sister-taxa tends to be associated with similarity, and antiquity of such divergence to be associated with dissimilarity.

## RESULTS

### Minimal-length trees or MPTs

The search for MPTs recovered 97 trees of minimal length (19 553 steps) under standard ordering of multistate characters and rooting by outgroup taxa as given (see Methods). This solution set (2.04 × 10^11^ rearrangements assessed) had the following summary statistics: CI = 0.2432; RC = 0.1664; RI = 0.6842; and skewness, *g*_1_|10^5^ = −0.4258.

**Figure 10:**
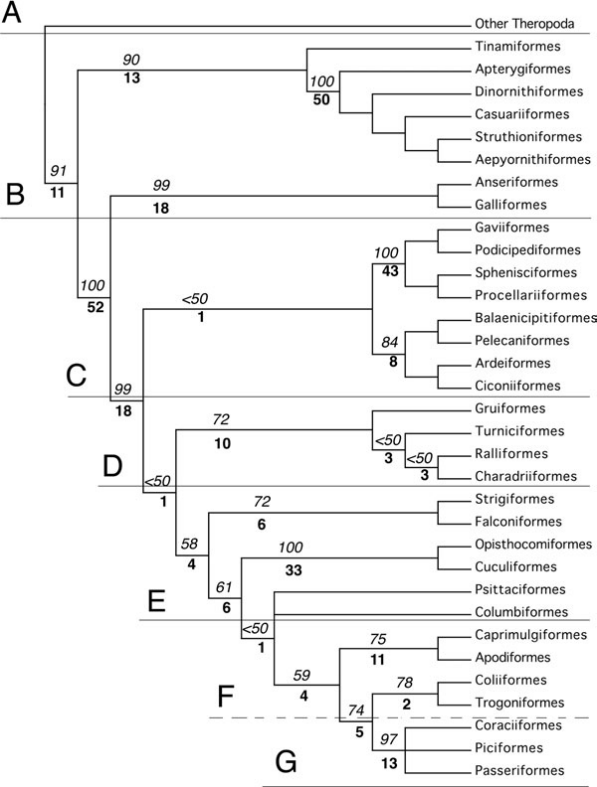
Ordinal-level strict consensus tree for orders of Neornithes based on 2954 morphological characters, indicating delimitations of segments detailed in Figures 12–18.

**Figure 12:**
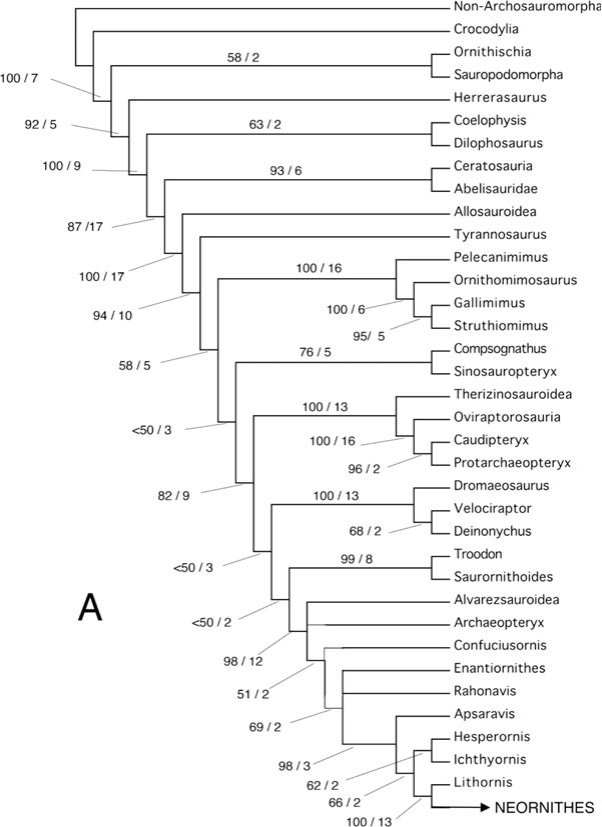
Detailed segments of strict consensus tree of all MPTs recovered in present study. Part A. Outgroup (non-neornithine) taxa. Nodes are labelled by percentages of bootstrapped replicates in which node was retained (numerator), and below by Bremer support indices (denominator).

**Figure 18:**
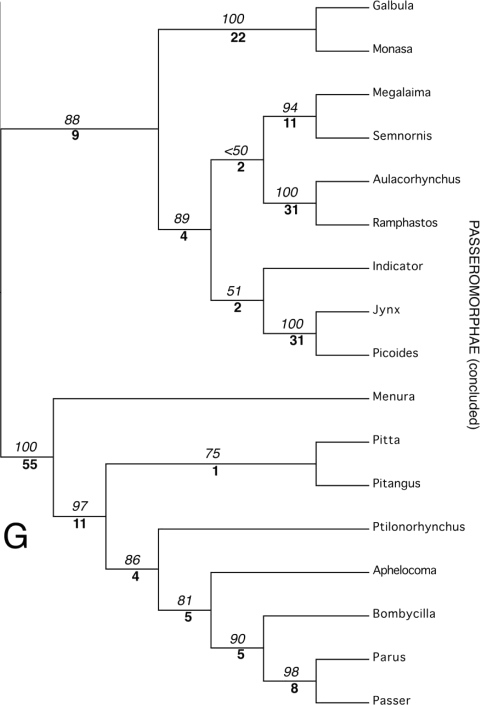
Detailed segment of strict consensus tree of all MPTs recovered in present study. Part G. Neornithes: Piciformes, and Passeriformes. Nodes are labelled above by percentages of bootstrapped replicates in which node was retained (italics), and below by Bremer support indices (bold type).

**Figure 11:**
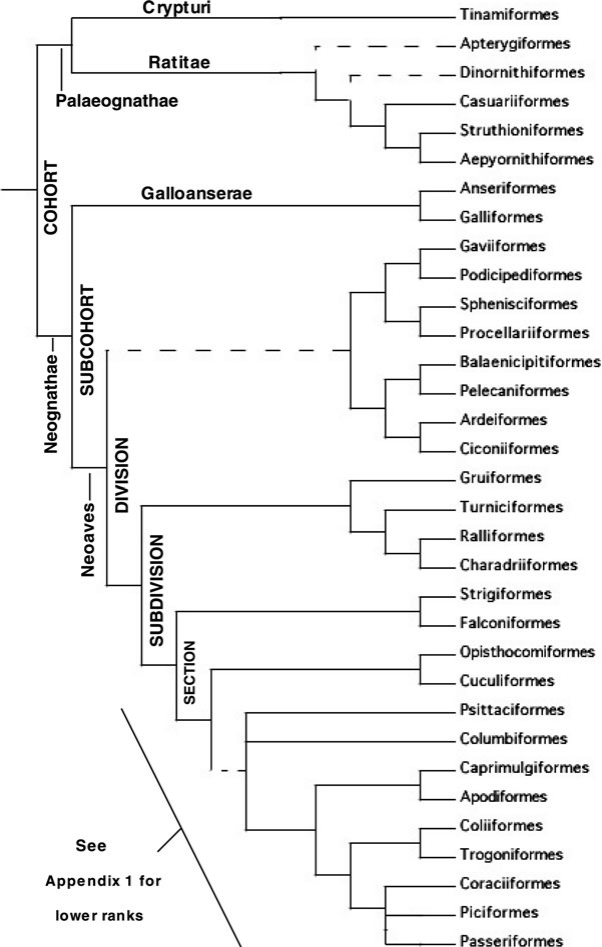
Simplified summary tree for uppermost, supraordinal ranks of avian classification. Dashed internodes correspond to marginally supported clades. For complete classification, see Appendix 1.

A strict consensus tree of the MPTs (Figs 10–18) was completely resolved for the Neornithes with the exception of six polytomies (mostly trichotomies, some nested, discussed below), uncertainties sufficiently limited so as to obviate a majority-rule consensus tree for the primary solutions set, or to delimit ambiguity where one or more ‘rogue taxa’ may be influential (Sumrall, Brochu & Merck, 2001). The strict consensus tree for the 97 MPTs shared the following summary statistics: (i) component information, 173; (ii) Nelson–Platnick term information, 4367; (iii) Nelson–Platnick total information, 4540; and (iv) Mickevich consensus information, 0.168.

### Outgroup taxa: Mesozoic roots of Aves

#### Non-Neornithine Aves

In light of the growing consensus regarding fossil lineages of the Mesozoic and widely employed characters thereof, broad agreement between our findings and those of others treating pre-neornithine birds was not unexpected. Relationships among outgroup taxa in this analysis generally were consistent with recent analyses (Martin, 1983; Witmer, 1991; Holtz, 1998; Padian & Chiappe, 1998; Clarke & Chiappe, 2001; Chiappe, 2001, 2002; Clarke & Norell, 2002, 2004; Clark, Norell & Makovicky, 2002; Chiappe & Dyke, 2002; Maryanska, Osmólska & Wolsan, 2002; Pisani *et al.*, 2002; Snively, Russell & Powell, 2004; Mayr, Pohl & Peters, 2005; Zhou & Zhang, 2005).

Critical for empirical rooting of ingroup taxa, as opposed to hypothetical ancestors or other synthetic means of proposing polarities, this congruence lends credence to assessments of polarities of characters at the most basal of neornithine nodes (e.g. the divergence of neognathous from palaeognathous taxa). Crocodylians fell as predicted among the basal Archosauria (Larhammar & Milner, 1989; Hedges, 1994). Principal exceptions from a growing consensus of palaeontologists were reversed positions or irresolution within two pairs (Fig. 12): (i) Troodontoidea (*Troodon* and *Saurornithoides*) and Dromaeosauroidea; and (ii) *Rahonavis* and *Apsaravis*, the latter couplet being equally parsimonious whether paraphyletic to other taxa or as sister taxa. Details of positions among outgroups are of secondary interest here, but it is noteworthy that the few instances of incongruence with other studies were associated with exceptionally poorly supported nodes or polytomies in the present work (Fig. 12). It is likely that the generally lower support indices among pre-Neornithes reflect missing key taxa and poor preservation of those coded.

## Neornithes

‘… it is probable that the majority of living genera [of birds] were in existence by the end of the Tertiary… . Most, perhaps all, of the [modern] orders of birds had become established by the end of the Eocene.’ (Brodkorb, 1971a: 42)

‘The phylogenetic position of Palaeogene birds thus indicates that diversification of the crown-groups of modern avian “families” did not take place before the Oligocene, irrespective of their relative position within Neornithes (crown-group birds).’Mayr (2005a: 515)

Strong support for monophyly of the Neornithes (Table 2; Figs 10, 11) was conferred. Notable, however, in the present reconstruction was its poor congruence with the ‘tapestry’ depicted by Sibley & Ahlquist (1990), in which only three higher-order taxa – their Ratitae, Galloanserae and Procellarioidea, and monophyly of one contentious order (Caprimulgiformes) – were in significant agreement with corresponding clades in the present analysis. Points of disagreement, however, were abundant and included much of the topological (diagrammatic) structure in the two works, and notably included the following groupings depicted by Sibley & Ahlquist (1990: fig. 4A): (i) monophyly of {Ratitae, Galloanserae}; (ii) provisional, exceptionally basal placement of *Turnix*; (iii) very basal positions and interposition of Piciformes, Coraciiformes, Coliiformes, Trogoniformes and Passeriformes; (iv) multiple discrepancies associated with hypotheses of polyphyly of Pelecaniformes and Ciconiiformes, and (v) inclusion of some Gaviiformes, Podicipediformes, Sphenisciformes and Falconiformes among these groups. Topological dichotomies that hierarchically group modern orders of Neornithes were sought (Fig. 10), and these formed the ordinal basis for a higher-order classification (Appendix 1).

In the following, descriptions of findings, statistics of support, etc., were presented in figures, and reference to these was employed in place of repetition of metrics in the text. Consequently, readers are directed to the appropriate figures and tables where narratives refer to robustness, support and relative parsimony of alternative hypotheses.

## Modern palaeognathous birds

This analysis revealed the relationships among the palaeognathous birds to be exceptionally resolved, well supported, virtually unambiguous, empirically rich, markedly traditional, and supported by an unprecedented sample of outgroups. The ratites or flightless modern palaeognathous birds have been the subject of more anatomical and molecular study than any other avian group, an important motivation for which concerned diagnoses of plesiomorphic and apomorphic morphological characters in a group widely recognized to represent an early branch among Neornithes but for which useful outgroups were lacking (Balouet, 1984; Zusi, 1993). Basal polarities of characters of plesiomorphic condition among modern and closely related fossil palaeognathous taxa (Houde & Olson, 1981; Houde, 1988; Leonard *et al.*, 2005) awaited resolution by means of the most primitive Aves, many recovered only recently (Appendix 1).

Taxonomically orientated anatomical studies, emphasizing ratites or more inclusive in scope, ensued during the 19th and 20th centuries (Fürbringer, 1888; Feduccia, 1980; Houde & Haubold, 1987), and investigations of phylogenetic emphasis were among the earliest for Neornithes (Verheyen, 1960a; Sibley & Ahlquist, 1972; Cracraft, 1974a; Wattel, Stapel & de Jong, 1988). In some cases, inference of the primary grade of divergences of palaeognathous, galloanserine and other neognathous taxa aided in the recovery of historical patterns and broad outlines of phylogeny of palaeognathous taxa, patterns that were to prove beyond the limits of mtDNA for resolution (Härlid, Janke & Arnason, 1997, 1998).

Most prior studies regardless of method – notably excepting early works conceptually confined by the dated biogeographical paradigm of static continents (Briggs, 2003) or phenetic perspectives on affinities (McDowell, 1948; de Beer, 1956; Storer, 1960a, 1971a, b; Sibley & Frelin 1972) – have hypothesized that the palaeognathous birds are the sister-group of other Neornithes, the Tinamiformes are the sister-order of the ratites among palaeognathous taxa (Caspers, Wattel & de Jong, 1994; de Kloet & de Kloet, 2003), and accordingly the ratites are monophyletic (Bock, 1963b; Prager *et al.*, 1976; Stapel *et al.*, 1984; Bock & Bühler, 1988; Härlid *et al.*, 1997; Lee *et al.*, 1997; Van Tuinen, Sibley & Hedges, 1998; Dyke, 2001a; Dyke & Van Tuinen, 2004; Slack *et al.*, 2006a, b). These findings counter early disputes based in part on biogeography, isolated interpretations of fossils (Houde & Olson, 1981), speculations regarding heterochrony (Feduccia, 1985) and (subsequently admitted) analytical anomalies (Härlid & Arnason, 1998). Notable in the last of the foregoing categories was the initial inference of a sister-relationship between a neognathous group comprising the Galliformes and Anseriformes and the palaeognathous birds by Sibley & Ahlquist (1990), a topology rendering at the outset the polyphyly of neognathous taxa; subsequently these authors depicted the neognathous birds as monophyletic.

**Figure 7:**
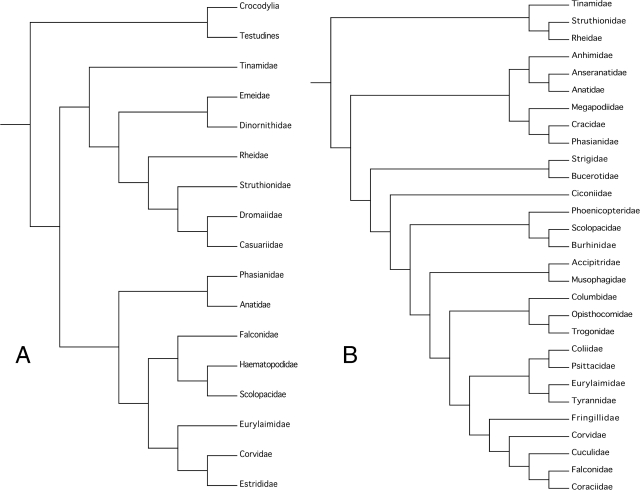
Molecular phylogenetic trees proposed in previous studies (see Fig. 1 for details), VII. A, Paton *et al*. (2002); B, Sorenson *et al*. (2003).

**Figure 8:**
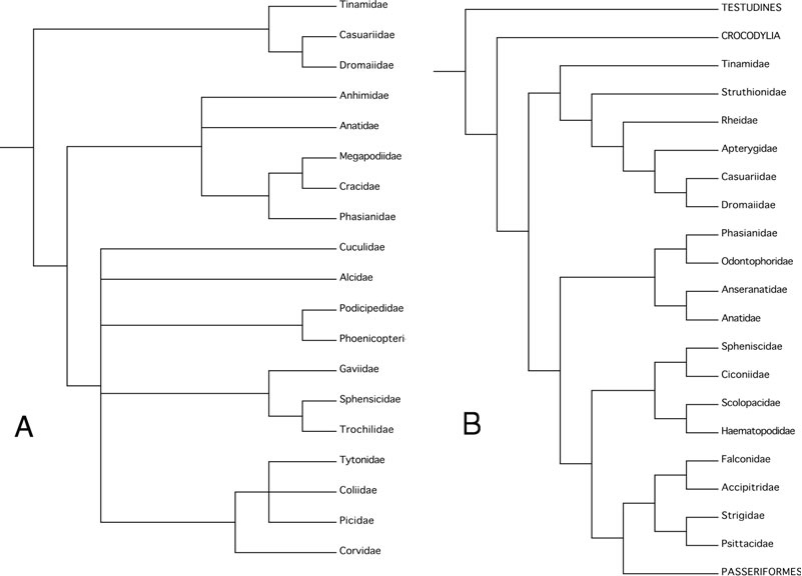
Molecular phylogenetic trees proposed in previous studies (see Fig. 1 for details), VIII. A, Chubb (2004a); B, Harrison *et al*. (2004).

**Figure 13:**
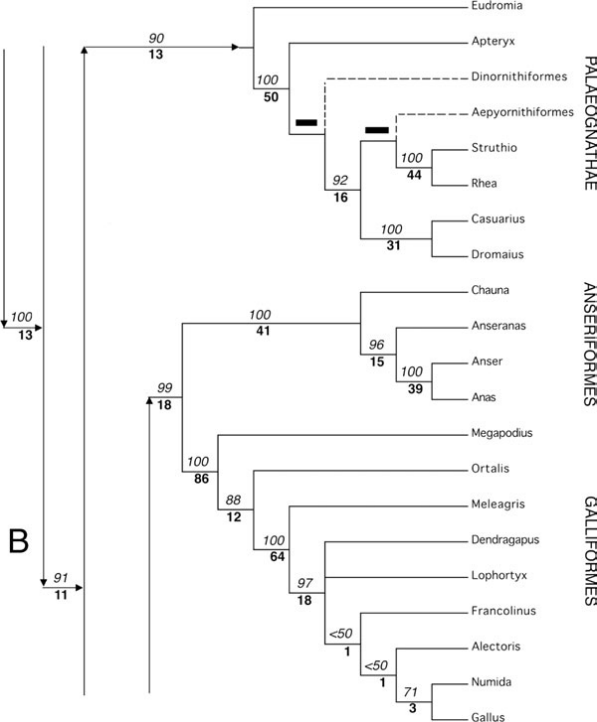
Detailed segment of strict consensus tree of all MPTs recovered in present study. Part B. Neornithes: Palaeognathae and Galloanserae. Nodes are labelled above by percentages of bootstrapped replicates in which node was retained (italics), and below by Bremer support indices (bold type).

Monophyly of the Tinamiformes was supported by the molecular analyses by Paton *et al*. (2002) and Harrison *et al*. (2004), but minimal taxonomic sampling diminished the generality of these inferences. Sister-group relationships of palaeognathous orders – Struthioniformes and Rheiformes, and Dromaiiformes and Casuariiformes – were supported strongly here (Fig. 13) and elsewhere (Lee, Feinstein & Cracraft, 1997; Leonard, Dyke & Van Tuinen, 2005). A minority of earlier findings (Figs 7A, 8B) provided weak evidence of paraphyly of the Struthioniformes and Rheiformes with respect to a sister-grouping of Dromaiiformes and Casuariiformes and also provided weak support for the Apterygiformes as as sister-group to the latter (Van Tuinen, Sibley & Hedges, 2000; Cooper *et al.*, 1992, 2001; Paton *et al.*, 2002; Harrison *et al.*, 2004). Despite support indices suggestive of robustness in several of the molecular works, questions regarding Bayesian bootstrap values (Simmons, Pickett & Miya, 2004) justify caution in such assessments.

The Apterygiformes, herein placed as sister-group to all other ratites (Fig. 13), have been inferred to occupy a marked diversity of positions in prior studies (Cracraft, 1974a, 2001; Lee *et al.*, 1997; Cooper *et al.*, 2001; Haddrath & Baker, 2001; Paton *et al.*, 2002; Harrison *et al.*, 2004). Also, the position of the Apterygiformes relative to the extinct Dinornithiformes varied (Vickers-Rich *et al.*, 1995). The Apterygiformes are the most speciose and genetically subdivided of extant orders of ratites (Baker *et al.*, 1995; Burbridge *et al.*, 2003), but are significantly less diverse than the formerly sympatric Dinornithiformes.

The position of the Dinornithiformes also remains a point of controversy, in part because of missing data for this extinct, diverse group; monophyly and relationships among members have been confirmed (Baker *et al.*, 2005). Cracraft (1974a, 2001) considered the Dinornithiformes to be the sister-group of the Apterygiformes, contrary to Cooper *et al*. (1992, 2001), Van Tuinen *et al*. (1998, 2000), Haddrath & Baker (2001) and the present provisional inferences. In most respects, the topologies for ratites inferred by Lee *et al*. (1997) and Dyke & Van Tuinen (2004: fig. 4) most closely approximated that inferred here (Fig. 13).

Missing data for two orders of ratites – Dinornithiformes and Aepyornithiformes – proved analytically problematic if included unconditionally with extant ratites. Unrestricted analysis of these extinct, moderately related, highly divergent, sparsely coded lineages resulted in a suspicious placement of these two orders as sister taxa. The large numbers of missing data in the two extinct lineages, many lacking in both taxa, prompted two alternative analyses to be performed. Global searches of Dinornithiformes (excluding the poorly known Aepyornithiformes) and placements within the MPT as backbone-constraint placed the moas to be the sister-group of other ratites exclusive of Apterygiformes (Fig. 13), contrary to a sister-relationship between these New Zealand endemics as advocated by Cracraft (1974a, 2001). By backbone-constraints or exclusion of the Dinornithiformes, the Aepyornithiformes were placed as the sister group of the clade comprising Struthionidae and Rheidae (Fig. 13).

## Galliformes and Anseriformes: land and water fowl

### Interordinal relationships

The sister-group relationship between the Galliformes and Anseriformes, reaffirmed here (Fig. 13), was inferred previously by Cracraft (1981, 1988), Cracraft & Mindell (1989), and substantiated thoroughly using morphological (Dzerzhinsky, 1995; Caspers *et al*., 1997; Livezey, 1997a, 1998a; Cracraft & Clarke, 2001; Dyke, 2003; Mayr & Clarke, 2003) and molecular data (Bleiweiss *et al.*, 1994, 1995; Groth & Barrowclough, 1999; Van Tuinen *et al.*, 2000, 2001; Cracraft, 2001; Prychitko & Moore, 2003; Chubb, 2004a; Harrison *et al.*, 2004; Simon *et al.*, 2004; Smith, Li & Zhijian, 2005). However, marginally supported counter-proposals persist (Ericson, 1996, 1997; Ericson, Parsons & Johansson, 1998; Bourdon, 2005).

### Anseriformes

Within the waterfowl (Anseriformes), sequential sister-group relationships of the Anhimidae, Anseranatidae and Anatidae, respectively, was previously demonstrated by Livezey (1997a) and confirmed here (Fig. 13). Monophyly of the morphologically diverse and speciose Anatidae, including the true geese (Anserinae) and typical ducks (Anatinae), is essentially beyond dispute (Livezey, 1986). There exist departures from this arrangement by a minority of workers (Olson & Feduccia, 1980a; Sraml *et al.*, 1996), but this topology has been substantiated using diverse evidence (Livezey, 1986, 1997a; Quinn, 1992; Donne-Goussé*et al.*, 2002). The historical hypothesis placing the Phoenicopteridae within the Anseriformes (Table 1) was among the early casualties of formal phylogenetics (Livezey, 1997a, 1998a).

### Galliformes

The pioneering myological works by Hudson, Lanzillotti & Edwards (1959) and Hudson & Lanzillotti (1964) provided early hints concerning relationships of Galliformes, but unfortunately these surveys were not cladistic and followed Peters (1934) in considering unique *Opisthocomus* as an aberrant galliform. Studies of galliform fossils continue to be phenetic in approach (Mourer-Chauviré, 2000; Göhlich & Mourer-Chauviré, 2005). Fortunately, this pattern is likely to change with the increasingly common phylogenetic analyses of galliforms (Dyke, Gulas & Crowe, 2003) and an improved fossil record (Mayr & Weidig, 2004; Mayr, 2005a).

In the present work, relationships of two families within the Galliformes – Megapodiidae (Birks & Edwards, 2002) and Cracidae (Pereira & Baker, 2004; Grau *et al.*, 2005) as mutually monophyletic, sequential sister-groups to all remaining galliforms – agree with placements by other investigators (Prager & Wilson, 1976; Cracraft *et al.*, 2004). Some workers (Hudson *et al.*, 1966), however, suggested a sister-group relationship between the two families (superfamily Cracoidea), as opposed to placement as successive sister-groups (paraphyletic) to other galliforms (Fig. 13).

The robust placement of Meleagrididae as sister-group to the Phasianidae *sensu lato* in the present work (Fig. 13) opposes inclusion of the family among the enormous complement of other galliforms (reviewed by Sibley & Ahlquist, 1990). The present finding also differs with the indeterminate placement of this distinctive group from most galliforms by Dyke *et al*. (2003). Dyke *et al*. (2003: fig. 3) depicted the Megapodiidae and Cracidae as basal, successive sister-groups to the diverse and speciose ‘Phasianoidea’; the latter group included *Numida* and *Acryllium* (Numidinae) as members of a polytomous assemblage immediately basal to *Meleagris*, *Agriocharus*, Tetraonidae, and a clade comprising 39 taxa of other galliforms inviting taxonomic subdivision. Most of the large-bodied genera of phasianoids (e.g. *Gallus*, *Phasianus*) and the ‘Old World quail and partridges’ were among a large, basal polytomy of the ‘phasianoids’ exclusive of the guineafowl (Numidinae). Some of the nodes within this large group, including those resolving Meleagridae and Tetraonidae relative to megapodiid and cracid galliforms, were not sustained by Dyke *et al*. (2003: fig. 3) in a strict consensus of 1700 MPTs based on 102 characters. Also, the tree inferred here (Fig. 13) departed from those recovered using molecular data (Dimcheff, 2002; Dimcheff, Drovetski & Mindell, 2002).

**Figure 3:**
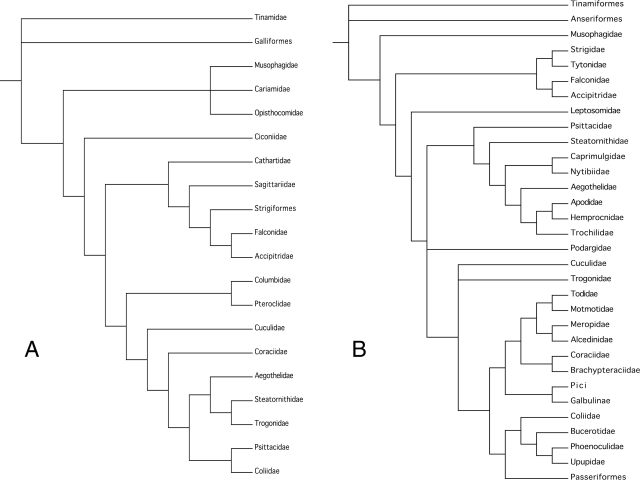
Morphological phylogenetic trees proposed in previous studies (see Fig. 1 for details), III. A, Mayr (2005b); B, Mayr (2005f: fig. 9), excluding fossils *Prefica* and *Paraprefica*.

The vast majority of galliform taxa are members of a morphologically conservative group (Holman, 1961), many formerly included among the Perdicidae or Odontophoridae (Sibley & Ahlquist, 1990). These taxa also posed problems of resolution in the present work (Fig. 13), and nodes among these taxa were sufficiently weak as to permit alternative local topologies (i.e. a terminal polytomy). Armstrong, Braun & Kimball (2001) found that mitochondrial and nuclear DNA similarly resolved groupings within a sparse but broad sample of Galliformes. Basal nodes of the latter taxa are broadly consistent with some higher-order topologies (Prager & Wilson, 1976; Helm-Bychowski & Wilson, 1986; Crowe *et al.*, 1992; Kimball *et al.*, 1999; Gutiérrez, Barrowclough & Groth, 2000; Lucchini *et al.*, 2001; Dimcheff *et al.*, 2002; Pereira, Baker & Wajntal, 2002). The single exception among this group (based on included genera) is the strongly supported sister-group relationship between *Gallus* (Phasianidae) and *Numida* (Numidinae). The Numidinae were inferred to be the sister-group of the Phasianidae by Kimball *et al*. (1999) and Pereira & Baker (2006a).

## Marine assemblage

A diversity of mutually distinctive groups of aquatic birds have been the focus of much early speculation regarding the potentially misleading effects of similarities of locomotion leading to morphological convergence. Most evocative of these speculations concerned the Gaviiformes and Podicipediformes (e.g. Stolpe, 1935; Storer, 1956, 1960b), foot-propelled diving specialists that prompted arguments based on phenetics, assumptions of ancestral status for fossils, simplistic proposals of evolutionary trends and (most fundamentally) a failure to meet conventional standards of phylogenetic inference. These shortcomings notwithstanding, such proposals from this era gave rise to a general and uncritical acceptance of rampant convergence uniquely afflicting morphological characters, claims that persist to the present day.

Various alliances among the Gaviiformes, Podicipediformes and Procellariiformes were suggested by Mayr & Amadon (1951), and proved consistent with myological data analysed by  McKitrick (1991a,  b) and molecular patterns recovered by Watanabe *et al*. (2006). A relationship between the Gaviidae and Charadriiformes was considered plausible by Storer (1956). Without explanation, however, Storer (1971b) listed the loons and grebes together immediately following the Charadriiformes, in apparent contradiction to his previous opinion. Foreshadowing a natural radiation of marine birds, Ho *et al*. (1976) inferred a comparatively close relationship of the Sphenisciformes with other primarily marine orders, and fossil evidence for loons – of only marginal quality, optimistic appraisals by Olson (1992a) and Mayr (2004a) notwithstanding – suggests an early origin at least for the Gaviiformes. A phylogenetic alliance among the Sphenisciformes, Procellariiformes, Gaviiformes and Podicipediformes was substantiated as well by Cracraft (1982a), and this was indicated by Nunn & Stanley (1998) and Slack *et al*. (2006a) on molecular grounds.

**Figure 14:**
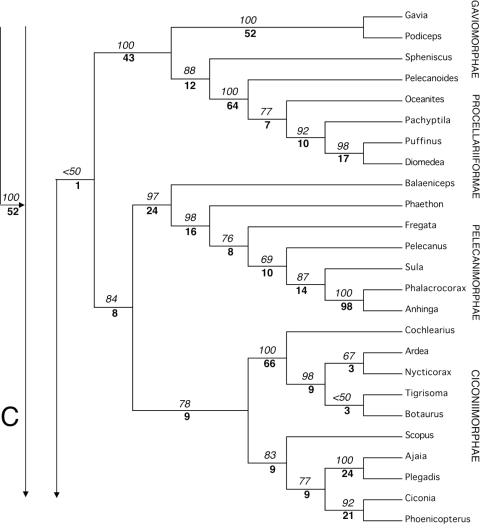
Detailed segment of strict consensus tree of all MPTs recovered in present study. Part C. Neornithes: nodes are labelled above by percentages of bootstrapped replicates in which node was retained (italics), and below by Bremer support indices (bold type).

The comparatively robust skeletal elements of penguins predispose them to fossil preservation, and recently recovered remains hold promise for stratigraphic chronology (Slack *et al.*, 2006b). The clade of basal marine taxa inferred herein evolved myriad modes of foraging (Storer, 1971a): (i) Gaviiformes and Podicipediformes being extremely specialized foot-propelled diving birds; (ii) Sphenisciformes and Pelecanoididae (Procellariiformes) being wing-propelled diving birds, submarine ‘flight’ of the former rendering members aerially flightless (Livezey, 1989a); and (iii) Procellariiformes, comprising hover-foraging Oceanitidae and other families combining wind-powered gliding and plunge-diving (Del Hoyo, Elliott & Sardgatal, 1992). Some fossil groups remain of uncertain ordinal affinity – e.g. the wing-propelled Plotopteridae (Olson & Hasegawa, 1979, 1996; Olson, 1980; Goedert, 1988; Goedert & Cornish, 2002; Mayr, 2004b) – and did not merit analysis herein, where states for cranial characters are critical but specimens are woefully incomplete. Early descriptions suggested the inclusion of the Plotopteridae among Pelecaniformes is competitive with an alternative relationship to Sphenisciformes for which pectoral similarities were emphasized (Mayr, 2004b). Dissent regarding the ordinal relationships of the Plotopteridae is consistent, to a point, with the interordinal relationships of the Pelecaniformes and Sphenisciformes inferred herein (Fig. 14).

Monophyly of the Sphenisciformes seldom has been doubted, and resolution of relationships among modern and fossil species was achieved (Ksepka, Bertelli & Giannini, 2006), but the position of this distinctive marine group remains a long-standing controversy. This duality of distinct synapomorphy and symplesiomorphy underlies a number of classificatory problems of Aves, in which marked distinction of groups tends to confound comparisons with other groups. Of the alternatives proposed, an affinity with the Procellariiformes has received broadest support, both in the present analysis (Fig. 14) and elsewhere (Cracraft, 1981, 1986, 1988).

Despite agreement with the inferences by Cracraft (1982a), it is predictable that strong confirmation of a sister-group relationship between the Gaviiformes and Podicipediformes (Fig. 14) herein will engender concerns of artefactual pairing by convergence (Storer, 1956, 1971a, 2000, 2002). Storer (2002: 16) felt that the non-phylogenetic work by Stolpe (1935) ‘… demonstrated that the similarities among the loons, grebes, … resulted from convergent evolution …’ The inclusion of the Mesozoic Hesperornithiformes with modern Gaviiformes and Podicipediformes by Cracraft (1982a), a finding not supported here (Figs 10, 14), was the inference subjected to greatest criticism. Obvious similarities of form and life history have prompted exceptional attention to differences between the two orders (e.g. Sibley & Ahlquist, 1972: table 1), tallies without benefit of polarities or phylogenetics. In many cases, these rationalizations are undermined with respect to functional comparisons, e.g. the Gaviidae employ feet for primary propulsion but also use their wings (Olson, 1985), and members of the two orders also differ in the movements typical of the pelvic limb (Storer, 1956). Pairing of the Gaviiformes with the Podicipediformes as sister-groups has been championed by Cracraft (1982a, 1988), a proposal not without opposition (e.g. Storer, 1956, 1960b, 1971a; Sibley & Ahlquist, 1972, 1990). Additional support for this ordinal pairing has been reported ( Cracraft & Mindell, 1989; Bourdon, Boya & Iarochène, 2005), but most other analyses excluded one or both of these key orders, rendering comparisons among such works regarding these orders impossible.

Without a consensus regarding a relationship between the Podicipedidae and Gaviidae, the former have been the subject of several extraordinary proposals, based on relatively weak evidence or mere speculation. Olson (1985: 168), under the subheading ‘Family *Incertae Sedis* Podicipedidae’, stated: ‘In looking beyond their obvious specializations for diving, I cannot see that the grebes (Podicipedidae) would be out of place in the Gruiformes.’ A more precise proposal for the latter is a possible affinity on myological grounds with the gruiforms *Rhynochetos* and *Eurypyga* (Zusi & Storer, 1969). An apparent variant of this speculation was a possible relationship with the Heliornithidae and the closely related Rallidae (Beddard, 1893; Olson, 1985; Houde, 1994). Also, a tenuous alliance between the Podicipedidae and Cuculidae was depicted by Van Tuinen *et al*. (2000), but subsequent works have failed to support this grouping. Another position recently inferred for the Podicipediformes relates to the Phoenicopteridae (Van Tuinen *et al.*, 2001; Mayr, 2004c), a proposal considered further below.

In most respects, inferences herein regarding the Procellariiformes were among the least contentious for the marine assemblage, whether in comparison with traditional (Kuroda, 1954) or modern reconstructions (Nunn & Stanley, 1998; Kennedy & Page, 2002; Watanabe *et al.*, 2006). A moderate departure from traditional arrangements is the finding herein of the Diomedeidae (albatrosses) as comparatively derived, with other Procellariiformes paraphyletic to the typical Procellariidae (Austin, 1996; Gómez-Díaz *et al.*, 2006) and Diomedeidae (Nunn *et al.*, 1996).

## Pelecans and allies: totipalmate birds

The totipalmate or pelecaniform birds, as traditionally defined, remain a higher-order group of extraordinary controversy, but in reality the suite of unifying characters, stressed by Beddard (1898), has been expanded for decades beyond the totipalmy cited as sole uniting anatomical character for the order by Sibley & Ahlquist (1972). Polyphyly of the order was inferred subsequently by Sibley & Ahlquist (1990) and Hedges & Sibley (1994). The status of the Pelecaniformes has been debated since the core assemblage was included in widely recognized classifications (Mayr & Amadon, 1951; Wetmore, 1930, 1960), and points of controversy include those of monophyly, content and interordinal position, as empirically derived from metric (Verheyen, 1960b), neontological (Cracraft, 1985), palaeontological (Bourdon, 2005; Bourdon *et al.*, 2005) and molecular perspectives (Siegel-Causey, 1997; Farris *et al.*, 1999).

The exceptional heterogeneity of traditionally included families – e.g. frigatebirds, gannets and pelicans – render questions of membership especially problematic. Perhaps most intriguing of the debated memberships is that of the shoebill or *Balaeniceps* (Reinhardt, 1860, 1862; Cottam, 1957; Feduccia, 1977a; Mayr, 2003a). Purportedly intermediate features of ‘stork-like’ and ‘pelican-like’ forms (Van Tuinen *et al.*, 2001; Bourdon *et al.*, 2005) have extended to proposals of pelecaniform affinity of the hammerkop (Scopidae). In agreement with the present analysis, the consensus of available phylogenetic works places the distinct Phaethontidae as sister-group to other pelecaniforms exclusive of *Balaeniceps* (Mayr & Clarke, 2003), with an alternative position hypothesized for the Phaethontidae as an exceptional plesiomorph allied to some pelecaniforms and the Procellariiformes (Bourdon *et al.*, 2005). The present study also resolved *Balaeniceps* as sister-group to the clade comprising Phaethontidae and other (traditional) Pelecaniformes. *Scopus* was not inferred here to be closely related to the Pelecaniformes (Fig. 14), *contra*Mayr (2003a).

Relationships among traditional Pelecaniformes (excluding *Balaeniceps*), inferred cladistically by Cracraft (1985: figs 6, 7), agreed with the inferences presented herein (Figs 10, 14), whereas comparisons between the studies with respect to the orders Sphenisciformes, Gaviiformes, Podicipediformes and Procellariiformes were not possible. Sibley & Ahlquist (1990) proposed a ‘four-fold’ polyphyly of Pelecaniformes among the most notable departures of their analysis from contemporary arrangements, whereas several other traditional elements were conserved in their scheme. Hedges & Sibley (1994), based on an analysis impoverished in both data and taxa, also suggested polyphyly of taxa traditionally considered pelecaniform in a work remonstrated by Farris *et al*. (1999). Syntheses by Van Tets (1965) and Siegel-Causey (1997: fig. 6.3) reaffirmed ordinal monophyly (exclusive of Phaethontidae) using morpho-ethological data, whereas molecular reconstructions violated ordinal monophyly by topologically variable inclusions of the Diomedeidae, Procellariidae and Cathartidae (Siegel-Causey, 1997: fig. 6.2). One minor departure from tradition by Sibley & Ahlquist (1990) was a terminal triad in which the Phalacrocoracidae were placed as sister-group to the Anhingidae and Sulidae.

**Figure 6:**
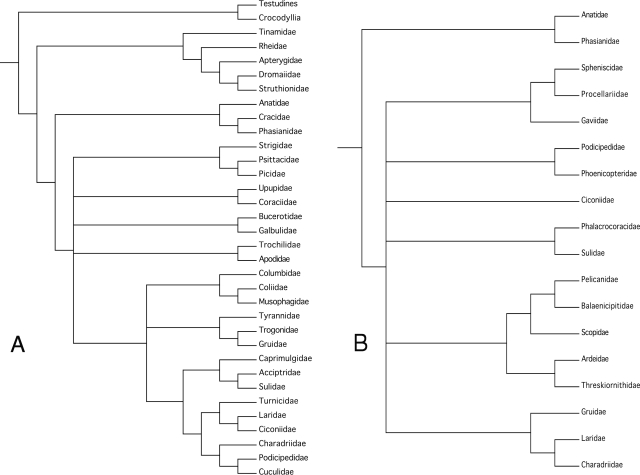
Molecular phylogenetic trees proposed in previous studies (see Fig. 1 for details), VI. A, Van Tuinen *et al*. (2000); B, Van Tuinen *et al*. (2001).

Kennedy & Spencer (2004) weakly confirmed monophyly of the traditionally constituted order, in part by use of appropriate outgroups but despite heterogeneous taxonomic sampling of ingroup families. Three weakly resolved departures by Kennedy & Spencer (2004) from the hypothesis inferred herein (Fig. 14) were: (i) reversal of the positions of the Phaethontidae relative to the Fregatidae + Pelecanidae; (ii) a sister-relationship between the Pelecanidae and Phaethontidae; and (iii) paraphyly of Phalacrocoracidae and Anhingidae to the Sulidae.

The Phalacrocoracidae and Anhingidae – families long considered closely related and strikingly similar in external and skeletal aspects (Siegel-Causey, 1988) – have been subjected to unexpected hypotheses of relationship. A series of related papers (Kennedy, Spencer & Gray, 1996; Kennedy, Gray & Spencer, 2000; Kennedy & Spencer, 2000, 2004; Kennedy *et al.*, 2005), based on limited taxonomic representation of pelecaniform families and unconventional analytical methods, mustered mtDNA sequences and behavioural data that favoured paraphyly of these two families to the Sulidae, also inferred phenetically by Sibley & Ahlquist (1990). Based on the present analysis (Table 3), however, a sister-group relationship between Phalacrocoracidae and Anhingidae is strongly favoured.

**Table 3:** Alternative topological inferences and minimal differences in tree length (additional steps) relative to placements in MPTs (Figs 11–17), conditional on other topological alterations being prohibited (optimizations of characters thereon permitted). Higher-order taxa correspond to classification proposed in Appendix 1

Taxon	Alternative hypothesis*	Δ length	References
Palaeognathae	∪ Galloanseromorphae	54	Sibley & Ahlquist (1990)
Ratitae (global)†	Δ topology	31	Cracraft (1974a)
Ratitae (local)†	Δ topology	63	Cracraft (1974a)
	Δ topology‡	17	Cooper *et al*. (2001)
	Δ topology‡	13	Haddrath & Baker (2001)
Galloanserimorphae	Polyphyly§	[19]	Bourdon *et al*. (2005)
Galliformes	Polyphyly	90	Dyke *et al*. (2003)
Megapodiidae	∪ Cracidae	10	Dyke *et al*. (2003)
Meleagrididae	∪ Phasianidae	20	Dyke *et al*. (2003)
Anseriformes	Δ familial topology	54	Olson & Feduccia (1980a); Livezey (1997a)
Anhimae	∪∨⊂ Galliformes	41	Olson & Feduccia (1980a); Livezey (1997a)
Gaviomorphae	∪ Charadriomorphae	72	Storer (1956); Olson (1985)
Podicipediformes	∪ Phoenicopteridae	146	Mayr & Clarke (2003); Mayr (2004a)
	∪ Charadriomorphae	54	Storer (1956)
	∪ Eurypygidae	182	Zusi & Storer (1969)
	∪ Ralliformes	159	Olson (1985); Houde (1994)
Pelecaniformes	Δ topology	344	Kennedy & Spencer (2004: fig. 1B)
Sulae	Δ topology	125	Kennedy *et al*. (2005: fig. 8)
Balaenicepitidae	¬∪ Pelecaniformes	30	Cracraft (1985); Mayr (2003a)
Scopidae	∪∨⊂ Pelecaniformes	23	Mayr (2003a)
Threskiornithidae	∪∨⊂ Charadriiformes	174	Olson (1978)
Ardeidae	⊂ (Turnices ∪ Eurypygae)	75	Olson (1978)
Phoenicopteridae	∪ Anseriformes	107	Feduccia (1976, 1977b); Hagey *et al*. (1990)
	∪ Cladorhynchini	154	Olson & Feduccia (1980b)
Gruiformes (traditional)	Monophyly¶	11	Livezey (1998b)
Charadriiformes	Δ topology	60	Strauch (1978) *fide*Chu (1995: fig. 1)
	Δ topology	106	Sibley & Ahlquist (1990) *fide*Paton *et al*. (2003)
	Δ topology	82	Chu (1995: fig. 8), excluding *Ibidorhyncha*
Mesitornithidae	∪ Cuculiformes	107	Mayr & Ericson (2004)
Strigiformes	∪ Caprimulgiformes	43	Hoff (1966)
Cathartidae	∪∨⊂ Ciconiiformes	112	Ligon (1967); Rea (1983); Avise *et al*. (1994a)
Opisthocomidae	∪∨⊂ Galliformes	120	Hudson *et al*. (1959); Hudson & Lanzillotti (1964)
	∪ Cuculiformes**	22	Avise *et al*. (1994b); Hughes & Baker (1999)
Caprimulgiformes	Polyphyly	31	Mayr (2002a, b)
	∪ Cypselomorphae	42	Mayr (2002a, 2003c, 2004d, 2005f, g)
Aegothelidae	∪ Apodiformes	31	Mayr (2002a, 2003c, 2004d, 2005f, g)
Steatornithidae	∪ Trogoniformes	102	Mayr (2003b)
Hemiprocnidae	∪ Apodidae, monophyly	5	Sibley & Ahlquist (1990: fig. 361)
Apodidae	∪ Passeri (Hirundinidae)	193	Shufeldt (1889b); Van Tuinen (2002)
Galbulae	∪∨⊂ Coraciiformes	20	Olson (1983a)
Coraciiformes	Δ topology, ∈ Trogoniformes	199	Lowe (1946); Maurer & Raikow (1981)
Coracii	Δ topology	64	Cracraft (1971b)
*Menura*	∪∨⊂ Passeri	11	Irestedt *et al*. (2001); Barker *et al*. (2002)

* Set-symbolism coopted for concise statement of phylogenetic hypotheses, as follows: ∪, sister-group (disjoint) union; ⊂, included as subclade; ∈, included as a member taxon; ∨, or; Δ, change in; ¬, not (negation of predicate argument).

† Local optima for Aepyornithiformes and Dinornithiformes (as bi-ordinal sister-group to ratites exclusive of Apterygiformes) and global optima (former as sister-group to Struthionidae and Rheidae, latter as sister-group to ratites exclusive of Apterygiformes).

‡ Comparisons excluded effects due to differences in outgroup taxa, as well as tentatively placed Aepyornithiformes.

§ Doubtful comparability given differences in taxonomic samples between studies.

¶ Corresponds to that proposed by Livezey (1998b), exclusive of Pedionomidae and fossil gruiforms (Cracraft 1969, 1971a, 1973a).

** Alternative hypothesis compared sister-grouping with Cuculiformes exclusive of Musophagidae.

## Storks, herons and allies

‘Wading birds’, as delimited here, comprise the typically long-legged, long-necked storks and herons, and exclude the morphologically reminiscent cranes and allies (Gruiformes) and the potentially allied shorebirds (Charadriiformes). Highest-order nodes resolved in the present study defined a primary division of (i) ‘herons’ from (ii) ‘storks’ and allies as sister-groups (Fig. 14). Among the ‘storks’, *Scopus* is the sister-group to other members, the latter comprising clades partitioning the (i) ibises and spoonbills, and (ii) flamingos and typical storks. Within the ‘herons’, the only notable finding is the placement of *Cochlearius* as sister-group to other herons (Fig. 14), an inference consistent with traditional classifications (e.g. Wetmore, 1960) and earlier findings (Cracraft, 1967a; Sheldon, Jones & McCracken, 2000).

Shufeldt (1901b) suggested affinities between the Phoenicopteridae (flamingos) and both the Anseriformes (waterfowl) and the Ciconiiformes (storks and traditional allies). Olson (1978) questioned the monophyly of the traditional Ciconiiformes on phenetic grounds, suggested charadriiform affinities of Phoenicopteridae and Threskiornithidae, and expressed uncertainty regarding the ordinal placement of the herons (Ardeidae). Van Tuinen *et al*. (2001), based on conventional molecular estimates and the phenetics of DNA–DNA hybridization, found no support for monophyly of the Ciconiiformes in an analysis including representatives from several other traditional groups. Molecular reconstructions by Slikas (1997), however, confirmed monophyly of the morphologically diverse, ‘true’ storks (*Scopus* and *Balaeniceps* not sampled), groupings that also were afforded significant ethological support (Slikas, 1998).

It has been hypothesized in recent years that the Phoenicopteridae may be the sister-group of the grebes (Podicipediformes), a proposal supported by tenuous molecular (Van Tuinen *et al.*, 2001) and morphological evidence (Mayr & Clarke, 2003; Mayr, 2004c; but see Storer, 2006). Given the variable viewpoints expressed regarding the Phoenicopteridae as well (Gadow, 1877; Shufeldt, 1889a; Feduccia, 1976, 1977a), this couplet offered the hope of dispensing with two challenging taxonomic placements by means of a single union, a circumstance not uncommonly an artefact of long-branch attraction (Philippe *et al.*, 2005). Both of these autapomorphic taxa have been subjected to classificatory confusion for more than a century (e.g. Weldon, 1883; Shufeldt, 1901b; Jenkin, 1957), with affinities of the flamingos considered plausible between either the Ciconiiformes or the Anseriformes. Despite robust support for the more traditional position in the present analysis (Tables 2, 3; Figs 10, 14) and the minimal evidence presented by others for the proposal of the Podicipediformes, the latter hypothesis merits examination on the grounds of its superficial implausibility and the marked rearrangements of higher-order avian relationships it would imply. Supplementary morphological support for a sister-group relationship between grebes and flamingos marshalled by Mayr & Clarke (2003), however, required the exclusion (in a second analysis) of the loons – heretofore the global sister-group of the grebes – to sustain the grouping in question. Both exclusion of the Gaviiformes and narrow sampling of characters and taxa with which the Phoenicopteriformes were evaluated by Mayr (2004c) weakened the resultant inferences regarding the relationships of flamingos.

Chubb (2004a: 148) recovered 50% and 78% bootstrap support for this taxonomic couplet in analyses of different partitions of the *ZENK* gene, and joined Van Tuinen *et al*. (2001) in the speculation that: ‘… because both grebes and flamingos are highly derived morphologically and adapted to unique aquatic niches, their potential evolutionary alliance has previously gone unnoticed.’ Unfortunately, this rationalization is vulnerable to criticism because: (i) modifications for foot-propelled diving of grebes are comparable with those of several other groups of Neornithes – e.g. some Anatidae (Oxyurini, Mergini), Gaviidae, Phalacrocoracidae and Anhingidae; and (ii) the ‘unnoticed alliance’ between grebes and flamingos recognized by Chubb (2004a) instead was countered by a number of apomorphies in each genus that are shared with other taxa – e.g. *Podiceps* with *Gavia*, *Phoenicopterus* with (other) Ciconiiformes. The present data set (Livezey & Zusi, 2006) supports the rejection of this novel proposal involving the grebes and flamingos (Table 3; Fig. 14), and suggests that the taxonomic proposal for the couplet by Sangster (2005) is premature.

## Cranes, rails, shorebirds and allies

**Figure 15:**
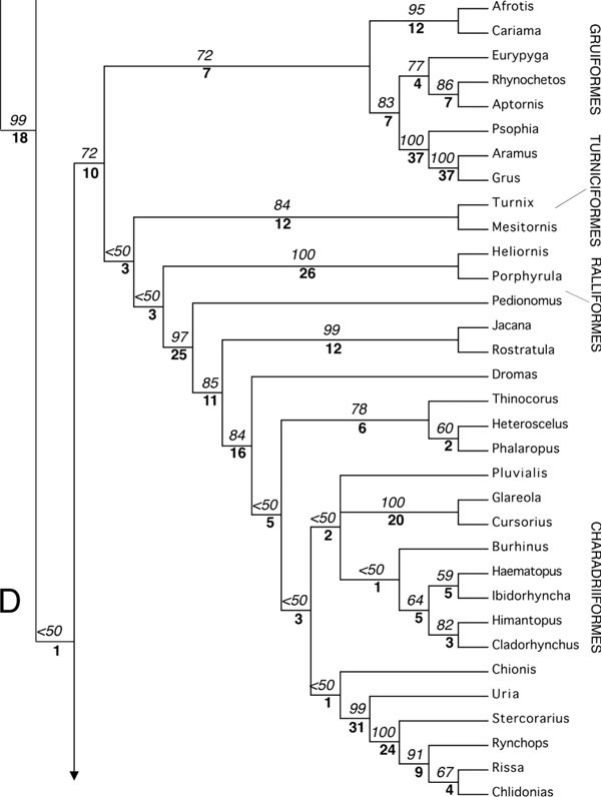
Detailed segment of strict consensus tree of all MPTs recovered in present study. Part D. Neornithes: Gruiformes and Charadriiformes. Nodes are labelled above by percentages of bootstrapped replicates in which node was retained (italics), and below by Bremer support indices (bold type).

The remaining long-legged, statuesque denizens of early successional, often wet habitats, together with the true shorebirds, compose the sister-group of remaining neornithine taxa (Fig. 15). These families, typically included within the traditional Charadriiformes and Gruiformes, have a long, perhaps unequalled history of debate in the ornithological literature (reviewed by Sibley & Ahlquist, 1972, 1990; Livezey, 1998b). Primary points of controversy concern the monophyly of the Gruiformes, and relationships between the taxa traditionally referred to the Gruiformes and the Charadriiformes; the latter order is known for especially great diversity in structure of the skull (Kozlova, 1961).

### Gruiformes and allies

In an analysis of phylogeny and flightlessness of the Rallidae (Livezey, 1998b, 2003b), the traditionally delimited Gruiformes appeared to be monophyletic when analysed with only limited outgroups. However, in the more extensive sampling of higher-order groups of the present analysis (Fig. 15), this assemblage was resolved to be paraphyletic to the Charadriiformes. Most families included among the Gruiformes have been the subject of comparatively intense debate with respect to taxonomic position, e.g. Sibley, Ahlquist & DeBenedictus (1993) prepared an addendum for the Rallidae and allied families, and Houde (1994) revealed the difficulties of resolving the phylogenetic position of the Heliornithidae within the order. Nonetheless, the order contributed to early perceptions of southern-hemispheric origins of many non-passeriform birds (Cracraft, 1982b).

In the present work, most families formerly included among the Gruiformes were inferred to be monophyletic (Fig. 15), forming a single clade within which a primary bifurcation established the first of two subclades comprising the Otididae (Pitra *et al.*, 2002) and Cariamidae (Livezey, 1998b). The second of the primary gruiform clades, and sister-group of the foregoing clade, comprised the sister-groups of (i) Eurypygae (i.e. Eurypygidae, Rhynochetidae and Aptornithidae as sequential sister-groups) and (ii) the nominate suborder Grues (i.e. Psophiidae, Aramidae and Gruidae as sequential sister-groups). New information on the Eocene fossil *Eogrus* (Cracraft, 1969; Clarke *et al.*, 2005a) is consistent with monophyly of the Gruidae inferred by other means (Krawjewski & King, 1996). With the exception of an alternative position hypothesized for the subfossil Aptornithidae (Livezey, 1994; Houde *et al.*, 1997), arrangements of these ordinally defining families have engendered only limited dissent (Mitchell, 1915; Livezey, 1994, 1998b).

Several families formerly included within the Gruiformes by Livezey (1998b), as detailed above, were inferred herein to be members of the sister-group of the Gruiformes, and specifically were resolved as two sequential sister-groups of the Charadriiformes (Fig. 15). Several of these have attracted an inordinate interest pertaining to phylogenetic position, diversity of form, intraordinal membership (e.g. Turnicidae) and manifestation of morphological intermediacy of others – e.g. Pedionomidae and Otididae (Gadow, 1891a; Bock & McEvey, 1969; Olson & Steadman, 1981). The present analysis provisionally placed the Turnicidae and Mesitornithidae as sister-taxa and the first of the two sequential sister-taxa (taxa paraphyletic) to the Charadriiformes (Fig. 15). Rotthowe & Starck (1998) agreed with both the present analysis and that by Livezey (1998b) regarding an affinity between the Turnicidae and Gruiformes, but Mayr & Ericson (2004) proposed a close relationship between the Mesitornithidae and Cuculiformes. The remaining sequential sister-group (lineage in this grade) comprised the Rallidae and its sister-group Heliornithidae (Fig. 15), a close relationship inferred both by Houde (1994) and Livezey (1998b), among others.

### Charadriiformes

The preceeding clades subtended a clade herein interpreted as comprising the Charadriiformes. The true shorebirds, as resolved here (Fig. 15), comprise families of comparatively obvious ordinal affinity and great apomorphy, and generally accepted as monophyletic (Strauch, 1978; Björklund, 1994; Chu, 1994, 1995; Moum *et al.*, 1994; Moum, Arnason & Arnason, 2002; Friesen, Baker & Piatt, 1996; Thomas, Wills & Székely, 2004a; Bridge, Jones & Baker, 2005). Relationships among several major groups of charadriiform birds have been inferred (e.g. Thomas, Wills & Székely, 2004b); however, the systematics of the group remains markedly controversial (Strauch, 1985; Christian, Christidis & Schodde, 1992; Paton *et al.*, 2002; Ericson *et al*., 2003a; Van Tuinen, Waterhouse & Dyke, 2004; Paton & Baker, 2006; Pereira & Baker, 2006b).

The present analysis established the monophyly of the Charadriiformes, of which the Pedionomidae constituted the sister-group to other members (Fig. 15). The latter finding represents a slight departure from the marginal inclusion of *Pedionomus* among Gruiformes and affinities of the genus with the charadriiform Jacanidae (Whittingham, Sheldon & Emlen, 2000) and Rostratulidae inferred by Livezey (1998b), and is consistent with the inferences by Olson and Steadman (1981) and Ericson (1997). Within the Charadriiformes, *Pedionomus* is the sister-group to: (i) the bifamilial couplet comprising the Jacanidae and Rostratulidae, (ii) the monotypic Dromadidae and (iii) a clade comprising Thinocoridae and the sister-families Scolopacidae (e.g. *Heteroscelus*) and Phalaropodidae (Fig. 15); and (iv) a terminal clade comprising two major subclades and multiple, only partially dichotomously resolved families (Fig. 15). These broad groupings bear notable similarities with the suborders defined by Lowe (1931a).

The remaining clade of the Charadriiformes comprises two major subclades, both of which are weakened by three marginally robust, defining nodes (Fig. 15). The first comprises in turn three lineages or subclades: (i) the Charadriidae; (ii) the sister-groups Cursorinae and Glareolinae (collectively constituting the Galreolidae); and (iii) a clade comprising the Burhinidae and its sister-group comprising two bifurcate clades, the Haematopodidae (united exclusively with monotypic *Ibidorhynchus*), and the Recurvirostridae (united exclusively with monotypic *Cladorhynchus*). The other major, pectinate subclade within the Charadiformes comprises, respectively, the sequential sister-groups Chionididae, Alcidae, Stercorariidae, Rynchopidae and Laridae (Fig. 15).

## Birds of prey–diurnal and nocturnal

**Figure 16:**
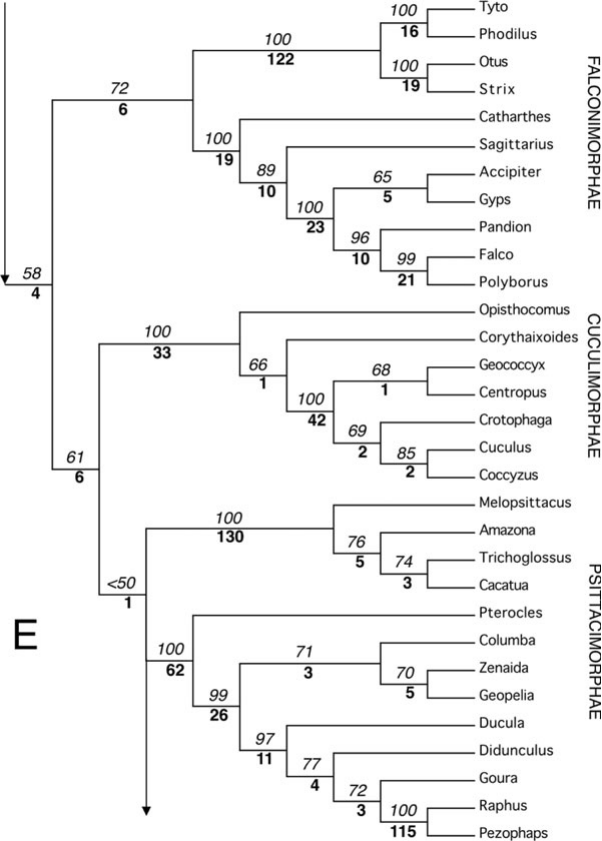
Detailed segment of strict consensus tree of all MPTs recovered in present study. Part E. Neornithes: Falconiformes, Strigiformes, Cuculiformes and Psittaciformes. Nodes are labelled above by percentages of bootstrapped replicates in which node was retained (italics), and below by Bremer support indices (bold type).

**Figure 17:**
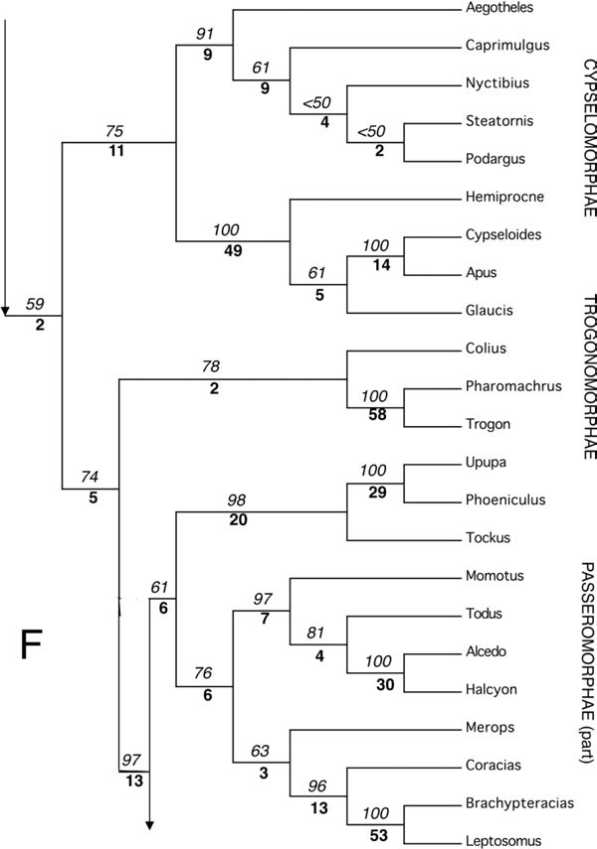
Detailed segment of strict consensus tree of all MPTs recovered in present study. Part F. Neornithes: Columbiformes, Caprimulgiformes, Apodiformes, Coliiformes, Trogoniformes and Coraciiformes. Nodes are labelled above by percentages of bootstrapped replicates in which node was retained (italics), and below by Bremer support indices (bold type).

Raptors or birds of prey – comprising the diurnal Falconiformes and (principally) nocturnal Strigiformes – share a primary reliance on carnivory, by scavenging or capture of prey and associated functional commonalities. The sister-relationship of these raptorial orders inferred herein (Figs 10, 16) and by Mayr *et al*. (2003) has been the subject of suspicion based on phenetic tallies of differences (Gadow, 1893; Beddard, 1898) and speculations concerning convergences and raptorial specializations (Sibley & Ahlquist, 1972; Cracraft, 1981). However, these orders differ in many respects and manifest substantial diversity within orders, conditions as suggestive of comparatively ancient divergence of sister-groups sharing general raptorial lifestyles and independent (order- and family-specific) morphological refinements. This clade is first in a sequence of four – the birds of prey, *Opisthocomus*, Cuculiformes, Psittaciformes and Columbiformes – that are sequential sister-groups of remaining Neornithes. Although all of these orders were robust with respect to individual monophyly, the four highest-order branches supporting these orders were not (Figs 10, 15–17), rendering the branching sequence provisional.

In addition to suspicions of convergence, several concerns may be seen as opposing the phylogeny inferred herein: (i) an alternative interordinal hypothesis that presumes the Strigiformes to be most closely related to the non-raptorial but similarly nocturnal Caprimulgiformes; (ii) an hypothesis that holds the New World vultures (Cathartidae) to be more closely related to the Ciconiidae than to typical birds of prey; and (iii) several counterproposals concerning certain families and genera of Falconiformes, notably positions of the terrestrially specialized secretary-bird (*Sagittarius serpentarius*), the piscivorous ospreys (*Pandion haliaetus*), and the distinctive Falconidae relative to other diurnal raptors.

### Falconiformes

In the present analysis, however, Cathartidae was resolved as the sister-group of other Falconiformes – an inference considered ‘probable’ by Sibley & Ahlquist (1972) – and the Sagittariidae was sister-group of the order exclusive of the Cathartidae. The Falconiformes, exclusive of the foregoing two families, comprised a pair of sister-clades: (i) the Accipitridae, including Old World vultures (e.g. *Gyps*), and (ii) a clade comprising the Pandionidae and its sister-group the Falconidae, the latter including the caracaras (Fig. 16).

Jollie (1976, 1977a, b, c) comparatively surveyed morphological characters of the Falconiformes in a monograph largely limited to anatomical phenetics and influenced by suspicions of functional convergence. Exclusive of primarily syringeal evidence (Griffiths, 1994), the only phylogenetic study of diurnal raptors based on morphological characters remains that by Holdaway (1994). Unfortunately, most studies treat most families within the Falconiformes (as construed herein) in only limited capacitiy or secondary focus, e.g. as outgroups for the Falconidae (Griffiths, 1994, 1999; Haring *et al.*, 2001; Griffiths *et al.*, 2004), or in treatments of other phylogenetic issues within the Accipitridae (Seibold & Helbig, 1996; Helbig *et al.*, 2005; Lerner & Mindell, 2005). Cytotaxonomy appears to possess signal, especially in the comparatively intensively studied Falconiformes, but even phenetic groupings of cytotaxonomy have defied interpretation (Ansari & Kaul, 1986). The recent sequence-based phylogeny proposed for the diurnal birds of prey (Lerner & Mindell, 2005) emphasized species-level relationships within the Accipitridae, and Fain & Houde (2004) failed to resolve relationships among the diurnal raptors. Lerner & Mindell (2005) differed from the present analysis in the placement of *Pandion* as more closely related to the Accipitridae than to the Falconidae or Phalcobaeninae. A sister-group relationship between Cathartidae and other Falconiformes, as inferred herein (Fig. 16), was recovered by Mayr & Clarke (2003), although the latter differed regarding the Strigiformes, Accipitridae and Falconidae.

Ligon (1967) tallied characters suggestive of a phenetic ‘affinity’ between the Cathartidae and Ciconiiformes. Evidently derived from studies by Gadow (1893), Beddard (1898) and Jollie (1953, 1976, 1977a, b, c), works that included Pelecaniformes and Procellariiformes as alternative candidates, the work by Ligon (1967) was a comparison of favoured features solely between the Cathartidae and selected representatives of Ardeidae, Ciconiidae and Accipitridae. Ligon (1967) did not consider polarities or include a formal analysis based on a broad array of characters, and most of the phenetic differences are not convincingly distinct; many features were cast in terms of antiquated typology (Cracraft *et al.*, 2003; Zusi & Livezey, 2006), such as the ‘palatal types’ of Huxley (1867). Nevertheless, this hypothesis found a receptive audience (Cracraft, 1972a; Cracraft & Rich, 1972; König, 1982; Rea, 1983; Emslie, 1988; Seibold & Helbig, 1995; Slikas, 1997; Lerner & Mindell, 2005), and it arguably is more popular than it is empirically robust.

Seibold & Helbig (1995) concluded that limited mtDNA sequence data supported a close relationship between the Cathartidae and storks. Subsequent analyses of the data used by Seibold & Helbig (1995) – revised and augmented by Hackett *et al*. (1995) and Avise & Nelson (1995) – largely were not comparable because of methodological differences. Avise, Nelson & Sibley (1994a) and Wink (1995) compiled weak molecular evidence to test the hypothesis, the results of which were equivocally consistent with the hypothesis of Ligon (1967). Analyses including these taxa during the following decade (Figs 1–10) failed to support the exclusion of the Cathartidae from Falconiformes *sensu stricto*, or associate the family with the Ciconiiformes.

### Strigiformes

The other substantive debate regarding birds of prey concerns the relative support for a sister-group relationship between: (i) diurnal and nocturnal raptors, or (ii) the similarly noctural Strigiformes and Caprimulgiformes (Hoff, 1966; Sibley & Ahlquist, 1972; Randi *et al.*, 1991; Wink & Heidrich, 1999). The current analysis strongly confirmed a sister-group relationship between the Strigiformes and the Falconiformes (Fig. 16), a union also supported by Cracraft (1988), Mayr & Clarke (2003) and Mayr *et al*. (2003). Recent molecular studies have placed the Strigiformes tenuously with a striking diversity of taxa, including the Psittacidae, Picidae and Rhamphastidae (Espinosa de los Monteros, 2000; Van Tuinen *et al.*, 2000). Fossils that exhibit generalized raptorial characters or those of both Strigiformes and Falconiformes also have been described (Mayr, 2000a, b, 2005b; Mayr & Daniels, 2001).

With respect to familial relationships within the Strigiformes, the present analysis reaffirmed a basal bifurcation between barn-owls (Tytonidae) and typical owls (Strigidae), with the former including *Phodilus* (Fig. 16). *Phodilus* (bay owl) has been considered of variable intermediacy to both strigiform families (Table 1), but most recent molecular data bearing on *Phodilus* (G. Barrowclough, pers. comm.) are consistent with the present findings (Fig. 16).

## Hoatzin, cuckoos, pigeons, parrots and allies

This group of medium-sized landbirds approximates part of the ‘Anomalogonatae’ of Garrod (1874) and Beddard (1898), largely synonymous with the earlier branches within the ‘higher landbird assemblage’ of Olson (1985). Clades informally included in this grade of modern landbirds are characterized by mutually exclusive apomorphies rendering many of the groups among the most readily recognized of birds. Members of this subterminal grade of avian orders have been the subject of numerous studies, but nonetheless a clear consensus regarding their interordinal affinities has failed to emerge (Bleiweiss, Kirsch & Lapointe, 1994; Bleiweiss, Kirsch & Shafi, 1995; Johansson *et al.*, 2001). As noted previously, these taxa –*Opisthocomus*, Cuculiformes, Psittaciformes and Columbiformes – traditionally were accorded ordinal rank, and were resolved here as a grade in which defining nodes achieved only marginal support. Accordingly, this series of clades conservatively can be considered to compose a tri-ordinal grade or corresponding polytomy that bridges the Cuculiformes with the Caprimulgiformes and Apodiformes. The latter ambiguity primarily relates to the failure to resolve the order of branching of the Psittaciformes relative to the Columbiformes (Fig. 17). Nevertheless, the orders branching from this grade were each strongly supported.

The Opisthocomidae – solely comprising the unusual hoatzin (*Opisthocomus hoazin*) – has been allied with Tinamidae, Galliformes, Cuculiformes, Columbidae, Pteroclidae, Rallidae, Otididae and Coliidae, among other higher-order groups (Table 1). Recent attempts to resolve the uncertainty of position of this monotypic lineage by molecular means have proven largely unsuccessful, principally by mutual contradiction or ambiquity of findings (Avise, Nelson & Sibley, 1994b; Hedges *et al.*, 1995; Marceliano, 1996; Sorenson *et al.*, 2003), and also because of contaminated sequence data (Avise & Nelson, 1995; Hackett *et al.*, 1995). A growing number of works are at least consistent with an affinity between *Opisthocomus* and the Cuculidae (Sibley & Ahlquist, 1972, 1990; Hughes & Baker, 1999), despite disputes regarding method and differences in taxonomic sampling. In the present analysis, *Opisthocomus* was placed as the sister-group of the Cuculiformes, the latter weakly including the Musophagidae (Veron, 1999) as sister-group to the Cuculidae (Table 2; Fig. 16).

Uncertainties of phylogenetic position and superficial plesiomorphy of *Opisthocomus* led some (e.g. Feduccia, 1980, 1996; Olson, 1985) to suggest that the taxon derives from the ‘roots’ of Neornithes. This proposal is consistent with a perception that the species descended from uniquely primitive ancestry, a view exemplified by its description as a ‘reptilian’ bird by Parker (1891), its use as the only neornithine explicitly figured with *Archaeopteryx* or non-avian Theropoda (Brodkorb, 1971a; Feduccia, Lingham-Soliar & Hinchliffe, 2005: fig. 26), and the much-publicised retention and use of weakly functional ungues alulares in the genus prior to fledging (Shufeldt, 1918). In actuality, such ‘wing claws’ are retained by members of many modern avian orders in variably vestigial states (Livezey & Zusi, 2006). Accordingly, morphological and molecular evidence for the purported plesiomorphy of *Opisthocomus* is ambiguous at best: most studies place the genus as closely related to the Cuculiformes (Hughes & Baker, 1999; present study), whereas a few analyses suggest a more distant relationship (Mayr *et al.*, 2003; Mayr, 2005b).

Various other studies, most with only marginal taxonomic sampling, have inferred a sister-group relationship between *Opisthocomus* and the Cariamidae (Mayr & Clarke, 2003; Mayr, 2005c) or inclusion within an eclectic assemblage defying plausible explanation in light of other findings (Fain & Houde, 2004). The unique alimentary features of *Opisthocomus*, notably refinements for herbivorous or ruminant digestion (Dominguez-Bello, Ruiz & Michelangeli, 1993; Kornegay, Schilling & Wilson, 1994), are of little phylogenetic significance as they are autapomorphic among Neornithes. However, the lysozymes associated with fermentation by Kornegay *et al*. (1994, 2003) suggest *Opisthocomus* to be more similar to *Columba* than *Gallus*.

Phylogenetic studies of the Cuculidae *per se* are surprisingly few, but include taxonomically inclusive attempts at morphological and ethological insights (Seibel, 1988; Hughes, 1996, 2000; Posso & Donatelli, 2001) as well as a molecular exploration (Sorenson & Payne, 2003). Berger (1960) compiled characters distinguishing the Cuclidae from the Musophagidae, many of which show homoplasy at wider scales of comparison. The molecular study by Johnson *et al*. (2000), the primary focus of which were the Malagasy couas, resulted in a topology within the family broadly similar to that inferred herein, differences in sampling notwithstanding (Fig. 16).

### Pigeons and sandgrouse

The Columbidae traditionally are recognized as monophyletic, whereas the interordinal position of the Columbiformes remains a primary point of dispute. The incompletely resolved position inferred here (Fig. 17): (i) compares reasonably well with the semi-speculative tree by Cracraft (1988); (ii) accords acceptably with the poorly resolved reconstructions by Mayr & Clarke (2003), Mayr *et al*. (2003) and Mayr (2005c); and (iii) is only weakly congruent with the placements by Van Tuinen *et al*. (2000) and Fain & Houde (2004). The fossil record of the Columbidae from the Palaeogene is poor, and described as non-existent by Mayr (2005a). Sampling of the Columbidae was comparatively intense in the present study so as to affirm the monophyly of such a diverse family and to expand the thoroughness of placements of the extinct ‘raphids’*Raphus* and *Pezophaps* (Livezey, 1993).

The present analysis indicated monophyly of flightless *Raphus cucullatus* and *Pezophaps solitaria*, one of the principal hypotheses proposed for the ‘raphids’ (Livezey, 1993). *Goura* and *Didunculus*, historically speculated to be sister-genera, were placed as paraphyletic to the raphids (Fig. 17). These inferences general agree with those by Shapiro *et al*. (2002: fig. 1) and Johnson & Clayton (2000a, b), and revealed generic partitions within the Columbidae in considerable agreement with the present work. The Pteroclidae (sand-grouse) have been the topic of study for more than a century (Gadow, 1882; Shufeldt, 1901c; Stegmann, 1957, 1959; Fjeldså, 1976). The pteroclids were placed herein as the sister-group of the Columbidae – a view favoured by the majority over an hypothesized alliance with the Charadriiformes (Sibley & Ahlquist, 1972, 1990).

### Parrots and allies

The primary mystery of this unique order is its interordinal position, a debate clearly manifested by the myriad groupings inferred for it in phylogenetic works during the last two decades. Monophyly of the Psittaciformes, not amenable to testing with the few exemplars included here, has been assumed (Smith, 1975) or affirmed by diverse morphological (Sibley & Ahlquist, 1972, 1990) and molecular means (Ovenden *et al.*, 1987; Christidis *et al.*, 1991; Leeton *et al.*, 1994; Miyaki *et al.*, 1998; Eberhard, Wright & Bermingham, 2001; Eberhard & Bermingham, 2001, 2004; Groombridge *et al.*, 2004; Russello & Amato, 2004; de Kloet & de Kloet, 2005; Ribas *et al.*, 2005; Tavares *et al.*, 2006). Higher-order relationships are less clear, and the order has been allied with: (i) groups comprising sufficient diversities of neognathous taxa as to establish little progress (Sibley & Ahlquist, 1990; Fain & Houde, 2004); (ii) Trogonidae and/or Coliidae (Espinosa de los Monteros, 2000; Mayr, 2000b, 2005d, e; Mayr & Clarke, 2003); (iii) Picidae (Van Tuinen *et al.*, 2000); (iv) Coliidae and some Pici (Mayr *et al.*, 2003); and (v) Strigidae (Harrison *et al.*, 2004). Although the present analysis provides no single, well-supported and precise position for the order, the evidence compiled herein is consonant with a (perhaps deep) relationship between the Columbiformes and Psittaciformes. This interordinal union was inferred by Burton (1974) in a study of *Didunculus*, and also confirmed by Sibley & Ahlquist (1972: 241) and Cracraft (1981).

## Goatsuckers, swifts and hummingbirds, mousebirds and trogons

### Overview

This heterogeneous group of moderate to small landbirds of controversial relationships – approximately synonymous with the ‘Coccygomorphae’ (Huxley, 1867), the Anomalogonatae (Garrod, 1874; Beddard, 1898), or part of the ‘ “higher” landbird assemblage’ (Olson, 1985) – includes some of the most specialized and distinctive of modern birds. Unlike the foregoing groups, most higher-order nodes within this assemblage – i.e. those structuring relationships among orders – are robustly resolved (Fig. 17). Essential findings herein were monophyly of the traditional Caprimulgiformes (including Aegothelidae), and monophyly of its sister-group Apodiformes. The Apodiformes comprised the crested-swifts (Hemiprocnidae) as sister-group to a clade comprising the mutually monophyletic typical swifts (Apodidae) and hummingbirds (Trochilidae).

### Caprimulgiformes

A minority of works failed to recover monophyly of the Caprimulgiformes, either by unresolved polytomy (Johansson *et al.*, 2001), variably constituted paraphyly to Trogonidae or Apodiformes (Mayr & Clarke, 2003; Mayr *et al.*, 2003; Mayr, 2005f; Barrowclough, Groth & Mertz, 2006), or uniquely proposed alternative alliances with the Accipitridae and Sulidae (Van Tuinen *et al.*, 2000), Sagittariidae (Mindell *et al.*, 1997), or Mesitornithidae (Fain & Houde, 2004). The present phylogeny affirms monophyly of the traditional Caprimulgiformes, although recent analyses suggest that the present study may not have been adequate to capture all ‘family-level’ variation in the order by omission of *Batrachostomus* and *Eurostopodus* (Sibley *et al.*, 1988; Mariaux & Braun, 1996). The moderately distant relationship between the nocturnal Caprimulgiformes and Strigiformes – considered closely related by some (Sibley *et al.*, 1988) – was favoured herein, a finding consistent with the hypothesis that at least the ocular refinements for nocturnality in these two orders are not homologous (Fidler, Kuhn & Gwinner, 2004). In this context it is noteworthy that another aspect of colour vision shows low consistency with avian phylogeny (Ödeen & Håstad, 2003). Support for groups within the Caprimulgiformes in the present work was marginal at best, and for practical purposes might be considered to be a polytomy of the included families. Palaeontological proposals suggest that fossil members of the Caprimulgiformes (and certain other groups) currently endemic to the southern hemisphere previously extended to the Palearctic (Olson, 1987; Mayr, 1999a, b, 2002a, b, 2005b, f).

Fidler *et al*. (2004) also presented equivocal evidence that the owlet-frogmouths (Aegothelidae) are not members of the Caprimulgiformes, as traditionally classified, a proposal augmented by some morphological evidence (Mayr 2002a, b) and DNA sequences (Barrowclough *et al.*, 2006). The latter studies led to marginally supported transfers of the Aegothelidae – herein inferred to be the sister-family of other Caprimulgiformes – to an alternative position as sister-group of the Apodiformes, a reasonably economical concession from global parsimony using the present data set (Table 3). Although the position of the Aegothelidae remains uncertain, mtDNA sequence data are consistent with the monophyly of this family (Dumbacher, Thane & Fleischer, 2003). A complete picture of caprimulgiform phylogeny must await comprehensive integration of putative fossil members (Olson, 1987; Mayr, 1999a, 2002a, b, 2005b, f).

The oilbird (*Steatornis caripensis*) – a nocturnal, cavernicolous frugivore – is one of the most challenging of avian genera with respect to phylogenetic position, irrespective of method. Recent analyses have differed regarding even the ordinal placement of this taxon, traditionally assigned to a monotypic family (Garrod, 1873b; Mariaux & Braun, 1996; Livezey & Zusi, 2001; Mayr, 2003b; Barrowclough *et al.*, 2006). The present work tentatively resolved *Steatornis* to be a highly apomorphic member of the Caprimulgiformes (Fig. 17).

### Apodiformes

This order is monophyletic and, as traditionally construed, comprises the highly derived crested-swifts, swifts, swiftlets and hummingbirds. The interfamilial relationships inferred here (Fig. 17) – Hemiprocnidae (crested-swifts) as sister-group to a clade comprising the mutually monophyletic Apodidae (swifts) and Trochilidae (hummingbirds) – have received growing support from other works (e.g. Chubb, 2004b) in tabling the largely antiquated contention that the hummingbirds were closely related to the Passeriformes and related variants of this hypothesis (Table 1). Departures from the present hypothesis included that of monophyly of the Hemiprocnidae and Apodidae (Chubb, 2004b). The molecular phenetics of Sibley & Ahlquist (1990), including where re-analysed (Harshman, 1994b) or augmented (Bleiweiss *et al.*, 1994, 1995), also differed by resolving the Trochilidae as phenetic ‘sister-group’ to all other Apodiformes, prompting the former to be ordinally distinguished as Trochiliformes. The speciose hummingbirds (Trochilidae) achieved phylogenetic diversity in concert with the related apodids (Mayr, 2003c, 2004d, 2005g), a radiation second only to passeriforms in scale (Bleiweiss, Kirsch & Matheus, 1997; Bleiweiss, 1998a, b, c; García-Moreno *et al.*, 2006), and evolved a diversity of concomitant apomorphies, some of which overcame unique locomotory challenges (e.g. Altshuler, Dudley & Ellington, 2004). Phylogenetic study of the swifts (Apodidae) by palaeontological (Mayr, 2001a, 2003c, 2005g) and molecular means (Dumbacher *et al.*, 2003; Chubb, 2004b; Thomassen *et al.*, 2005), resolved a fundamental dichotomy between taxa possessing capacities for echolocation (Thomassen *et al.*, 2003).

### Coliiformes and Trogoniformes

Modest support was afforded herein to a sister-group relationship between the Trogoniformes and Coliiformes (Fig. 17). Both orders have proven challenging to place among other avian orders (Garrod, 1876; Forbes, 1881), but monophyly of these orders has not be questioned (Espinosa de los Monteros, 1998, 2000). The present analysis confirms earlier anatomical (Verheyen, 1956b) and molecular analyses (Espinosa de los Monteros, 1998, 2000), in which this couplet also was inferred in turn to be closely related to Coraciiformes (Berman & Raikow, 1982). Johansson & Ericson (2004) conceded that appropriate outgroups for rooting analyses of these orders was problematic, a problem that led Moyle (2005) to employ a suite of outgroups. Among the more notable expansions of palaeodistributional limits have pertained to the Trogoniformes (Mayr, 1998a, 2001b) and Coliiformes (Mayr, 2000b, 2001a), although diagnostic evidence was poor by general neontological standards.

This couplet raises the possibility of artefactual grouping by way of long-branch attraction (Lyons-Weiler & Hoelzer, 1997; Wilson, 1999; Bergsten, 2005). Examination of ranges of terminal branch lengths compiled in the MPTs –*Colius* (84–133) and Trogonidae (10–17), with the branch subtending clade having a range of lengths 36–97 – suggests that the Trogonidae are not obviously vulnerable to an artefactual grouping. This judgement is supported further by the inclusion of a number of multistate, supportive characters (Wägele, 1996).

## Coraciiform, piciform, and passeriform birds

### Overview

Long recognized as a speciose, diverse and widespread group, historical disagreements pertaining to these orders have turned on familial memberships (e.g. Trogonidae) and delimitation of orders within the assemblage (Lowe, 1948; Wetmore, 1960; Sibley & Ahlquist, 1972: 241). Predictably, some subgroups manifested intermediate suites of characters and have proven least tractable (Burton 1984: fig. 32); the latter have been addressed most pointedly, perhaps, in palaeontological diagnoses (Ballmann, 1979; Mayr 1998b, c; Mayr & Daniels, 2001). Also, where data are less numerous, a common alternative to monophyly of the Coraciiformes or Piciformes is resolution of the two as sequential sister-groups (paraphyletic) to the Passeriformes. Although monophyly of the tri-ordinal assemblage was substantiated here (Figs 17, 18), the analysis revealed several alternative arrangements among the three orders, represented by the polytomy in the strict consensus tree of MPTs (Figs 17, 18).

### Coraciiformes

Monophyly of the families traditionally included within the Coraciiformes has been a point of disagreement for almost a century (Murie, 1872b, c, 1873; Lowe, 1948; Sibley & Ahlquist, 1972, 1990), and persists as a palaeontological challenge (Mayr, 2000d, 2005h, i; Mayr & Mourer-Chauviré, 2003; Mayr *et al.*, 2003). This state of affairs has been prolonged by poor representation of the order in many recent family-level, multi-ordinal analyses (e.g. Espinosa de los Monteros, 2000; Van Tuinen *et al.*, 2000), with notable exceptions including the analyses by Johansson *et al*. (2001) and Kirchman *et al*. (2001). Taxonomically narrow analyses include morphological works by Cracraft (1971b), Burton (1974) and Maurer & Raikow (1981), and the molecular phenetics of Sibley & Ahlquist (1990) and Bleiweiss *et al*. (1994).

The Coraciiformes were found herein to be a monophyletic member of a trichotomy that included the Piciformes and Passeriformes (Figs 10, 11, 17, 18), but the magnitude of support for monophyly of the Coraciiformes was only moderate, and generally was exceeded by that for included nodes. The ordinal work by Maurer & Raikow (1981) proved most relevant in this context, but conclusions of the two analyses differed considerably. Evidently, restriction of the outgroups and characters included in the analysis by Maurer & Raikow (1981) resulted in contradictory findings symptomatic of diminished signal, e.g. inversions of taxa within the ingroup and inclusion of Trogonidae within the ingroup.

The very distinctive hornbills (Bucerotidae), together with a sister-group comprising the Upupidae and Phoeniculidae, were situated as the sister-group of other coraciiforms (Fig. 18). This group was considered a separate order allied to other Coraciiformes by Burton (1984) and Kemp (1995). Manegold (2005), however, inferred the Coraciiformes to be polyphyletic and comprising: (i) the ‘Bucerotes’ (Upupidae, Phoeniculidae and Bucerotidae) as sister-group to the mutually monophyletic Piciformes (including Galbulae) and Passeriformes; (ii) the ‘Alcediniformes’ (all other members of the traditional order not included elsewhere); and (iii) *Leptosomus*, excluded from the Coraciiformes and of indeterminate ordinal relationship.

The remaining members fell into two sister-groups: one of these comprised the Motmotidae and its sister-group comprising the Todidae and Alcedinidae, the Todidae of uncontested monophyly (Overton & Rhoads, 2004), and the Alcedinidae monophyletic but perhaps comprising two or more distinct subgroups (Fry, 1980; Marks & Willard, 2005). The remaining member of this pair of sister-groups comprised the sequential sister-groups of Meropidae, Coraciidae, Brachypteraciidae and Leptosomatidae (Fig. 17). The Motmotidae were inferred herein to be the sister-group of a clade comprising the Todidae and Alcedinidae (Moyle, 2006). However, some ‘intermediacy’ of morphological and molecular characters of the tody-motmot (*Hylomanes*) and the Todidae suggests possible paraphyly of the Motmotidae (as traditionally constituted) or the Todidae (Overton & Rhoads, 2004). The Meropidae (bee-eaters), of established monophyly (Fry, 1984; Burt, 2004), were inferred here to be the sister-group to remaining Coraciiformes (Fig. 17), the latter known in the vernacular as ‘rollers’. As detailed above, morphological assessments of the memberships and positions of these families differ significantly (Manegold, 2005).

### Piciformes

The Galbulidae and Bucconidae were inferred herein to be sister-groups, and together as forming the sister-group of other Piciformes. The remaining Piciformes in this analysis comprised two sister-groups (Fig. 18), each of which comprised two, provisionally monophyletic families: (i) Capitonidae (Moyle, 2004) and Rhamphastidae (Eberhard & Bermingham, 2001; Wechstein, 2005); and (ii) Indicatoridae and Picidae (Prychitko & Moore, 1997; DeFilippis & Moore, 2000; Weibel & Moore, 2002a, b). Support for neither of the latter clades was strong, approximating only 50% bootstrap support (Fig. 18). This arrangement is consistent with much of the classification proposed by Burton (1984). One point of current debate is the possible paraphyly of the Capitonidae, in which member taxa represent successive sister-groups to the (monophyletic) Ramphastidae (e.g. Prum, 1988; Sibley *et al.*, 1988; Lanyon & Hall, 1994; Barker & Lanyon, 2000). Unfortunately, despite comparative richness of the record, fossil members of these groups have provided few insights into the phylogeny of modern piciforms (Mayr, 2001c, d, 2004e, 2005h, i).

Monophyly of the Piciformes, most often challenged regarding membership of the Galbulae, has been controversial – e.g. Olson (1983a), Raikow & Cracraft (1983), Lanyon & Zink (1987), Johansson & Ericson (2003) – despite comparatively detailed anatomical study (Burton, 1974) and related phylogenetic analyses (Simpson & Cracraft, 1981; Swierczewski & Raikow, 1981; Avise & Aquadro, 1987; Manegold, 2005). Most attempts to reconstruct the phylogenetics of the order have been variably inclusive with respect to included families and limited to molecular evidence (Webb & Moore, 2005; Wechstein, 2005; Benz, Robbins & Peterson, 2006), and resultant findings posed no serious contradictions to the inferences made here.

### Passeriformes

The Passeriformes are a dominant evolutionary component of the global avifauna, and the phylogeny of the order has figured prominently in terminological disputes regarding faunal ‘radiations’ (Barker *et al.*, 2004), ‘key innovations’ of evolutionary change (Raikow & Bledsoe, 2000; Olson, 2001) and ‘evolutionary success’ (Raikow, 1988). Current consensus by avian systematists holds the Passeriformes to be one of the most recently differentiated and apomorphic of lineages of modern birds, with a growing body of evidence for Gondwanan genesis (Ericson *et al.*, 2002a). However, analyses limited to the mitochondrial genome (Moore & deFilippis, 1997), the early mainstay of sequence analyses (Kessler & Avise, 1985; Ast *et al.*, 1997; Braun & Kimball, 2002), resulted in several studies in the placement of Passeriformes as the sister-group of most or all other Neoaves (Mindell *et al.*, 1997, 1999), a topological shift of exceptional magnitude and enormous evolutionary implications. This finding, mirrored by the phenetics depicted by Sibley & Ahlquist (1990) and very recent analyses based principally on mitogenomics (Pereira & Baker, 2006b; Slack *et al.*, 2006b), since has been attributed (Cracraft *et al.*, 2003, 2004) in subsequent works to (unavoidable) reliance on most closely related but nevertheless distant outgroups – e.g. Crocodylia, Testudines – which probably serve as unreliable sources of information on avian polarities. This circumstance, compounded by weak taxonomic sampling or shortcomings of mitochondrial data for reconstruction of deep nodes (e.g. Mindell *et al.*, 1996; Tsaousis *et al.*, 2005), necessitate caution in corresponding inferences. Fortunately, with respect to genomes analysed, principal differences reduce to rotations of three or four variably nested nodes (Johnson, 2001: fig. 3).

As for monophyly and composition of the Passeriformes (Beecher, 1953; Mayr, 1958; Olson, 1971; Feduccia, 1973, 1977c; Brom, 1990), the present analysis was necessarily limited to selected genera and families of this enormous group, as were the few previous morphologically based phylogenies of the group (Raikow, 1982, 1994a, b). A number of passeriform subgroups, mostly at comparatively low taxonomic levels, appear to have undergone cladogenesis sufficiently recently to reflect vicariance related to recent glaciations and current continental patterns (Edwards & Wilson, 1990), but controversy regarding this tempo persists (Klicka & Zink, 1997; Johnson & Cicero, 2004; Zink & Klicka, 2006). The meagre palaeontological evidence available indicates an origin of the Passeriformes to be no later than the early Eocene (Boles, 1995, 1997; Barker *et al.*, 2004; Mayr & Manegold, 2004).

The present analysis substantiated the interordinal position and monophyly of representatives of major subgroups of the Passeriformes (Fig. 18). Within the narrow taxonomic sample analysed herein (cf. Sibley, 1974; Cracraft, 1992a; Helm-Bychowski & Cracraft, 1993; Nunn & Cracraft, 1996; Barker *et al.*, 2002; Irestedt *et al.*, 2002), *Menura* was resolved as the sister-group of other members of the order, i.e. a member of the non-passerines (Fig. 18). *Menura* typically is situated crownward of basal *Acanthisitta* (but see Gadow, 1893; Ames, 1971) and included among the basal oscine passerines (Sibley & Ahlquist, 1970; Sibley, 1974; but see Ericson *et al.*, 2002a, b, 2003b, 2006). This minimally exemplified and questionably resolved subgroup was inferred to be the sister-group of remaining passeriforms, first followed by a poorly represented grade of suboscine passerines (Tyrannides) – Tyrannidae and Pittidae (Prum, 1993). The Tyrannides in turn subtends the oscine passerines (Passerides), within which two major subgroups (Ericson *et al.*, 2006) were sparsely represented but arranged in accord with current consensus (Fig. 18). Barker *et al*. (2002) inferred the Ptilonorhynchidae (Stonor, 1938; Cracraft, 1992a; Nunn & Cracraft, 1996; Cracraft & Feinstein, 2000; Johansson *et al.*, 2001) to be the sister-group of the remaining passeriform taxa. Among other Passerides, the single exemplar of the Corvida (*Aphelocoma*) was the sister-group of the three representatives of the Passerida –*Bombycilla*, *Parus* and *Passer* (Ericson *et al.*, 2000; Ericson & Johansson, 2003). With the exception of the position of *Menura* in the present analysis, the broad subdivisions inferred here agree with the majority of other recent works (Edwards, Arctander & Wilson, 1991; Irestedt *et al.*, 2001; Ericson *et al.*, 2003b; Cracraft *et al.*, 2004; Spicer & Dunipace, 2004) and are consistent with palaeogeographical evidence for an Australasian origin for the oscines (Boles, 1995, 1997; Barker *et al.*, 2004).

## Branch lengths and evolutionary change

Morphological characters employed in cladistic analyses tend to be held to unrealistic standards, and to serve as sources of insights (not expected of molecular characters) beyond mere inference of phylogenetic relationships. For example, in some circles there is an expectation that, in addition to resolving phylogenetic relationships of multiple taxa, apomorphies supportive of nodes should make obvious functional sense (e.g. debates regarding aquatic lineages and possible convergences) and permit interpretation resembling lists of (semi)diagnostic characters for nested series of taxa. In some cases, particularly where taxonomic scale is low and a functional focus pertains, such patterns and trends are discernible. However, with increasing taxonomic scale, these are in the minority, and like DNA sequence data, such diagnostic transparency and functional interpretation is seldom attainable. Many subtle features possessed of phylogenetic signal may be structural artefacts of functionally neutral details of anatomy, historical accidents that prove variably reliable through the process of evolutionary modification with descent.

Nevertheless, quantification of evolutionary change is critical to estimates of rates and correlation of change among characters and related evolutionary topics (e.g. Omland, 1997a, b; Nunn & Stanley, 1998), and exploration of this aspect of reconstructions is intended to pre-empt misplaced expectations or distorted perspectives. The tempo and mode of morphological evolution and cladogenesis have held the interests of systematists for decades (Simpson, 1944; Cracraft, 1984), pre-dating the advent of molecular methods or assumptions made for them (e.g. uniform or ‘clock-like’ evolutionary rates). Antiquity of lineages provides opportunity, other parameters being equal, for increased expectation of evolutionary changes (probabilistic, not deterministic expectation), and where such lineages comprise only modest numbers of members – i.e. limited evolutionary opportunities for departures from uniformity or reversals within a given lineage – such change also tends to lead to comparatively direct diagnosticity of terminal lineages. Intuitive relevance of origins, ages, longevities of lineages and expectations of evolutionary divergence notwithstanding, these topics have been underserved by newly acquired empirical evidence. Van Tuinen *et al*. (2006: table 2) listed 14 avian families construed to show molecular variation significantly lower than that expected on the basis of current taxonomic status. Given present findings, however, this issue appears illusory, with virtually all taxa in question having early origins, including the Anhimidae (Anseriformes), Podicipedidae and Spheniscidae, three being members of the Pelecaniformes, and the remaining examples members of either the Ciconiiformes, the Gruiformes or the Charadriiformes.

Unlike molecular evolution, no strict assumptions or dependence on constant or uniform rates of change have been made for morphological characters. In the present analysis, branch lengths varied substantially depending on specific optimizations, and therefore comparisons of lengths, like the internodes in trees, were not restricted to unambiguous changes. Instead, central tendencies of branch lengths of MPTs were quantified by median lengths, and variation among optimizations by standard deviations and ranges of lengths recovered. For Neornithes, numbers of changes optimized as autapomorphies averaged 41 (SD = 33, range 4–186). By contrast, for single lineages, maximal lengths of terminal branches were: 186 for *Spheniscus*, 132 for *Phoenicopterus* and 131 for *Mesitornis*. Minimal lengths of terminal branches were: 4 for *Francolinus* and *Alectoris*. At higher phylogenetic scales (interordinal and interfamimlial), branch lengths had the following summary statistics: mean = 36, SD = 33, range 5–133. In general, then, terminal branch lengths were approximately 10% greater than those of the branches subtending them (i.e. deeper internodes). A pattern of short internodes has been inferred previously (Cracraft *et al.*, 2004), but the attribution of cause to realities of evolutionary intervals vs. diminished power of resolution remains contentious.

A survey of the minimal branch lengths included in the MPT revealed that branches among higher-order nodes were extraordinarily similar to associated terminal branches (latter being those subtending individual taxa) in means and variances of branch lengths. However, comparative numbers of the more critical diagnostic and supportive characters within the Neornithes revealed that character-based definitions of highest-order clades (corresponding to the most ancient of synapomorphies) were disappointingly low, whereas those for superorders and orders (Appendix 2) were comparatively robust and included suites of diagnostic character-states (Table 2). However, the correspondence among ‘raw’ branch lengths, statistics of nodal support and numbers of ‘diagnostic’ apomorphies generally was poor (Table 2), in agreement with the findings of Farris *et al*. (2001) and Wilkinson (2003).

## DISCUSSION

### Broad comparisons with prior studies

‘Survival of the fittest will decide which of the many competing theories [of avian phylogeny] will prevail. Only one can survive. Each revisor attempts to shorten the struggle by acting as a selective factor.’ (Stresemann, 1959: 269)

‘Where the root of the Neoaves goes, however, is highly uncertain and seems likely to remain a very difficult problem.’ (Stanley & Cracraft, 2002: 39)

#### Perspectives and findings

In the published record of phylogenetics, it has become virtually customary simply to generate phylogenetic hypotheses of varying consonance with little or no consideration of factors underlying divergent inferences (Figs 1–9). This tradition has led to a false sense of congruence among studies, especially among molecular systematists. We consider that it is incumbent upon authors to consider the points of disagreement as well as the most plausible underlying philosophical and empirical reasons for the differences. A reasonable degree of detail in such deliberations inevitably will include points of contention and opinion, and we hope that these will challenge the current ambience of consensus and invite constructive debate of these important issues. At the same time, however, it is logistically unfeasible that large-scale studies (e.g. the present work) be held to standards of character descriptions and illustrations in analytical works that are logistically realistic in more common, small-scale works. For example, in the present study, a conservative estimate of character-states eligible for illustration would approach 7000. Nonetheless, access to underlying data for all studies should be made practically available by alternative means, and include formal descriptions of characters as analysed, and essential figures and references to critical descriptive works (e.g. Livezey & Zusi, 2006).

#### Deep tradition and the ‘tapestry’

Broad affinities of long standing between avian orders – traditionally only implied to variable degrees by adjacency in linear classifications (Clark, 1901; Wetmore, 1930, 1960; Mayr & Amadon, 1951) – that were not supported by the present analysis were: (i) Galliformes as closely allied with Falconiformes; (ii) Gaviiformes, Podicipediformes and Sphenisciformes placed as the most basal of ‘Carinatae’; and (iii) a truly basal position of *Opisthocomus* among Neornithes. Although confidence in the ‘tapestry’ (e.g. Monroe, 1989) diminished markedly within a few years of publication, the proposals by Sibley & Ahlquist (1990) were ‘rewoven’ by Harshman (1994b), ‘dusted off’ by Mooers & Cotgreave (1994), and continue to be cited for justification and design of sequence-based analyses (e.g. Fain & Houde, 2004). Limited reverence for the tome by Sibley and Ahlquist (1990) lingers, most conspicuously in the non-systematic literature (e.g. Del Hoyo, Elliott & Sardgatal, 2001), principally because of its taxonomic scale and molecular basis (e.g. Chubb, 2004b; Fain & Houde, 2004).

Given the controversy and contradictory nature of the era, it is appropriate to compare our findings with the groups delimited by Sibley & Ahlquist (1990), bearing in mind that the present phylogenetic analysis is of limited comparability with the phenetics of DNA–DNA hybridization. First, despite the unprecedented number of taxa analysed, the earlier work was invalidated shortly after its appearance because of problems stemming from phenetic methodology, sparsity of the distance matrix, absence of a root and irreducibility of data-type, some deficiencies having been identified prior to its release (Cracraft, 1987, 1992b; Houde, 1987; Sarich, Schmid & Marks, 1989; Barrowclough, 1992; Lanyon, 1992; Mindell, 1992). Simplification of the reconstruction by Sibley & Ahlquist (1990: figs 354–356) to ordinal terminal taxa (Fig. 4) reveals the diagram to be continuously pectinate throughout most of the neognathous birds, and largely reflects ‘chaining’ of least dissimilar elements, an artefact common to some agglomerative algorithms. Cracraft *et al*. (2004) considered current knowledge of avian phylogeny to be of comparable irresolution.

A most peculiar aspect of the ‘tapestry’ is the reversal of mid-basal and apical higher-taxa – e.g. Piciformes and Passeriformes as sister-groups to the ‘Ciconiiformes’ (*sensu*Sibley & Ahlquist, 1990) and allies – a finding countered by the vast majority of other analyses (Cracraft & Mindell, 1989; Johansson *et al.*, 2001; Braun & Kimball, 2002; Edwards *et al.*, 2002; Paton *et al.*, 2002; Mayr & Clarke, 2003; Mayr *et al.*, 2003; Prychitko & Moore, 2003; Dyke & Van Tuinen, 2004; Harrison *et al.*, 2004; Poe & Chubb, 2004). This phenetic artefact undoubtedly contributed to the poor congruence of the present phylogenetic hypothesis with that by Sibley & Ahlquist (1990), in which only four higher-order groups – their Ratitae, Galloanserae and Procellarioidea, and monophyly of one currently contentious order (Caprimulgiformes) – showed broad agreement in both works. The present analysis strongly countered the polyphyly inferred by Sibley and Ahlquist (1990) for the Pelecaniformes and Columbiformes, and differed as well regarding paraphyly of the Coraciiformes and Cuculiformes, the alternative positions of Galliformes + Anseriformes, and the provisional placement of the Turniciformes (Fig. 4).

Sibley & Ahlquist (1972: 240–241) listed 34 summary inferences entitled ‘Probabilities and Possibilities’, presented under four levels of perceived likelihood. Of the conclusions listed, agreement (with minor qualifications) with the present analysis was achieved for: all eight (100%) of the ‘highly probable’ conclusions; seven of ten (70%) deemed ‘probable’; four of ten (40%) considered ‘possible’; and only two of six statements (33%) classified as ‘improbable’, essentially logical negations of views included among the ‘highly probable’.

#### Contemporary studies

Comparisons among most phylogenetic hypotheses are compromised by differential taxonomic sampling and nodes afforded only tenuous support. The present phylogenetic hypothesis, depicted to ordinal scale (Figs 10, 11), approximated the tree depicted by Cracraft (1988) most closely of prior works, issues of comparability notwithstanding. The present analysis, almost 20 years subsequent to that by Cracraft (1988), represents a return to the broad outlines of the latter, seminal work. Given the different scales of the two analyses in terms of taxa and characters, however, it is unreasonable to assume similarities to be the result of reliance on ‘the same characters’.

An increasing proportion of all studies confirm positions and monophyly of Palaeognathae, Galloanserimorphae and major subclades thereof. However, most molecular studies (e.g. Van Tuinen *et al.*, 2000, 2001; Paton *et al.*, 2002; Van Tuinen, 2002; Chubb, 2004a), as well as analyses based on combined data (Dyke & Van Tuinen, 2004), differed significantly with parts of the present hypothesis, especially those pertaining to the Pelecaniformes, Ciconiiformes, Podicipedidae, Opisthocomiformes, Cathartidae, Caprimulgiformes and Coraciiformes (Figs 12–18). There was considerable disagreement among recent molecular studies alone (e.g. Espinosa de los Monteros, 2000; Johansson *et al.*, 2001; Poe & Chubb, 2004), regardless of data analysed (Philippe *et al.*, 1996; Graur & Li, 2000), which reveals contrasts only between morphological and molecular inferences to be oversimplifications of modern study (e.g. Braun & Brumfield, 1998; Van Tuinen, 2002).

Comparisons with the limited number of other analyses (Figs 1–3) were virtually uniformative because palaeontological works have tended to emphasize narrow taxonomic groups considered likely to accommodate newly described or controversial taxa, and also to limit characters to those scoreable for the taxon or fossil of interest (e.g. Clarke *et al.*, 2005b), with some exceptions (e.g. Mayr & Clarke, 2003). Several provisional and ongoing reconstructions by Cracraft *et al*. (2004) were not considered here. A survey of comparable cladistic studies of morphological or molecular bases (cf. Cracraft, 2001; Mayr & Clarke, 2003; Cracraft *et al.*, 2004; Fain & Houde, 2004; Clarke *et al.*, 2005b) revealed that the present analysis achieved considerable agreement with most of the latter studies concerning the widely supported (com)positions of the Palaeognathae and Galloanserimorphae, and an allied clade dominated by marine and wading birds (Figs 10–18).

#### Adjudication of success

It is to be hoped that diverse approaches will converge empirically toward common analytical standards (Lake, 1997) and a solution for which acceptance is widespread and merited. However, there are no standards of accuracy against which phylogenetic analyses of natural lineages can be calibrated (i.e. known histories), and therefore the assessment of progress is elusive. Hypothetico-deductive empiricism may reveal critical characteristics of scientific hypotheses, but cannot provide ‘proof’ of a hypothesis (Helfenbein & DeSalle, 2005).

Given that proof of hypotheses or certain recognition of the single, true phylogeny is unattainable, the strongest support for a specific reconstruction (beyond intrinsic robustness) lies in common elements shared by other analyses – empirical (not popular) consensus. Such studies are most potent where performed independently using new data. Likelihood of correctness of molecular and morphological reconstructions cannot be judged a priori, especially across all classes of investigation. Such assessments are conditional on individual cases, and decisions based on consistency with prior analyses, degree of resolution (assuming bifurcations are the primary cladogenetic mode), size and diversity of data on which the hypothesis was based, and analytical properties of included characters. The relevance of statistics internal to single trees – e.g. robustness of nodes and consistency indices – to the likelihood of global accuracy is undecided (Benton, 2001).

Consequently, an important element of phylogenetic study is comparison of findings with the estimates of other investigators, especially comparisons of those aspects of trees that withstand variations in method or data base. However, against which topology or topologies does one compare specific findings? This quandary especially afflicts those disposed to a dichotomous view of morphological and molecular estimates of history. Provision of a sample of trees (Figs 1–9) was intended, in part, to emphasize the dilemma that faces investigators wishing to evaluate hypotheses comparatively. It appears that until some kind of genuine consensus is achieved, systematists are compelled to pit their findings against a plethora of other, marginally comparable works.

### Molecular systematics: competitor or collaborator?

At present, molecular systematics is characterized both by the coexistence of general (if not unbridled) optimism (Van Tuinen, 2002) and by profound doubts regarding resolution of substantial segments of neornithine phylogeny (Poe & Chubb, 2004). Yet the current dominance of avian systematics by molecular methods is sufficiently profound as to lead some to consider palaeontology to be the sole justification for a continued role for morphology in systematics or to question its value altogether (e.g. Stevens, 2000; Scotland, Olmstead & Bennett, 2003; Jenner, 2004a, b). Nevertheless, historical signal from genes and their morphological products offer a potentially fruitful synergy (Jenner 2004a: 340), one that exceeds the use of morphology for placements of fossils.

An unfortunate pattern has emerged in molecular circles, however, in which perennial problems of avian systematics (Table 1) are attributed to the relative impotence or unreliability of morphological clues to phylogeny (e.g. Monroe, 1989; Sibley & Ahlquist, 1990; Givnish & Sytsma, 1997a, b; Sorenson *et al.*, 1999; Paton *et al.*, 2003; Paton & Baker, 2006), or as justification for merely mapping morphological characters a posteriori onto molecular trees (e.g. Gittleman *et al.*, 1996; Slikas, 1997; McCracken *et al.*, 1999; Van Tuinen, 2002; Huelsenbeck *et al.*, 2003). Therefore, it would be negligent to forego this opportunity to counter this perception explicitly (e.g. Shafer, Clark & Kraus, 1991; Hillis & Wiens, 2000; Marques & Gnaspini, 2001). We do not intend an assault on molecular methodology, but seek to refute persistent prejudices that afflict morphological phylogenetics (cf. Smith, 1998; Baker & Gatesy, 2002), to underline the distinctness between ease of application and reliability in phylogenetic methods, and to encourage objectivity in assessment of findings.

Perhaps the deficiency attributed most widely to morphological phylogenetics stems from suspicions of morphological convergence, concerns seldom empirically substantiated and to which molecular methods are widely assumed to be immune (Lockhart *et al.*, 1994; Goldring & Dean, 1998; Lee, 1997, 1999; Sorenson *et al.*, 1999; Yang & Bielawski, 2000). To date, assumptions of morphological convergence principally are made where convenient and are seldom reversed, with few exceptions (e.g. McCracken *et al.*, 1999; McCracken & Sorenson, 2005). However, verification of convergence in molecular data (Holmquist, Pearl & Jukes, 1983; Kornegay *et al.*, 1994; Philippe *et al.*, 1996) is increasingly frequent. For morphology, we hope that intuitive claims of convergence will be supplanted by phylogenetically framed analyses of refined morphological and functional data (Raikow, 1985b), especially those pertinent to the: pectoral limb (Middleton & Gatesy, 2000; Burness, Chardine & Darveau, 2005); pelvic limb (Gatesy, 1991; Gatesy & Biewiener, 1991; McKitrick, 1993; Patak & Baldwin, 1998; Carrano & Biewener, 1999; Abourachid, 2000, 2001; Abourachid & Renous, 2000; Hutchinson, 2001a, b, 2002; Zeffer & Norberg, 2003; Zeffer *et al*., 2003; Fujita, 2004); skull and associated musculature (Müller, 1961a, b, 1963; Weber, 1990, 1993; Zusi & Livezey, 2000; Bout, 1997; Meekangvan *et al.*, 2006); and general body form (Nudds & Rayner, 2006; Bybee, Lee & Lamm, 2006).

Studies based both on molecular and morphological phylogenetics (Figs 1–9) manifest substantial disagreement both within and between schools (Patterson *et al.*, 1993), and remain comparable in resolution and support, with disputes often conjectural in nature. Both classes of data present substantial challenges of homology (further below), and those that face molecular systematists (Wheeler *et al.*, 1995; Philippe *et al.*, 1996; Phillips *et al.*, 2000; Jenner, 2004a, b; Wiens, 2004) are remarkably similar to those afflicting morphological phylogeneticists. Problems of homology in molecular applications, principally related to ‘gaps’, indels, and their implications for serial homology and sequence alignment (Redelings & Suchard, 2005), include: bias in substitution and codons (Collins, Wimberger & Naylor, 1994; Kreitman & Antezana, 2000); concerted evolution (Drouin & Moniz de Sá, 1995; Eberhard *et al.*, 2001); pseudogenes (Nielsen & Arctander, 2001); silent substitutions and undetected heterogeneity in rates of substitution (Wakeley, 1994; Simon *et al.*, 1996); selectively constrained evolutionary rates of repetitive DNA families (Chen *et al.*, 1991); homoplasy indirectly related to the four-state sampling universe of nucleotides (Wägele, 1995, 1996); and independence of molecular ‘characters’ (Zardoya *et al.*, 1998; Graur & Li, 2000; Felsenstein, 2004).

Similarly, subjectivities of sampling and analysis beset both morphologists and molecular systematists, including: sampling of genes (Zardoya & Meyer, 1996; Moore & deFilippis, 1997; Pollock *et al.*, 2002) and taxa (Bergsten, 2005); comparative weighting (García-Moreno, 2004); branch support (Felsenstein & Kishino, 1993; Suzuki, Glazko & Nei, 2002; Alfaro, Zoller & Lutzoni, 2003); and model selection (Mort *et al.*, 2000; Buckley, Simon & Chambers, 2001; Huelsenbeck *et al.*, 2002; Simmons *et al.*, 2004; Lee & Hugall, 2005; Pickett & Randle, 2005; Pickett *et al.*, 2005; Steel & Pickett, 2006). In addition, the critical distinction between ‘gene trees’ and ‘species trees’, which can differ significantly (Page & Charleston, 1997; Berglund-Sonnhammer *et al.*, 2006), may be overlooked or ignored (Pamilo & Nei, 1988; Doyle, 1992, 1996; Moore, 1995; Maddison, 1997; Page & Charleston, 1997; Thornton, 2002; Geeta, 2003).

Despite these considerable challenges, molecular systematics clearly holds great potential for resolution of many problems of avian systematics, particularly in the Passeriformes. An informal survey of the passeriform literature since 1990 revealed studies of diverse taxonomic scales: 11 subordinal, five superfamilial, 34 (sub)familial, 55 generic and 24 (super)specific. This considerable success notwithstanding, largely unexplored is the potential of enterprises jointly including molecular characters of sequence and higher-order genomic structure (Kadi *et al.*, 1993; Delport, Ferguson & Bloomer, 2002; Prychitko & Moore, 2003; Slack *et al.*, 2003; de Kloet & de Kloet, 2003, 2005; Edwards, Jennings & Shedlock, 2005), the latter ignored at considerable peril (Winnepennincks & Backeljau, 1996). Together with morphological data of fossil and modern taxa, such molecular diversity appears to be essential for progress at many scales of avian phylogeny (Graybeal, 1994; Edwards *et al.*, 2002; Harrison *et al.*, 2004; Simon *et al.*, 2004).

#### Appeal of the novel and unexpected

Apparent departures from taxonomic groups supported throughout much of the cladistic or molecular eras have been frequent during recent years (Cracraft *et al.*, 2003, 2004). Fain & Houde (2004: 2570) proposed the entertainment of a number of counter-intuitive and weakly supported groupings in their analysis, in the spirit of freeing systematists from being ‘… guided by preconceptions of relationships.’ The latter appeal for objectivity is unquestionably laudable, but the fact that the proposed groups were merely novel does not constitute affirmation of any kind. Similarly, Pons, Hassanin & Crochet (2005: 686) stated that their study: ‘… identifies *for the first time* some sister relationships that *had never been suggested before*.’[emphasis added]. Although many traditionally recognized higher-order groups deserve formal analysis, novelty of resultant proposals is irrelevant to these endeavours. Realization of this potential primarily turns on two issues of modern systematics – rigorous and nomenclaturally transparent analyses that bridge subdisciplines (beyond the recent penchant to use fossils in molecular phylogenies for estimates of evolutionary rates), and empirically justified views and integration of morphological evidence in an era of increasing reliance on molecular inference.

### Morphological homology–ontogeny, function and phylogeny

#### Insights from avian phylogeny

Hope for success lies in a pluralistic approach to evidence (Cracraft *et al.*, 2004). This goal, in turn, is conditional on the surrender of prejudice and a common concept of homology. Ornithological systematics is replete with assumptions, assertions and inferences concerning homology and its role in the recognition of characters and evolutionary patterns (Freudenstein, 2005). In practice, variously defined ‘sameness’ is the basis for pre-analytical (a priori) assessments of homology in phylogenetics (Wake, 1999), but resultant phylogenies provide the historical framework within which homology is confirmed a posteriori (Haszprunar, 1998). However, non-historical criteria have been attached to the concept of homology virtually since its theoretical origins, of which ontogeny and function were perhaps the most common. Accordingly, alternative perceptions have influenced avian phylogenetics virtually throughout its history, particularly regarding homology and paedomorphic characters, homoplasy and convergence, the concept of *Grundplans* (e.g. Weber, 1990), and implications of ontogeny and genetics for homology of characters.

#### Phylogenetics and homology

Homology is synapomorphy at some phylogenetic level (Nelson, 1994), and is defined a priori as ‘similarity due to common descent’ (West-Eberhard, 2003: 485). Hall (2003) equated homology with identity (despite change) made evident by phylogeny: homology, reversals, rudimentia, vestigia, atavisms and parallelisms. Considerations of parallelism and convergence for Aves involve aspects of cranial structures (Starck, 1969) among outgroups (Carroll, 1988, 1997; Unwin, 1993; Brochu, 2001). Examples of atavism are few, but include the recurrence of a plesiomorphic pelvic muscle among Paradiesidae (Raikow, Borecky & Berman, 1980). Strong examples of morphological parallelism in birds involve the evolutionary loss of flight by flightless Rallidae (Livezey, 2003b).

#### Similarity and homology

Homology is conditional on essential, potentially mutable ‘sameness’ of a character manifesting continuity of descent within a phylogenetic hypothesis, whereas common function and ontogeny are not conditions thereof (Hall, 1994, 2003; Wake, 1999). Müller & Newman (1999) advocated secondary qualities of generation, integration and autonomy of a structure for the status of homology to be conferred, nuances herein considered components of the essential ‘sameness’, if considered at all. Variants of characters recognized in a phylogenetic context (putative homologues) and manifesting modification with descent – affected by any of a number of mechanisms of evolutionary change (selection, drift, mutation, ontogenetic deviation) – are treated as ‘states’ of a given character here.

#### Ontogeny and homology

The ontogenetic mechanisms that produce homologous states of a character are of considerable evolutionary interest and may prove critical in particular cases of diagnosis (Wagner, 1989), but do not qualify as criteria of homology of terminal features *per se* (Cracraft, 1967b; Hall, 2003). Genetics of ontogeny, however, can provide unique insights into the bases of likely homologues, e.g. odontogenesis and the edentuly of modern birds (Chen *et al.*, 2000; Mitsiadis, Caton & Cobourne, 2006).

A synthetic view of homology holds that respective developmental stages of members of lineages, interpreted hierarchically within a phylogenetic framework, are each potential homologues capable of partitioned evolutionary patterns (Abouheif, 1997). Thus, homologues are defined within each developmental stage of each character (Hall, 2003), e.g., genes, developmental processes and stages thereof. However, judgements of homology based on ontogenetic processes are mistaken extensions of identity of descent across quasi-autonomous developmental modules (Rieppel, 1992, 1994; Wagner, 1994; Santini & Stellwag, 2002; Arthur, 2004). Traditional assertions that homologues must share genetic foundations represent similar overextensions of historical identity (Hall, 2003). Variants, including asymmetry, of a terminal character evolved during phylogenetic descent by means of developmental change are homologues of the given character, and variation in the ontogenetic mechanisms behind evolution of the character are not necessarily evidence of non-homology of the resultant states (Hall, 1994; Cooke, 2004). For practical considerations, predefinitive homologues are problematic for fossil birds as useful fossil embryos are rare (Elzanowski, 1981; Norell *et al.*, 1994).

Developmental sequences include potentially distinct components such as developmental cascades, changes in timing (heterochrony) and position (heterotopy), and frame shifts (Hall, 1984; McKinney *et al.*, 1990; Schulmeister & Wheeler, 2004). Where ontogenetic mechanisms *per se* are potential characters, the concept of modularity of development (Minelli, 1998; Raff & Raff, 2000) implies a delimitation of ontogenetic processes as characters in themselves. Of recent concern is the digital frame-shift within the digiti manus avium (Wagner & Gauthier, 1999), which counters the former embryological hypothesis of Hinchliffe (1985) that is still advocated by Burke & Feduccia (1997) and Feduccia (1999). Subsequent study has implicated *Hox* genes in such shifts (Chiu *et al.*, 2000; Vargas & Fallon, 2005a, b), although the proposal is not without controversy (Galis, Kundrát & Metz, 2005). The modularity of development permits the view of the hypothesis of Wagner & Gauthier (1999) as but one characterization of several plausible candidates based on embryological principles (Galis, van Alphen & Metz, 2002; Hamrick, 2002; Welten *et al.*, 2005).

Several other instances are variably conspicuous cases of heterochrony (McKinney *et al.*, 1990; Klingenberg, 1998; Livezey, 2003b) – e.g. shifts in general developmental trajectories of Megapodidae (Starck & Sutter, 2000) and that of the avian furcula (Hall, 2001). Perceptions regarding the diagnostic relevance of anatomical position with respect to homology vary (Zelditch & Fink, 1996), e.g. the partly positional argument of the ‘rostro-parasphenoid’ process as distinct from the traditionally defined processus basipterygoideus (Weber, 1993), typological paradigms (Richardson, Minelli & Coates, 1999), and the role of function (Elzanowski, 1977). Patterns imposed by altered proximodistal developmental axes of appendages (Richardson, Jeffrey & Tabin, 2004) or action of regulatory (e.g. *Hox*) genes (Galis, 1999; Telford, 2000) increasingly are recognized in changes among transitional and terminal (definitive) developmental homologues. To date, most references to heterochrony have emphasized paedomorphic characters, i.e. variants of homologues typical of juveniles of plesiomorphic relatives (Livezey, 1995a, 2003b; Fink, 1988), and include more than simple alteration of growth rates (Starck & Sutter, 2000). Instead of undermining homology, such instances of heterochrony provide potentially novel synapomorphies among paedomorphs (Cracraft, 1981; Raff *et al.*, 1990).

The law of Von Baer (Gould, 1977) – the biogenetic law – postulated that the order of developmental stages in an individual reflects the phylogenetic series of increasingly apomorphic states found in that lineage. In some cases, this series approximates the evolutionary changes leading to the terminal state (Gould, 1977), and may provide possible insights into polarities and transformation series (Kraus, 1978; Alberch, 1985; Shubin, 1994; Jeffrey *et al.*, 2002; Grant & Kluge, 2004; Schulmeister & Wheeler, 2004) – i.e. states consistent with the ‘ontogenetic criterion’ (Alberch, 1985; Meier, 1997; Mabee, 2000). Avian candidates for this criterion include the angulus coracoscapularis and mutliple unions of elements or *anlagen* of the definitive avian shoulder girdle (Livezey & Zusi, 2006).

#### Function, homology and convergence

Cladistic (parsimony) analysis often is charged with a disregard for functional implications and convergence of character states, causing systematists to mistake similar but independently derived features among distantly related taxa as homologous. Convergence without demonstrated phylogenetic influence, as well as naive historical examples – e.g. purported affinities of swifts and swallows, tabled decades ago (e.g. Shufeldt 1889b; Lowe, 1939; *contra*Van Tuinen, 2002) – do not merit consideration here. Bock (1967, 1979) and Homberger (1980) considered function to be a critical criterion of homology, the independent study of which being required prior to inclusion of the structure in question in a phylogenetic analysis. Notable examples of this paradigm concern specializations of the feeding apparatus of Coraciiformes (Rawal & Bhatt, 1974) and Picidae (Bock, 1999), or cranial refinements among Charadriidae (Kozlova, 1961). Hypotheses of homology between features require a phylogenetic framework, and mere similarity of function in two potential homologues fails to demonstrate or exclude homology or convergence.

Convergence frequently is invoked in the context of adaptation (Coddington, 1994) and, at least in ornithological tradition, by superficial comparisons of the structures among distantly related lineages that share function, e.g. pelvic limbs of pursuit divers (cormorants, mergansers, loons), forelimbs of wing-propelled divers (alcids, diving petrels, penguins), or bills of piscivores (herons, anhingas, kingfishers). It is notable, however, that phylogenetic relationships among these examples were not obfuscated herein by these analogous similiarities in light of the totality of characters analysed, and that the purported instances of convergence were limited to a minority of phylogenetically analysed characters.

Given that character-states are homologous variants of a particular character defined a priori by critical similarity and a posteriori by continuity of descent, considerations of function, although of evolutionary interest, are not directly germaine to homology or its diagnosis (Lauder, 1994). Function, and its possible relationship to form, constitute but one potential precondition of convergence – one component of homoplasy (Hall, 2003). Examples of homologues cited independently of ontogeny in birds include: the processus basipterygoideus (Elzanowski, 1977) and modifications for dorsoventral movements of the carpus (Vazquez, 1992). Given that homology, and therefore homoplasy, are diagnosed reliably only within a phylogenetic context, non-hierarchical assessments of homoplasy (Faith, 1989; Zeffer *et al.*, 2003) offer few if any insights.

Simpson (1944) discussed the differentiation of convergence from parallelism in closely related taxa under the term ‘parallel evolution’, and Bock (1963a) described it in the context of ‘evolutionary homodynamy’. Attribution of taxonomic groupings to convergence is conditional on: (i) the case for homology and plausible selection effecting changes (Fusco, 2001); (ii) reliability of phylogenetic analyses indicating disjunction of the disputed groups (Sommer, 1999); and (iii) the independence of phylogenetic reconstructions from sources of potential bias (Lee & Doughty, 1997). Two avian examples follow that might be taken by some to exemplify cases of ‘convergence’ of morphological characters leading to erroneous phylogenetic groups, exercises critical to assessing perceptions relative to empirical evidence (Wiens *et al.*, 2003).

*Ratites*. We found strong support for monophyly of ratites, a finding in agreement with current consensus. The prior hypothesis of polyphyly was confounded by a perspective of static continents, convergence (Cracraft, 1974a) and a phenetic emphasis on differences (McDowell, 1948; Starck, 1955; Lang, 1956; Romer, 1968; Storer, 1971a). Synapomorphies of ratites not reasonably related to flightlessness or giantism fail as cladistic support for polyphyly. Finally, advocates of convergence fail to propose an empirically supported, plausible alternative hypothesis of relationship(s) consistent with the morphological (and molecular) data.

In our analysis, no support was found to ally any ratites to other non-ratite taxa; by contrast, 75 characters were ‘diagnostic’ or ‘highly supportive’ of monophyly of ratites (Table 2). Of these, 30 referred to the pectoral girdle or wing. If these 30 characters are accepted to be homologous as coded, they would lend support to the monophyly of ratites and (by parsimony) their shared flightlessness. On the other hand, if analysis had indicated that these same characters were optimized parsimoniously as non-homologous (but not coded a priori as such, absent evidence), the inference would be consistent with the possibility that flightlessness evolved in parallel more than once within a palaeognathous clade. Neither the present phylogeny nor alternative scenarios provide conclusive evidence for hypotheses concerning relative sequence(s) in the evolution of flightlessness in ratites; evolutionary trends of this kind are best explored through optimizations of character-suites a posteriori on the phylogenetic hypothesis (Fig. 13).

Candidates for parallelism of potential phylogenetic influence include the synostosis scapulocoracoideum of ratites and its marked similarity with those of non-avian Theropoda (Feduccia, 1986), and convergent enlargement of the angulus coracoscapularis of flightless Neornithes (Livezey & Humphrey, 1986; Livezey, 1988, 1989a, b, c, 1990, 1992a, b, 1993, 1995a), especially in the ratites and Rallidae (Livezey, 2003b). Diagnosis of the scapulocoracoideum as atavism would hinge on the phylogenetic history of the feature. A similar challenge attends classification of other pectoral changes among ratites as plesiomorphy, synapomorphy, parallelism or convergence.

*Grebes and loons*. We inferred the loons and grebes to be sister taxa, with no comparable support for positioning either taxon more strongly elsewhere (Table 3). Of the 17 characters diagnostic or highly supportive of this relationship (Table 2), 11 are from the pelvic girdle and limb (Livezey & Zusi, 2006). Those who considered these taxa to be only distantly related typically espoused a certainty that the similarities of the hindlimb and pelvis were misleading convergences associated with foot-propelled diving (Storer, 1956: 426; Storer, 1971a: 5). Suspected convergence is not supported by the differences in the hindlimbs of loons and grebes in that such are at least as parsimoniously interpreted to be: (i) symplesiomorphies differentially lost or modified in the lineages following divergence; or (ii) autapomorphies acquired independently following divergence of the orders. Neither has been shown to be parsimoniously synapomorphic with one or more other avian orders (Fig. 14). It is noteworthy that proponents of an alliance between the grebes and flamingos are tolerant of multiple dissimilarities between the groups (Chubb, 2004a). Whatever the scenario, the support index for this couplet of orders (Table 2; Fig. 14) significantly counters a convergent history for these characters, and an alliance with either the Charadriomorphae or the Phoenicopteridae entailed substantial sacrifices in parsimony (Table 3).

### Palaeornithology: contrasting perspectives, common goals

#### Contrasts of ends and means

Until recently the fossil record for birds was marginalized with respect to formal phylogenetics, with most fossil taxa being fragmentary representatives or close relatives of modern groups. A spate of newly discovered fossils from the late Mesozoic has clarified greatly the theropod roots of birds. Despite consensus concerning the phylogenetic implications of new Mesozoic fossils and a number of shared goals, neontological and palaeontological schools often work at cross purposes. A former obstruction to unified analysis was a tradition of speculative evolutionary scenarios with strong palaeontological underpinnings, notably concerning evolutionary transitions and diversification (Olson, 1985; Feduccia, 1995, 2003; Chatterjee, 1997; Kardong & Zweers, 1997; Zweers & Vanden Berge, 1997a, b; Zweers *et al.*, 1997; Bleiweiss, 1998c; Feduccia *et al.*, 2005) and avifaunal ‘assemblages’ (Brodkorb, 1971a, 1976; Olson, 1985), that served as surrogates for ecological data not available for fossil lineages and past eras. A significant convergence in cladistic methods notwithstanding, it remains an unfortunate impediment that goals, expectations, nomenclature and assumptions of avian palaeontologists and neontologists (Cracraft, 1972b, 1974b, 1978, 1979, 1980) exist in largely parallel circles and have failed to realize a commonality of professional purpose. The most serious analytical challenges posed by avian fossils derive from missing data (Kearney, 2002; Kearney & Clark, 2003), which may affect the characters admitted for analysis.

#### Nomenclatural divergence, analytical corollaries

Issues of strict taxonomy aside, philosophical differences between the subdisciplines also involve long-standing perceptions of the diagnosibility of direct ancestry (e.g. Brodkorb, 1976; Olson, 1976). Palaeontological viewpoints regarding ancestral status of fossils also hold implications for nomenclature of fossil lineages (e.g. ‘stem-groups’) in phylogenetics (Benton, 2000), analytical validity of ‘ghost lineages’ (Norell, 1992), and the evolutionary significance of fossil ‘mosaics’ (Norell & Clarke, 2001; Dyke & Van Tuinen, 2004). A neontological perspective, however, considers fossils to differ from modern representatives solely by extinct status and quality of preservation, with many modern lineages representing more informative plesiomorphs of extant clades than any fossil member – e.g. anseriforms Anhimidae, *Anseranas* vs. fossil *Presbyornis* (Livezey, 1997a). Where adequately preserved for phylogenetic placement, fossils also may provide an estimate of minimal age of the group it represents, but this estimate is imprecise and subject to bias. Ironically, a misunderstanding of such estimation contributed to early arguments concerning ‘temporal incongruence’ and against a theropod origin for birds (e.g. Brochu & Norell, 2000, 2001).

Although the definitions of ‘stem’ and ‘crown’ groups are relatively simple (Meier & Richter, 1992), the former sharing conceptual roots with earlier terms of assumed or possible direct ancestry such as ‘plesions’ (Wiley, 1981), it seems that these designations carry important nomenclatural implications (Benton, 2000) and may impede the integration and interpretation of fossil and modern taxa by identical means. Where ancillary assumptions regarding local polarities and implications of ‘stem-group’ members are made in analyses based on narrow samples of taxa (e.g. Bourdon, 2005; Bourdon *et al.*, 2005) or characters (e.g. Mayr, 2002a, 2003a, b, c, 2004e, 2005i; Mayr & Clarke, 2003; Mayr *et al.*, 2003; Mayr & Ericson, 2004), or if the fossil material is of marginal quality (e.g. Mayr, 2002c, 2004e, 2005f), the differences between neontological and palaeontological schools can be substantial. In many contexts, it appears virtually inescapable that ‘stem-group’ status implicitly conserves the notion of possible or likely ancestry relative to the corresponding crown-group, and thereby suggests an evolutionary role beyond mere cladistic position (e.g. successive sister-groups).

Moreover, inclusion of a fossil in a ‘stem-group’ (Mayr, 2002c, 2005d) can lead to alternative analytical protocols, e.g. speculations of local polarities and substitution of hypothesized instead of observable character states to lend support to trees including mulitple fossils (e.g. Mayr, 2002c, 2004f, 2006a). The comparatively well known Pseudasturidae – formerly assigned to the Family Quercypsittacidae (Psittaciformes) by Mourer-Chauviré (1992) – were judged to combine ‘intermediacy’ in a number of characters purportedly diagnostic of psittaciforms with similarities to the ‘Galbulae’ (Piciformes), and were referred to the ordinal ‘stem-group’ of Psittaciformes by Mayr (2002c). Informal hypotheses of polarities in analyses of fossil birds – e.g. by Mayr (2005i: characters 5 and 12), Mayr *et al*. (2003: characters 1, 6, 11, 29, 35 and 71), and Mayr & Ericson (2004: character 55) – evidently intended to impose ‘local’ initial states in a particular context and often asserted in character descriptions (Mayr, 2002c), are innocuous if these are inferred from direct analysis rather than imposed based on preconceptions regarding a taxon. Evidently, however, in some cases states observed for given terminal taxa are replaced by states purportedly representative of ‘stem-group’ members (i.e. states hypothesized to be predecessors to those of taxa included in the corresponding ‘crown group’). Examples of the latter – confirmation of which requires character descriptions and the data matrix – include characters 5, 9, 18, 26, 30 and 68 of Mayr *et al*. (2003).

A related tradition of avian palaeontology is the imposition of intuitive trends that exceed the strict empirical content of available fossil material. For example, newly discovered fossils – notably a ‘stem-group hummingbird’ (*Eurotrochilus inexpectatus*) from the early Oligocene of Germany of purportedly modern grade – motivated Mayr (2003c, 2004d, 2005a, g) to enter the debate concerning the relationships of the Apodiformes. These efforts included a critique (Mayr, 2001f) of a description of a fossil taxon by Dyke (2001c), the attribution by Mayr (2004d) (based on limited taxonomic comparisons) of morphological specializations both for hovering flight and for nectarivory to *Eurotrochilus*, and speculations on the earliest evidence of avian nectarivory and the coevolution of certain bird-pollinated angiosperms in the New World. Given the oversimplification of distributions of characters, especially within the Apodiformes (Cohn, 1968; Karhu, 1992, 1999, 2001), and the wider controversy based on molecular data (Dumbacher *et al.*, 2003; Thomassen *et al.*, 2003, 2005; Chubb, 2004b), it is unfortunate that the characters included by Mayr (2001f, 2003c, 2004d, 2005g) totalled from 25 to 98, and failed to provide a synthesis of all relevant characters was not provided for relevant taxa prior to speculating regarding graded specializations and coevolutionary trends in the early Cenozoic.

#### Plesiomorph or interordinal ‘intermediate’?

Perhaps the most prevalent idiosyncracy of palaeontological perspectives is the reputed importance of fossils as a source of phylogenetic ‘bridges’ between extant, comparatively divergent lineages (Mayr, 2006b). However, neither the published record nor phylogenetic theory supports this notion, and the role of interordinal ‘linking’ lineages is at least as often revealed by extant taxa (Livezey, 1997a). The taxonomic history of *Presbyornis* illustrates the potential that such expectations may hold for phylogenetic placements of fossils with respect to modern higher-order groups.

Wetmore (1926) originally described *Presbyornis* from a single element from the Eocene of western North America as a charadriiform, but later (with abundant additional material) it was asserted to be a ‘transitional’ shorebird and indicative of a close relationship between Charadriiformes and Anseriformes (Olson & Feduccia, 1980a), the intuitive methods employed in the latter being criticized by Raikow (1981). More than a decade later and based on direct cladistic analysis of both *Presbyornis* and modern taxa, the genus was determined to be a plesiomorphic anatoid (Ericson, 1997; Livezey, 1997a). Subsequently, *Presbyornis* (and synonyms) has been the genus of choice for referral of fossils from the Eocene of England (Harrison & Walker, 1976a), Eocene of Mongolia (Kurochkin, 1988), Palaeocene of eastern North America (Olson, 1994; Ericson, 1997), Cretaceous of Antarctica (Noriega & Tambussi, 1995), late Palaeocene of North America (Benson, 1999), and Cretaceous of Mongolia (Kurochkin, Dyke & Karhu, 2002).

An examination of the material upon which these referrals were made raises reasonable doubts as to diagnostic reliability, and reveals the role of the comparatively well represented fossil *Presbyornis* as a palaeontological ‘strange attractor’ for other, variably preserved fossils of uncertain affinities. As the referrals of fossils to the early Anseriformes escalated, purported allies of *Presbyornis* also increased in number and morphological diversity: Olson (1994) reported a ‘giant’*Presbyornis* from the Palaeocene of eastern North America, Alvarenga (1999) referred a fossil from the mid-Tertiary of Brazil to the Anhimidae; Olson (1999b) phenetically allied *Anatalavis* from the London Clay to the modern Australian endemic Anseranatidae, a placement disputed by Dyke (2001b); Mourer-Chauviré*et al*. (2004) allied *Anserpica* from the Oligocene of Europe to the same family; and Clarke *et al*. (2005b) likened *Vegavis* (Cretaceous of Antarctica) to *Presbyornis* and referred the genus to the Anatoidea by a nested series of analyses of published data sets, by a method similar to that of supertrees.

The saga of *Presbyornis* also extended to the interordinal realm of fossil referrals, and provided insights into the alliance formerly alleged between *Presbyornis* and Phoenicopteridae by way of the poorly understood *Juncitarsus* (Olson & Feduccia, 1980a, b; Ericson, 1999), and thereby the subsequently proposed relationship between Phoenicopteridae and Podicipedidae. In addition, Cheneval & Escuillié (1992) cited similarities between grebes and the flamingo-like Palaelodidae in the pelvic appendage – the very class of characters considered by many of these authors to be prone to convergence and therefore unreliable in uniting grebes with loons.

Nevertheless, Mayr (2004c: 140) considered the sister-group relationship between grebes and flamingos to be ‘… one of the best supported higher-level clades within modern birds.’Mayr (2005a: 523) then suggested that the intermediacy of two skeletal features between *Juncitarsus* (Eocene of Wyoming) and the Palaelodidae (Oligocene of Europe), fossils traditionally allied to the Phoenicopteridae, ‘… provides a morphological link between Phoenicopteriformes and Podicipediformes.’ As for the early inferences made for *Presbyornis*, to which *Juncitarsus* and phoenicopterids were compared (Ericson, 1999), misclassification of fossils can lead to significant errors where informal phenetics and exceptional treatment of fossils are involved (Livezey, 1997a), problems not correctable by adoption of empirically depauperate taxonomic nomenclatures (e.g. ‘stems’ and ‘crowns’) and contradictory views on the phylogenetic roles of fossil taxa.

### Fossil neornithes: preservation and opportunities

#### Referrals, old and new

Despite the foregoing critique, well-preserved fossils can provide important insights into avian evolution, especially the Mesozoic origins of the group, and many potentially important fossils currently have yet to be described (J. A. Clarke, pers. comm.) and are beyond the scope of the present work. Unfortunately, a majority of fossil Neornithes, both of Mesozoic (Hope, 2002) and Cenozoic age (Brodkorb, 1963, 1964, 1967, 1971b), were named based on material not permitting meaningful inclusion in a formal cladistic analysis of modern scale. Moreover, classifications of many of these taxa were made phenetically, and with a marked tendency to refer new taxa to the modern taxon perceived to be most similar (Livezey & Martin, 1988; Livezey, 1997a, 2003c). Fortunately, increased use of cladistic analyses makes it likely that such records, especially those spanning the late Mesozoic and early Cenozoic, will provide an increasingly refined palaeontological dimension to avian phylogenetics.

Given the limitations of direct diagnosis (Table 2) and the phenetics of seeking the best neornithine group in which to place a fossil (Livezey & Martin, 1988; Livezey, 1997a), what is the recommended means for evaluation of a new fossil with respect to the present data set? Two paths seem most informative at present: (i) unconstrained analysis of the present data set, appended with the codings for the fossil taxon, however incomplete (within reasonable limits of informativeness); or (ii) analysis of the fossil taxon under a backbone-constraint for modern lineages (e.g. Figs 13–18). The latter probably will prove optimal in those cases where missing data are especially numerous or where even higher-order affinities are indiscernible, and especially where both circumstances pertain. Taxonomic groups of greatest diversity and quality of preservation hold the greatest potential for such insights, and these merit special emphasis here, especially those broadly consistent with groupings inferred here and for groups having few modern members.

#### Diversity, aquatic and terrestrial

Fossils have been referred, although not all by phylogenetic means, to all modern families of the Pelecaniformes: Phaethontidae (Harrison & Walker, 1976b; Olson, 1983b, 1985; Mayr & Smith, 2002), Fregatidae (Olson, 1977), Fregatidae or Sulidae (Olson & Matsuoka, 2005), Sulidae (Olson & Rasmussen, 2001; Mayr, 2002d; Stucchi & Urbina, 2004), Pelecanidae (Olson, 1999a), Phalacrocoracidae (Mayr, 2001c) and Anhingidae (Alvarenga, 1995; Alvarenga & Guilherme, 2003; Mourer-Chauviré*et al.*, 2004). In addition, the controversially referred Plotopteridae (Mayr, 2004b) have increased in palaeodiversity (Olson & Hasegawa, 1979, 1996; Olson, 1980; Goedert, 1988). Less well justified is the putative membership of a group of widespread, fossil, pseudo-denticulate birds – Odontopterygiformes (Owen, 1873; Howard, 1957; Goedert, 1989; Averianov *et al.*, 1991; Zusi & Warheit, 1992; González-Barba *et al.*, 2002) – for which a modest analysis mustered marginal support as an alternative sister-group to the Anseriformes (Bourdon, 2005).

The Coliiformes, Trogoniformes, Coraciiformes and Piciformes merit renewed examination as these groups (e.g. Bucconidae *sensu lato*, including Primobucconidae), as well as specimens of uncertain affinity (Olson, 1992b), also have received multiple new fossil referrals (Harrison, 1982a; Mayr, 2000c) – including Trogoniformes (Mourer-Chauviré, 1980; Mayr, 1998a, 1999a, 2001b, 2003b), Coliiformes (Olson & Houde, 1989; Houde & Olson, 1992; Mayr & Peters, 1998; Mayr, 2000b, 2001a, 2005d, e; Dyke & Waterhouse, 2000; Kristoffersen, 2001; Mayr & Mourer-Chauviré, 2004), Coraciiformes (Olson, 1976, 1992b; Mourer-Chauviré, 1985; Mayr & Mourer-Chauviré, 2000; Mayr, Mourer-Chauviré & Weidig, 2004b) and Piciformes (Mayr, 2001d, 2005h, i). Broadly delimited zygodactyl taxa (Feduccia & Martin, 1976; Mayr, 1998c, 2001e, 2004e, 2005h, i) complete the apparent palaeodiversity of ‘higher’ landbirds (Fig. 18), and contrasts with modern passeriform dominance (Manegold, Mayr & Mourer-Chauviré, 2004).

The Psittaciformes, at least the modern members of which are anatomically distinctive, have attracted a number of newly described fossils, some of which obscure this distinctness (Mayr, 2002c), and thus the order has undergone pronounced extensions of its palaeodistributional limits (Harrison, 1982b; Mourer-Chauviré, 1992; Mayr & Daniels, 1998; Stidham, 1998; Dyke & Mayr, 1999; Brochu & Norell, 2000; Dyke & Cooper, 2000; Mayr, 2001g, 2002c; Mayr & Göhlich, 2004; James, 2005). The uniquely apomorphic form of the crania of some taxa in this order is so extreme (Smith, 1975) as to pose challenges of comparability, and many modern members also manifest distinctly modified pectoral girdles and apomorphic pelvic skeletons (Smith, 1975; Livezey & Zusi, 2006). However, some fragments controversially referred to this clade are of potential relevance to the origins of modern orders and the K–T boundary (Stidham, 1998 vs. Dyke & Mayr, 1999), and merit reassessment.

### Spatiochronological dimensions of phylogenetics

#### Preservation and inferred distribution

A traditional referral of issues of ‘deep time’ to palaeontology (Brochu *et al.*, 2004) evidently reflects, in part, the rapidity with which fossil evidence was conjoined with modern phylogenetics for the calibration of geological time with phylogenetic hypotheses. Palaeocalibration of ages provided by fossil records in combination with models of molecular phylogenetics predictably turns on taxonomic groups possessed of rich, accurately aged fossils and reliable phylogenies.

These cross-disciplinary works progressed (perhaps too) rapidly toward attempts at global treatments of Neornithes that were influenced by undue inclusion of fossils of unreliable identity and age. In addition, the early spate of efforts favoured classes of models (e.g. Markovian) that facilitate minimization or ‘smoothing’ of discrepancies between calibrations and branching patterns as opposed to realistic incorporation of heterogeneous evolutionary rates (Sheldon *et al.*, 2000; Brochu & Norell, 2001; Van Tuinen & Hedges, 2001; Dyke, 2003; Pol *et al.*, 2004; Van Tuinen & Dyke, 2004; Van Tuinen *et al.*, 2006). The latter, often underappreciated, reality reflects the likelihood of preservation and a negative skewness of such records expected to be inversely correlated with body size and related heteroscedasticity that is directly correlated with geological age. These palaeontological issues are confounded by unrealistic assumptions of molecular trees and models in which the fossil data are incorporated. Not surprisingly, informativeness of such exercises to date has been limited – i.e. modern orders have been inferred to have very early origins (Pereira & Baker, 2006a: table 1; Van Tuinen *et al.*, 2006: tables 1, 2). Nevertheless, a phylogenetic hypothesis of high support and resolution (Figs 10–18) is an essential starting point – one, however, conditional on independent testing and augmentation. Another precondition of success, aside from well-documented fossil records (e.g. Clarke *et al.*, 2003), is use of realistic assumptions regarding molecular evolution where calibration of ages of divergence events is among the objectives (Pereira & Baker, 2006a).

#### Calibration of time

Failure to verify the existence of a molecular ‘clock’ notwithstanding (García-Moreno, 2004), an endeavour of particular interest regards bringing to bear the calibration of geological time – the ‘time axis’ of Benton (1996) – through phylogenetically placed fossil taxa, thereby estimating a minimal age of corresponding nodes in a phylogeny and recavering the temporal pattern of avian diversification (Hedges *et al.*, 1996; Mindell *et al.*, 1996; Miyaki *et al.*, 1998; Cooper & Penny, 1997; Kumar & Hedges, 1998; Sepkowski, 1999; Waddell *et al.*, 1999; Cracraft, 2001). Direct use of stratigraphic data for inference of trees by means of parsimony or ‘stratocladistics’ has been criticized on several methodological grounds, and is especially inappropriate for the sparse avian fossil record (Fisher, 1992; Huelsenbeck & Rannala, 2000).

Well-supported phylogenies for molecular models are critical for extrapolations of evolutionary rates from fossil-based point-estimates of geological age (Marshall, 1990; Springer, 1995; Arbogast *et al.*, 2002; Broham *et al.*, 2002; Smith & Peterson, 2002; Broham, 2003; Brochu, Sumrall & Theodor, 2004; Van Tuinen & Hedges, 2004). Disagreements among calibrations to date are consistent with evidence for significant variation among rates of evolution (Thorne, Kishno & Painter, 1998; Johnson & Cicero, 2004; Cicero & Johnson, 2006; Zink & Klicka, 2006), the effects of outgroups (Waddell *et al.*, 1999), initial estimates of which (e.g. Shields & Wilson, 1987) continue to be used. Other problems stem from the limited suitability of stratigraphic data in phylogenetic contexts (Huelsenbeck & Rannala, 2000), and effects of topological aspects of trees (Pol *et al.*, 2004). The continued controversy concerning the position of the Passeriformes relative to other Neoaves (especially Fig. 10) – considered by many to reflect effects of outgroup and relative evolutionary rates of mtDNA – presents a critical issue for attempts at calibrations more precise than Mesozoic vs. Cenozoic origins (Stanley & Cracraft, 2002; Cracraft *et al.*, 2004; Pereira & Baker, 2006a, b; Slack *et al*., 2006a).

Accordingly, the prospect of using currently available palaeontological data to calibrate evolutionary rates is disconcerting, regardless of the phylogenetic framework conjoined, principally because of a paucity of fossils that are reliably classified and of precise age (Hope, 2002; Livezey, 2003c). However, the existence of avian lineages in the late Mesozoic has been substantiated directly by palaeontological evidence (Olson, 1992a; Dalla Vecchia & Chiappe, 2002; Grellet-Tinner & Norell, 2002; Schweitzer *et al.*, 2002). For example, the estimated origin of megapodiid galliforms in the Cretaceous (Pereira & Baker, 2006b) agrees well with estimates for the comparably ancient Anseriformes.

Special attention relates to the oldest fossil record for a member of the Neornithes, increasingly with respect to hypotheses of descent relative to massive faunal upheavals following the K–T boundary (Feduccia, 1977c, 1995; Olson & Feduccia, 1980a; Olson & Parris, 1987; Paton *et al.*, 2002, 2003). Despite considerable effort, few points of agreement among phylogenetic calibration of rates and fossil records have been achieved (Benton, 1999, 2001; Dyke & Mayr, 1999; Van Tuinen & Hedges, 2001). In part, disagreements reflect variable reliances on assumpitions of ‘clock-like’ molecular evolution (Helm-Bychowski & Wilson, 1986; Van Tuinen & Hedges, 2001; Van Tuinen & Hadly, 2004). The unrealistic assumption of ‘clock-like’ molecular change (Brochu *et al.*, 2004) has led to diverse means of ‘correction’ or analytical adjustments (Mooers & Harvey, 1994; Sanderson, 1997; Mindell *et al.*, 1998; Bleiweiss, 1998c; Ho *et al.*, 2005), increased sampling of fossils (Springer, 1995; Smith & Peterson, 2002; García-Moreno, 2004; Pereira & Baker, 2006a, b), incorporation of multiple ‘clocks’ (Van Tuinen & Dyke, 2004) and relaxation of estimators through Bayesian methods (Yang & Rannala, 2006).

For example, Mayr (2002c) stated that the earliest passeriform is no older than the early Oligocene, whereas Cracraft *et al*. (2004) inferred the order to have originated prior to the K–T boundary, a discrepancy of magnitude likely to weaken associated calibrations. Recent attempts to bracket times of avian cladogenesis by Dyke & Van Tuinen (2004: fig. 3) based on the few widely accepted higher-order relationships necessarily encompassed relatively few major lineages of birds, whereas a priority accorded expanded taxonomic samples led Van Tuinen *et al*. (2006) to accept calibrations based on many fossils classified from the literature, relationships derived from the phenetics of DNA hybridization, and a null model incorporating questionable assumptions concerning molecular evolution (Pereira & Baker, 2006b) and a basal polytomy for Neoaves.

#### Palaeobiogeography and the spatial dimension

There is considerable optimism bestowed upon fossil taxa for the reconstruction of historical biogeography (Olson, 1985; Carroll, 1997). Southern-hemispheric patterns interpreted in terms of tectonic fragmentation and movements are manifested in the literature of avian systematics (Glenny, 1954; Cracraft, 1973b, 1975, 1976c, 1982c; Hedges *et al.*, 1996; de Kloet & de Kloet, 2005). Realistic reconstructions of historical biogeography require effects of vicariance events within continents – e.g. mountainous uplifts or glaciation – as a secondary class of abiotic antecedants of phylogenetic diversification (Ploeger, 1968; Cracraft, 1982c, d).

Of greater empirical substance for Aves, perhaps, are inferences of historical vicariance, notably those fortified by robust phylogenetic analyses and showing congruent geographical patterns. Most important of these for birds is the recurrent pattern of southern origins among many lineages, collectively suggestive of a critical role for Gondwana in early avian origins and diversification and most strikingly coincident with the K–T boundary (Cracraft, 2001). Patterns consistent with southern genesis are especially compelling in light of a biased tendency for migratory habit to counter northern–southern hemispheric patterns relative to those of eastern–western hemispheres (Böhning-Gaese, González-Guzmán & Brown, 1998). Taxa for which circumstantial evidence of this kind is consistent with southern-hemispheric origins (Cracraft, 1973b), include: Ratitae (Cracraft, 1974a; Haddrath & Baker, 2001), Anseriformes (Livezey, 1986, 1997a, 1998a), Galliformes (Dyke *et al.*, 2003), Sphenisciformes (Cracraft, 1988; Cracraft & Mindell, 1989; Harrison *et al.*, 2004), Gruiformes (Cracraft, 1973a, 1982b; Livezey, 1998b), Psittaciformes (Cracraft, 1988; Cracraft & Mindell, 1989; Miyaki *et al.*, 1998; de Kloet & de Kloet, 2005), Trochilidae (Bleiweiss, 1998d) and suboscine Passeriformes (Ericson, Johansson & Parsons, 2000; Irestedt *et al.*, 2001, 2002; Ericson *et al.*, 2002a, b, 2003b; Barker *et al.*, 2002, 2004; Edwards & Boles, 2002; Yuri & Mindell, 2002; Ericson & Johansson, 2003; Chubb, 2004b).

The Palaearctic understandably dominated palaeogeographical hypotheses in the early 20th century, especially for fossils from the late Cenozoic (Ploeger, 1968). The prevalence of terrestrial groups during the Palaeogene considered ‘basal’ to the Passeriformes (i.e. branching from the lineage culminating in the Passeriformes and its sister-group) prompted Mayr (2005a) to suggest that the former taxa may have occupied ‘passeriform’ niches prior to the Oligocene. This hypothesis should be amenable to testing by morphological comparisons but is contingent on the resolution of debated dates of origin of the Passeriformes (Boles, 1995, 1997; Cracraft *et al.*, 2004; Mayr & Manegold, 2004). Among the avian clades most frequently cited with respect to adaptive radiation, key innovation, ontogenetic underpinnings and sheer diversity – phenomena of prime interest (Starck, 1969; Smith, 1994) – are the Apodiformes and Passeriformes. Accordingly, the Apodiformes (especially the Trochilidae) attracted substantial anatomical (Cohn, 1968; Karhu, 1992, 1999, 2001) and phylogenetic study (Dyke, 2001c; Mayr, 2001f, 2003c, 2004d, 2005a, g; Thomassen *et al.*, 2003, 2005; Chubb, 2004b). The Passeriformes, however, hold a position of unique diversity – comprising 60% of extant Aves (Cracraft *et al.*, 2004) and unmatched global distribution (Fitzpatrick, 1988; Kochmer & Wagner, 1988), evolutionary success (Raikow, 1986, 1988; Vermeij, 1988) and adaptation (Baum & Larson, 1991).

#### Evolutionary radiations and the K-T controversy

Such palaeogeographical patterns have shed light on the theory of ‘adaptive radiation’ (Gould & Eldredge, 1977; Eldredge & Cracraft, 1980; Levinton, 1988; Eldredge, 1989; Valentine, 1990; Jablonski, 2000; Schluter, 2000), ‘explosive radiation’ (Feduccia, 1980, 1995, 1996, 2003; Sheehan & Fastovsky, 1992; Cooper & Penny, 1997; Kardong & Zweers, 1997; Cooper & Fortey, 1998), and ‘mass extinction’ (Jablonski, 2005) of Aves around the K–T boundary. Other biogeographical hypotheses of significance relate cladogenetic patterns and faunal diversity to tectonic movements (Hedges *et al.*, 1996; Craw, Grehan & Heads, 1999; Humphries & Parenti, 1999), and trans-Gondwanan dispersal (Cracraft, 1973b, 1975, 1976c, 1982b, c, 2001).

In particular, the Charadriiformes have been the focus of substantial, speculative scenarios regarding a special evolutionary role involving multiple avian groups and major extinctions. The notion that ‘shorebirds’ are fundamental to an understanding of avian evolution across the K–T boundary (Olson & Feduccia, 1980a, b) is no longer considered promising, and was based in part on a preconception of Charadriiformes as phenotypic intermediates bridging higher-order avian groups (Zweers & Vanden Berge, 1997a, b; Zweers, Vanden Berge & Berkhoudt, 1997; Dyke *et al.*, 2002; Paton *et al.*, 2002).

Quantitative estimation of rates of evolutionary change (Rodriguez-Trelles, Tarrio & Ayala, 2002) – given robust phylogenies (Marshall, 1990) and adequate fossil records (Sepkowski, 1999) – have fostered more detailed hypotheses of phylogenetic bottlenecks and ‘explosive’ radiation near the K–T boundary (Feduccia, 1995, 2003; but see Stanley & Cracraft, 2002). However, there is growing evidence, at least based on Bayesian analyses of data largely or entirely from the mitochondrial genome, that most or all neornithine orders date from the late Cretaceous (Grellet-Tinner & Norell, 2002; Schweitzer *et al.*, 2002; Dalla Vecchia & Chiappe, 2002; Pereira & Baker, 2006b; Slack *et al.*, 2006a, b; Van Tuinen *et al.*, 2006). If accurate, despite the vulnerability of such data to suboptimal rooting, this record undermines early anticipations of K–T boundary effects in modern orders and an evolutionary timespan in which major divergences of neornithine lineages would extend through the early and middle Cenozoic. Expectations for avian fossils of such antiquity are correspondingly conservative, and although fossils of such age potentially offer new calibration points for early avian lineages, there is diminished hope for points of calibration bearing on the relative antiquity of modern (super)orders of birds or precise molecular estimates of associated evolutionary rates characteristic of phylogenetic lineages.

### Priorities for future investigation

#### Current points of irresolution

Based on the present analysis (Figs 1–8) and other studies during recent decades (Figs 10–18), the area of primary ignorance for avian phylogenetics is the heretofore refractory groupings within the Neoaves, with principal problems being the highest-level nodes (notably the position of the Passeriformes within this group) and the comparatively routine but significant work of phylogeny within orders and families (Fig. 11; Appendix 1). Optimism remains justified, however, with new genes and molecular signal of scale higher than simple sequences and indels under exploration (e.g. retroposons). Reliance solely on a single scale of homology, e.g. the indels upon which Fain & Houde (2004) proposed largely hemisperically concordant ‘Metaves’ and ‘Coronaves’, is herein inferred to be nomina nuda (Appendix 1). This fixation on single-scale molecular analyses justifiably led Harshman *et al*. (2006: 42) to ask: ‘Can four million bases [nucleotides] resolve the [avian] tree?’ Fortunately, there are a number of anatomical fields of study that remain virtually untouched in modern phylogenetic contexts (Livezey & Zusi, 2001, 2006), a circumstance of hope in light of the palaeontological discoveries that will necessitate refinement of these and refined anatomical complexes coded (e.g. os palatinum of *Archaeopteryx*; Mayr *et al.*, 2005; Zusi & Livezey, 2006).

Several episodes of avian phylogeny were incompletely resolved in the present analysis (Figs 12–18), and will require significantly intensified sampling of taxa to solve:

- Positions of Aepyornithiformes and Dinornithiformes relative to extant ratites (Fig. 13).
- Resolution of genera within the Phasianoidea (Fig. 13).
- Resolution of the positions of the Psittaciformes and Columbiformes (Figs 16, 17).
- Determination of the relationships among several poorly resolved nodes involving the traditional Charadriiformes and Gruiformes, within the ‘central’ Charadriiformes (Fig. 15), for which alternative proposals continue to appear (Simmons *et al.*, 2004; Van Tuinen *et al.*, 2004; Paton & Baker, 2006; Pereira & Baker, 2006a), a task likely to require inclusion of rich suites of such integumentary characters as the natal integument for reconstruction of deeper nodes, and aspects of the definitive externum for resolution of shallower nodes (Jehl, 1968, 1971; Livezey, 1991, 1995b, c, d, 1996a, b, c, 1997c).
- Confirmation of relationships of the families of Caprimulgiformes (Fig. 17).
- Resolution of the trichotomy among the Coraciiformes, Piciformes and Passeriformes (Figs 17, 18), and resolution of subordinal and familial phylogeny within the Passeriformes, and affirming the position of the Passeriformes relative to other Neoaves.
- Make available an empirically grounded platform for finer-scale analyses of single orders or families of Neornithes as an alternative to the classical literature or the ‘tapestry’ by Sibley & Ahlquist (1990), with priority accorded to comparatively old, multifamilial orders (e.g. Galliformes, Procellariiformes) or traditionally challenging groups (e.g. Pelecanimorphae).
- Phylogenetic integration of well-preserved fossils into the phylogeny, both serving as additional taxa for resolution or revision of groups and as points of calibration of (minimal) ages of lineages of which these are members.

An especially rewarding class of study awaits optimization of life-historical attributes at the present phylogenetic scale, attributes such as sexual dimorphism, parental care and reproductive parameters (Wyles, Kunkel & Wilson, 1983; Winkler & Sheldon, 1993; Wesolowski, 1994; Wimberger & de Queiroz, 1996; Figuerola, 1999; Geffen & Yom-Tov, 2001; Tullberg, Ah-King & Temrin, 2002; Roulin, 2004; Pereira & Baker, 2005; Ekman & Ericson, 2006) as a starting point for more detailed studies in evolutionary biology. This area of study can advance only with: (i) use of well-resolved, robustly supported phylogenies, often not feasible (e.g. Cubo, 2003); and (ii) refinement of methods for optimization a posteriori of attributes, including where phylogenies include polytomies (Saunders, Smith & Campbell, 1984; Temrin & Sillén-Tullberg, 1994, 1995; Omland, 1997a, b; Ligon, 1999; Richardson *et al.*, 1999).

An unfortunate aspect of such methods has been revealed by a number of optimizations that relied on the phenetics of Sibley & Ahlquist (1990), ostensibly as it was the only hypothesis of adequate taxonomic breadth for the desired survey (e.g. Van Tuinen *et al.*, 2006). Attributes so assessed include body mass (Maurer, 1998), wing length (McCall, Nee & Harvey, 1998) and correlates of flightlessness (Cubo & Arthur, 2001). Most such published surveys have recovered significant patterns in selected morphological attributes despite the virtually universal view that the quasi-phylogeny that was used is unreliable. This incongruity indicates that apparent significance of optimizations is essentially meaningless, but more importantly provided a fortuitous insight that statistically significant patterns can emerge from inaccurate phylogenetic hypotheses and that it may be prudent to adopt more conservative critical values for tests of this nature. Until more discriminating methods are available, significance in this context should not be assessed against a null model of random change but instead against randomized evolution with varied descent or reserved for comparisons between phylogenies.

#### Broadened phylogenetic horizons

Philippe & Laurent (1998) entitled their paper with a challenge of undisputed cogency: ‘How good are deep phylogenetic trees?’ An expansion of phylogenetics of the Theropoda and Dinosauria is well underway, however, and will be central to a robust foundation for avian phylogeny, including significant implications for ‘global’ homology and anatomical nomenclature. This exploration should lead to phylogenetic hypotheses among Vertebrata of increasing scale, especially in light of character analyses already accomplished for non-avian Tetrapoda (Benton & Clark, 1988; Evans, 1988; Nielsen, 1995; Zardoya & Meyer, 1996; Laurin & Reisz, 1997; Philippe & Laurent, 1998; Zardoya *et al*., 1998; Xia, Xie & Kjer, 2003; Suzuki, Laskowski & Lee, 2004; Hill, 2005). Similar explorations among deep roots by molecular and morphological means also hold promise for the phylogenetic resolution of an expanded ‘super-clade’ of Reptilia and allied Tetrapoda (Benton, 1990; Graybeal, 1994; Kumazawa & Nishida, 1995; Mindell *et al.*, 1999; Ruta, Coates & Quicke, 2003), including (sub)fossil taxa to the degree permitted by remains (Handt *et al.*, 1994; Taylor, 1996) and logistic limits on life-historical data available for fossil taxa. In contrast to issues of quality of the fossil record (Wagner, 2000a) and limits on signal recoverable from fossils (Wagner, 2000b), potential for neontological study remains underexplored, especially that involving soft-tissue anatomical systems (Wägele, 1995).

In light of the evident attraction of probabilistic reconstructions, phylogenetics may benefit most from an expansion of Bayesian methods to address problems of incomplete data (Gelman & Xiao-Li, 2004), robustness of estimates (Insua & Ruggeri, 2000) and refined optimization, including (quasi-)likelihood methods, both parametric and non-parametric (Heyde, 1997; Beiko *et al.*, 2006; Anisimova & Gascuel, 2006). In both major classes of probabilistic models, renewed attention is justified to the analytical properties of branching processes (Harris, 1963; Athreya & Jagers, 1997; Kimmel & Axelrod, 2002; Haccou, Jagers & Vatutin, 2005), for which statistical methods have been elaborated only recently. In a problem of this unprecedented scale, it is critical for modern systematists to exploit a diversity of sources of data as a means to effect even-handed assessments of historical pattern.

An overview of the literature (Figs 1–10) reveals that much remains to be accomplished in avian phylogenetics. Significant advances principally lie in studies of great taxonomic scale and diverse support that target nodes of ordinal and higher taxonomic scales of Neoaves, in conjunction with a solution of the persistent disputes among morphological, mitogenomic and nuclear findings. In combination with incorporation of additional, evolutionarily conservative characters of soft anatomy (Oliveira *et al.*, 2004) and karyotypes (Shetty, Griffin & Graves, 1999; Burt, 2002), the methods of ‘total-evidence’ analyses hold promise for phylogenetic scales and calibration of ages previously not feasible (Stanley & Cracraft, 2002; Baker & Gatesy, 2002; Cracraft *et al.*, 2004; Yang & Rannala, 2006), a potential not without early tests (e.g. Kennedy & Page, 2002) and new methodological challenges (Baker *et al.*, 1998; Ballard *et al.*, 1998; Bang, Schultz & DeSalle, 2002; Bininda-Emonds *et al.*, 2002).

## APPENDIX 1

The following proposal for a higher-order classification of Class Aves is intended to encode natural groups as recovered in the foregoing phylogenetic analysis of the companion morphological data (Livezey & Zusi, 2006). Procedures of phylogenetic classification followed Wiley (1981) and Cracraft (1974b, 1978). We avoided the current divergence between rank-free Phylocode and traditional Linnean formats, as well as the palaeontological penchant for ‘stem’ and ‘crown’ groups. The four principles considered here were: (i) hierarchical grouping by phylogenetic relationship; (ii) preference to familiar, available taxa; (iii) preference given to names based on included type genera, where all other considerations are equal; and (iv) coordination of taxonomic ranks by similar emendation of names. Higher-order group names were chosen to conform most closely with others published comparatively recently and conformal with several conventions: (i) *incertae sedis* (indicative of unconfirmed monophyly and/or content); and (ii) *sedis mutabilis*, where a taxon comprises three or more members of equal rank (i.e. lineages in polytomy). Among Neornithes, the sequencing convention (Wiley, 1981) was used only for ordinal ranks for some taxa traditionally considered to be Gruiformes.

The comparatively simplified phylogeny upon which this classification is based is depicted in Figure 11. Exemplary taxa (often nominate genera) actually coded and analysed are shown explicitly in trees (Figs 13–18), and the higher-order taxa (mostly families) that correspond to the exemplars appear in the following classification. Families included in higher taxa are limited largely to those represented by exemplars analysed, e.g. two subfamilies of Anatidae as opposed to all recognized by Livezey (1997b). This convention is most notable with respect to the exceptionally diverse and minimally represented Passeriformes and embracing superorder. However, inclusion of comparatively recently recognized family group names within two orders of Superorder Psittacimorphae (Psittaciformes and Columbiformes), not represented among exemplars, was intended to counter under-representation of non-passeriform clades as well as to accommodate uncertainty of phylogenetic placement of exemplary genera with respect to recognized (sub)families.

No protocol for derivation of taxa of higher rank has been codified; a recent attempt was that by Sibley *et al*. (1988, 1990). The proposal made here is but one of many alternatives, including 150 years of provisional classifications. Although the ‘sequence convention’ might be applied to the very highest taxonomic ranks, we elected to retain distinct, dichotomous taxa to draw attention to these highest-order ranks within Neornithes; this permits hierarchical clarity, but it also results in some redundancy of higher-order names (Table 2). However, the convention was applied to names for some ranks listed prior to the Neornithes – parvclasses, sections, etc. (cf. Ratitae). We chose to use historical names over proliferation of new (semi)synonyms to preserve taxonomic history and despite the fact that this perpetuated some names of variably different content and inappropriate etymology and diagnosis (as understood by the original authors of these taxa). In some cases, acceptable taxa for some highest-order ranks were not found, and in these few instances new taxa were proposed, e.g. Terrestrornithes, and hyphenates of several others.

Among several frequently cited, 19th-century authors of higher-order taxa, two –Rafinesque (1815) and Leach (1820) – were disqualified following the adjudication of most modern systematists (Bock, 1994). We avoided group names of strongly militaristic overtones, e.g. the ‘brigades’ and ‘legions’ of Gadow (1893). We found the compendia by Lambrecht (1933), Wetmore (1930, 1960), Mayr (1958), Storer (1960a, 1971b), Brodkorb (1963, 1964, 1967, 1971b, 1978), Sibley *et al*. (1988, 1990) and Sibley & Ahlquist (1972, 1990) to be critical for ascertainment of taxonomic authorships. Many taxa named by Stresemann (1959) or delimited by Verheyen (1961) also were adopted. Full citations of references for higher-order taxa were not included herein for the sake of brevity. For taxa of rank greater than ordinal, we adopted, where possible, the system of suffixes proposed by Sibley *et al*. (1988, 1990) and Sibley & Ahlquist (1990). Given the dubious comparability of taxonomic ranks of higher-order names, we elected to forego annotation of taxa of supraordinal rank with the conventional specification of ‘new rank’ in this proposal.

The higher-order names given in bold type reflect inferred groupings, and although beyond the ranks of pervue by the ICZN, we provide diagnostic and supportive characters for these taxa (Table 2; Livezey & Zusi, 2006), whether new or conserved from historical works. The latter basis was preferred for naming higher-order groups, with inexactitude of content conceded as in such names used by Clarke & Norell (2002). For example, the content of an established name (e.g. Ornithurae) implicitly is defined herein (i.e. *sensu* present study). Taxonomy bearing on outgroup taxa (i.e. those preceeding Neornithes) are considered especially tentative. A minor point of contention is the position of *Lithornis* (Houde, 1988), a relative to palaeognathous Neornithes, inferred to be the sister-group of Tinamidae by Clarke & Norell (2002) and Clarke (2004), but inferred to be the sister-group of Neornithes by Clarke & Chiappe (2001), Leonard *et al*. (2005) and the present analysis (Fig. 12). Should a sister-group relationship between *Lithornis* and modern palaeognathous taxa be favoured, **Panpalaeognathae** Gauthier and de Queiroz, 2001 is available for the clade comprising both groups. Use of the traditional, higher-order taxon **Carinatae** Merrem, 1813, was precluded by provisional monophyly of *Hesperornis* and *Ichthyornis* in the present study (see also Rees & Lindgren, 2005), avoiding as well the implication of the name with respect to secondary obsolesence or loss of the carina sterni among Neornithes (Livezey, 2003a).

Supraordinal names proposed herein were intended to follow the convention of seniority of taxa and (to a lesser degree) included type family, as is typical of lower-scale taxa. Three important higher-order synonyms are: **Neoaves**Sibley *et al.*, 1988, senior to **Plethornithes**Groth & Barrowclough, 1999 (availability questionable in present context), and distinct from **Eoaves**Sibley *et al.*, 1988. There are also, two taxa –‘Cracrafti’ and ‘Conglomerati’– informally proposed as alternatives by Slack *et al*. (2006a). Unused herein is the potentially useful higher-order name **Euornithes** Sereno, 1999. **Gaviomorphae** replaced **Colymbimorphae** (Gadow, 1893) by revision of the former type genus *Colymbus*. We also replaced the senior, unfamiliar **Dypsporomorphae** Ogilvie-Grant, 1898, with the more familiar derivation **Pelecanimorphae**. Similar reasoning led to the suppression of **Aetomorphae** (Huxley, 1864) by **Falconimorphae** (Seebohm, 1890), the latter derived from an included ordinal taxon, but junior to the less representative alternative of **Strigimorphae** (Wagler, 1830). Uniform emendation of superorders was not imposed herein for non-Neornithes. The importance of dichotomy among higher-order names of comparable rank for comparability with the phylogenetic tree resulted in redundancy of supraordinal names for some clades, e.g. **Subdivision Dendrornithes** comprises a single **Section Raptores**, which in turn comprises a single **Superorder Raptoromorphae**. Antiquity of historical, higher-order taxa often resulted in minor differences in content – e.g. **Anomalogonates**Garrod, 1874 optimally should exclude the Cuculiformes for consistency with the myological diagnosis implied by the name.

Further study will probably subdivide **Superorder Passerimorphae** so as to comprise the **Superorder Coracomorphae**Huxley, 1867 and **Superorder Passerimorphae** (Linnaeus, 1758), the latter to comprise Piciformes and Passeriformes (cf. Manegold, 2005). Within the Passeriformes, the most suspicious anomaly in the present analysis was that of *Menura*; broader samples may justify its transfer to the Passerida, and thereby the first subordinal taxon instead may comprise the Acanthisittidae (Barker *et al.*, 2002, 2004; Ericson *et al.*, 2002a, b), representatives of which were not available for analysis here.

Principally because of limitations on available specimens, delegation of ordinal rank to the extinct elephant-birds (Aepyornithiformes) was favoured marginally over inclusion at lower rank within the Struthioniformes. A detailed classification of Order Anseriformes, including fossil taxa, was presented by Livezey (1997b), and a preliminary classification of the traditionally delimited Gruiformes, significantly revised by the present analysis relative to that proposed by Livezey (1998b), which tentatively recognized monophyly of the traditional order. (Sub)fossil taxa are plausible candidates for inclusion as sequential sister-groups of Galloanseromorphae (Diatrymidae, Gastornithidae and Dromornithidae) or membership within the Galliformes (Sylviornithidae) or Anseriformes (e.g. Mourer-Chauviré & Balouet, 2005) and are included based on published description (e.g. Cracraft, 1968; Livezey, 1997a) and cursory examinations. Material essential for rigorous diagnosis is rare or lacking, but we considered provisional hypotheses to indicate groupings likely but as yet undemonstrated by formal analysis preferable in such cases to no inference presented at all.

**Subclass Avialae** Gauthier, 1986

[**Infraclass Alvarezsauria** (Bonaparte, 1991)]

**Infraclass Aves** Linnaeus, 1758

**Parvclass Palaeoaves; new name**

**Superorder Archaeornithes**Gadow, 1893

Order Archaeopterygiformes Fürbringer, 1888

Family Archaeopterygidae Huxley, 1872

Order Confuciusornithiformes (Chiappe *et al.*, 1999)

Family Confuciusornithidae Hou *et al.*, 1995

**Superorder Euenantiornithes** Walker, 1981; *incertae sedis*

Order Rahonaviformes; **new name**

Family Rahonavidae**; new name**

Order Apsaraviformes; **new name**

Family Apsaravidae**; new name**

**Parvclass Ornithurae** Haeckel, 1866

**Superorder Odontoholomorphae** (Stejneger, 1885)

Order Hesperornithiformes (Fürbringer, 1888)

Family Hesperornithidae Marsh, 1872

Order Ichthyornithiformes (Marsh, 1873)

Family Ichthyornithidae (Marsh, 1873)

**Parvclass Eoaves**Sibley *et al.*, 1988; *incertae sedis*

Order Lithornithiformes Houde, 1988

Family Lithornithidae Houde, 1988

**Parvclass Neornithes**Gadow, 1893

**Cohort Palaeognathae** Pycraft, 1900

**Subcohort Crypturi** Goodchild, 1891

**Superorder Dromaeomorphae** (Huxley, 1867)

Order Tinamiformes (Huxley, 1872)

Family Tinamidae Gray, 1840

**Subcohort Ratitae** Merrem, 1813

**[Superorder Apterygimorphae**; *incertae sedis*]

Order Apterygiformes (Haeckel, 1866)

Family Apterygidae Gray, 1840

Order Dinornithiformes (Gadow, 1893)

[Family Anomalopterygidae (Archey, 1941)]

[Family Dinornithidae (Owen, 1843)]

**Superorder Casuariimorphae; new taxon**

Order Casuariiformes (Forbes, 1884)

Family Casuariidae Kaup, 1847

Family Dromaiidae Richmond, 1908

**Superorder Struthionimorphae; new taxon**

Order Aepyornithiformes (Newton, 1884)

Family Aepyornithidae Bonaparte, 1853

Order Struthioniformes (Latham, 1790)

Family Struthionidae Vigors, 1825

Family Rheidae (Bonaparte, 1853)

**Cohort Neognathae** Pycraft, 1900

**Subcohort Galloanserae**Sibley & Ahlquist, 1990

**Superorder Galloanserimorphae** (Sibley *et al.*, 1988)

Order Galliformes (Temminck, 1820)

Suborder Craci Sibley *et al.*, 1988; *incertae sedis*

Superfamily Megapodioidea (Lesson, 1831)

Family Megapodiidae Lesson, 1831

[Family Sylviornithidae Mourer-Chauviré & Balouet, 2005]

Superfamily Cracoidea (Vigors, 1825)

Family Cracidae Vigors, 1825

Suborder Phasiani (Vigors, 1825)

Superfamily Meleagridoidea (Gray, 1840)

Family Meleagrididae Gray, 1840

Superfamily Phasianoidea (Vigors, 1825); *sedis mutabilis*

Family Phasianidae (Vigors, 1825); *sedis mutabilis*

(Sub)Family Tetraonidae Vigors, 1825

Subfamily Perdicinae (Bonaparte, 1838)

Subfamily Odontophorinae Gould, 1844

Subfamily Phasianinae Vigors, 1825

Subfamily Numidinae Reichenbach, 1850

[Order Dromornithiformes Fürbringer, 1888]

Family Dromornithidae Vigors, 1825

[Order Diatrymiformes (Shufeldt, 1913)]

Family Diatrymidae Shufeldt, 1913

Order Anseriformes (Wagler, 1831)

Suborder Anhimae Wetmore & Miller, 1926

Family Anhimidae Stejneger, 1885

Suborder Anseres Wagler, 1831

Superfamily Anseranatoidea (Sclater, 1880)

Family Anseranatidae Sclater, 1880

Superfamily Anatoidea (Vigors, 1825)

[Family Presbyornithidae Wetmore, 1926]

Family Anatidae (Vigors, 1825)

Subfamily Anserinae Vigors, 1825

Subfamily Anatinae (Vigors, 1825)

**Subcohort Neoaves**Sibley *et al.*, 1988

**Division Natatores** Baird, 1858

**Subdivision Pygopodo-tubinares; new taxon**

**Superorder Gaviomorphae; new taxon**

Order Gaviiformes Wetmore & Miller, 1926

Family Gaviidae Allen, 1897

Order Podicipediformes (Fürbringer, 1888)

Family Podicipedidae Bonaparte, 1831

**Superorder Procellariimorphae** (Fürbringer, 1888)

Order Sphenisciformes Sharpe, 1891

Family Spheniscidae Bonaparte, 1831

Order Procellariiformes Fürbringer, 1888

Suborder Pelecanoidi (Gray, 1871)

Family Pelecanoididae Gray, 1871

Suborder Procellarae (Gadow, 1893)

Superfamily Oceanitoidea (Huxley, 1868)

Family Oceanitidae Forbes, 1882

Superfamily Procellarioidea (Fürbringer, 1888)

Family Procellariidae Vigors, 1825

Subfamily Procellariinae (Vigors, 1825)

Subfamily Pachyptilinae (Oliver, 1930)

Family Diomedeidae Gray, 1840

**Subdivision Stegano-grallatores; new taxon**

**Superorder Pelecanimorphae**Huxley, 1867

[Order Odontopterygiformes (Spulski, 1910)]

Family Odontopterygidae Lambrecht, 1933

Order Balaenicipitiformes (Sclater, 1924)

Suborder Balaenicipites (Sclater, 1924)

Family Balaenicipitidae (Sclater, 1924)

Order Pelecaniformes Sharpe, 1891

Suborder Phaethontes (Sharpe, 1891)

Family Phaethontidae Brandt, 1831

Suborder Steganopodes (Chandler, 1916)

Infraorder Fregatides (Sharpe, 1891)

Superfamily Fregatoidea (Garrod, 1874)

Family Fregatidae Garrod, 1874

Infraorder Pelecanides (Sharpe, 1891)

Parvorder Pelecanida (Sharpe, 1891); **new rank**

Family Pelecanidae Vigors, 1825

Parvorder Sulida (Reichenbach, 1849); **new rank**

Superfamily Suloidea (Reichenbach, 1849)

Family Sulidae Reichenbach, 1849

Superfamily Phalacrocoracoidea (Bonaparte, 1854)

Family Phalacrocoracidae (Bonaparte, 1854)

Family Anhingidae Ridgway, 1887

**Superorder Ciconiimorphae** (Garrod, 1874)

Order Ciconiiformes Garrod, 1874

Suborder Scopi (Bonaparte, 1853)

Family Scopidae (Bonaparte, 1853)

Suborder Ciconiae (Bonaparte, 1874)

Superfamily Ciconioidea (Sundevall, 1836)

Family Ciconiidae Sundevall, 1836

Family Phoenicopteridae Bonaparte, 1838

Superfamily Threskiornithoidea (Richmond, 1917)

Family Threskiornithidae Richmond, 1917

Family Plataleidae (Bonaparte, 1838)

Order Ardeiformes (Wagler, 1831)

Family Cochleariidae Ridgway, 1887

Family Ardeidae Vigors, 1825

Subfamily Botaurinae Bock, 1956

Tribe Botaurini (Bock, 1956)

Tribe Tigriornithini Bock, 1956

Subfamily Ardeinae (Vigors, 1825)

Tribe Nycticoracini Bock, 1956

Tribe Ardeini Bock, 1956

**Division Terrestrornithes; new taxon**

**Subdivision Telmatorae** (Lowe, 1931)

**Superorder Charadriimorphae**Huxley, 1867

Order Gruiformes (Bonaparte, 1854)

Suborder Cariamae (Wagler, 1830)

Infraorder Otides Sibley *et al.*, 1988

Family Otididae Gray, 1840

Infraorder Cariamides (Fürbringer, 1888)

Superfamily Cariamoidea (Gray, 1853); *sedis mutabilis*

[Family Bathornithidae Wetmore, 1933]

Family Cariamidae Bonaparte, 1853

[Family Phorusrhacidae (Ameghino, 1899)]

Suborder Eurypygae (Fürbringer, 1888)

Infraorder Eurypygides Sibley *et al.*, 1988

Family Eurypygidae Selby, 1840

Infraorder Rhynochetides Sharpe, 1891

Family Rhynochetidae Newton, 1868

Family Aptornithidae Bonaparte, 1856

Suborder Grues Bonaparte, 1854

Superfamily Psophioidea (Bonaparte, 1831)

Family Psophiidae Bonaparte, 1831

Superfamily Gruoidea (Vigors, 1825)

Family Aramidae Bonaparte, 1854

Family Gruidae Vigors, 1825

Order Turniciformes (Huxley, 1868); *incertae sedis*

Family Turnicidae (Gray, 1840)

Family Mesitornithidae Wetmore, 1960

Order Ralliformes (Reichenbach, 1854)

Family Heliornithidae Gray, 1841

Family Rallidae (Reichenbach, 1854)

Order Charadriiformes (Fürbringer, 1888)

Suborder Pedionomae (Gadow, 1893)

Family Pedionomidae Gadow, 1893

Suborder Parrae (Gadow, 1893)

Family Jacanidae Stejneger, 1885

Family Rostratulidae Ridgway, 1919

Suborder Limicolae (Beddard, 1898)

Infraorder Dromaides (Sharpe, 1891)

Family Dromadidae Gray, 1840

Infraorder Scolopacides (Strauch, 1978)

Superfamily Thinocoroidea (Gray, 1845)

Family Thinocoridae (Gray, 1845)

Superfamily Scolopacoidea (Vigors, 1825)

Family Scolopacidae Vigors, 1825

Family Phalaropodidae Bonaparte, 1831

Infraorder Charadriides (Huxley, 1867); *incertae sedis*

Superfamily Charadrioidea (Vigors, 1825)

Family Charadriidae Vigors, 1825

Superfamily Glareoloidea (Brehm, 1831)

Family Glareolidae Brehm, 1831

Subfamily Glareolinae Brehm, 1831

Subfamily Cursoriinae Gray, 1840

Superfamily Burhinoidea (Mathews, 1912)

Family Burhinidae Mathews, 1912

Superfamily Haematopoidea (Bonaparte, 1838)

Family Haematopidae Bonaparte, 1838

Subfamily Haematopodinae (Bonaparte, 1838)

Subfamily Ibidorhynchinae Bonaparte, 1856

Family Recurvirostridae (Bonaparte, 1831)

Subfamily Recurvirostrinae Bonaparte, 1831

Subfamily Himantopodinae Reichenbach, 1849

Tribe Himantopodini Sibley *et al.*, 1988

Tribe Cladorhynchini; **new taxon**

Suborder Lari Sharpe, 1891; *incertae sedis*

Infraorder Chionidides Sharpe, 1891

Family Chionididae Lesson, 1828

Infraorder Alcides (Sharpe, 1891)

Family Alcidae (Vigors, 1825)

Infraorder Larides (Sharpe, 1891)

Superfamily Laroidea (Bonaparte, 1831)

Family Stercorariidae Gray, 1870

Family Laridae (Bonaparte, 1831)

Subfamily Larinae Bonaparte, 1831

Subfamily Sterninae Bonaparte, 1838

Superfamily Rynchopoidea (Bonaparte, 1838)

Family Rynchopidae (Bonaparte, 1838)

**Subdivision Dendrornithes** (Verheyen, 1961)

**Section Raptores** Baird, 1858

**Superorder Falconimorphae** (Seebohm, 1890)

Order Falconiformes Seebohm, 1890

[Suborder Teratornithi (Miller, 1909)]

Suborder Cathartae (Coues, 1824)

Family Cathartidae (Lafresnaye, 1839)

Suborder Accipitres (Vieillot, 1816)

Infraorder Serpentariides (Seebohm, 1890)

Family Sagittariidae Finsch & Hartlaub, 1870

Infraorder Falconides (Sharpe, 1874)

Superfamily Falconoidea (Vigors, 1824)

Family Falconidae Vigors, 1824

Subfamily Falconinae Vigors, 1824

Subfamily Polyborinae Lafresnaye, 1839

Family Pandionidae Sclater & Salvin, 1873

Superfamily Accipitroidea (Vieillot, 1816)

Family Accipitridae (Vieillot, 1816)

Subfamily Accipitrinae (Vieillot, 1816)

Subfamily Gypaetinae (Vieillot, 1816)

Order Strigiformes (Wagler, 1830)

Family Tytonidae (Mathews, 1912)

Subfamily Tytoninae Mathews, 1912

Subfamily Phodilinae Beddard, 1898

Family Strigidae (Gray, 1840)

**Section Anomalogonates**Garrod, 1874

**Subsection Coccyges**Huxley, 1867; *incertae sedis*

**Superorder Cuculimorphae**Sibley *et al.*, 1988

Order Opisthocomiformes (L’Herminer, 1837)

Family Opisthocomidae Swainson, 1837

Order Cuculiformes (Wagler, 1830)

Suborder Musophagi Seebohm, 1890

Family Musophagidae Bonaparte, 1831

Suborder Cuculi Wagler, 1830

Family Cuculidae Vigors, 1825; *sedis mutabilis*

Subfamily Neomorphinae Shelley, 1891

Subfamily Centropodinae Horsfield, 1823

Subfamily Crotophaginae Swainson, 1837

Subfamily Cuculinae (Vigors, 1825)

Subfamily Phaenicophacinae (Horsfield, 1822)

**Superorder Psittacimorphae** (Huxley, 1867); *incertae sedis*

Order Psittaciformes (Wagler, 1830); *sedis mutabilis*

Family Nestoridae (Bonaparte, 1850)

Family Psittacidae (Illiger, 1811)

Family Cacatuidae Gray, 1840

Family Loriinidae Selby, 1836

Order Columbiformes (Garrod, 1874)

Suborder Pterocletes (Boucard, 1876)

Family Pteroclidae Bonaparte, 1831

Suborder Columbae (Latham, 1790)

Family Columbidae (Illiger, 1811)

Subfamily Columbinae (Illiger, 1811)

Subfamily Didunculinae Gray, 1848

Subfamily Gourinae Gray, 1840

Family Raphidae Wetmore, 1930

**Subsection Incessores** Baird, 1858

**Superorder Cypselomorphae**Huxley, 1867

Order Caprimulgiformes (Ridgway, 1891)

Suborder Aegotheli Sibley *et al.*, 1988

Family Aegothelidae (Bonaparte, 1853)

Suborder Caprimulgi Ridgway, 1881; *sedis mutabilis*

Family Caprimulgidae Vigors, 1825

Family Nyctibiidae (Chenu & Des Murs, 1851)

Family Podargidae (Gray, 1840)

Family Steatornithidae (Gray, 1846)

Order Apodiformes Peters, 1940

Suborder Hemiprocni; **new taxon**

Family Hemiprocnidae Oberholser, 1906

Suborder Apodi (Peters, 1940)

Family Apodidae (Hartert, 1897)

Subfamily Cypselinae Bonaparte, 1838

Subfamily Apodinae Hartert, 1897

Family Trochilidae Vigors, 1825

**Subsection Trogones; new name**

**Superorder Trogonomorphae; new taxon**

[Order Sandcoleiformes Houde & Olson, 1992]

Family Sandcoleidae Houde & Olson, 1992

Order Coliiformes (Murie, 1872)

Family Coliidae (Swainson, 1836)

Order Trogoniformes Wetmore & Miller, 1926

Family Trogonidae Lesson, 1828

**Subsection Pico-clamatores; new name**

**Superorder Passerimorphae**Sibley *et al.*, 1988; *sedis mutabilis*

Order Coraciiformes Forbes, 1884

Suborder Bucerotes Fürbringer, 1888

Infraorder Upupides (Seebohm, 1890)

Family Upupidae Bonaparte, 1831

Family Phoeniculidae Sclater, 1924

Infraorder Bucerotides (Fürbringer, 1888)

Family Bucerotidae (Gray, 1847)

Suborder Halcyones (Forbes, 1884)

Superfamily Motmotoidea (Gray, 1840)

Family Motmotidae Gray, 1840

Superfamily Alcedinoidea (Stejneger, 1885)

Family Todidae Vigors, 1825

Family Alcedinidae (Bonaparte, 1831)

Subfamily Alcedininae Bonaparte, 1831

Subfamily Halcyoninae (Vigors, 1825)

Suborder Coracii (Forbes, 1884)

Infraorder Meropides (Fürbringer, 1888)

Family Meropidae Vigors, 1825

Infraorder Coraciides (Wetmore & Miller, 1926)

Superfamily Coracioidea (Vigors, 1825)

Family Coraciidae Vigors, 1825

Superfamily Leptosomatoidea (Bonaparte, 1850)

Family Leptosomatidae Bonaparte, 1850

Family Brachypteraciidae (Sharpe, 1892)

Order Piciformes (Meyer & Wolf, 1810)

Suborder Galbulae (Fürbringer, 1888)

Family Galbulidae Bonaparte, 1831

Family Bucconidae Boie, 1826

Suborder Pici (Meyer & Wolf, 1810)

Superfamily Capitonoidea (Bonaparte, 1840)

Family Capitonidae Bonaparte, 1840

Family Rhamphastidae Vigors, 1825

Superfamily Picoidea (Vigors, 1825)

Family Indicatoridae Swainson, 1837

Family Picidae Vigors, 1825

Subfamily Jynginae Bonaparte, 1838

Subfamily Picinae Bonaparte, 1838

Order Passeriformes (Linnaeus, 1758)

Suborder Menurae (Sharpe, 1891)

Family Menuridae (Lesson, 1828)

Suborder Passeres Linnaeus, 1758

Infraorder Tyrannides Sibley *et al.*, 1988

Family Tyrannidae Vigors, 1825

Family Pittidae Swainson, 1831

Infraorder Passerides (Linnaeus, 1758)

Parvorder Corvida Sibley *et al.*, 1988

Family Ptilinorhynchidae Gray, 1841

Family Corvidae Vigors, 1825

Parvorder Passerida Sibley *et al.*, 1988

Family Bombycillidae Swainson, 1831

Family Paridae Vigors, 1825

Family Passeridae Illiger, 1811
